# Decoding the Water Harvesting Mechanism of MIL-100(Fe) Across Short- and Long-Range Length Scales

**DOI:** 10.1021/jacs.5c12269

**Published:** 2025-10-28

**Authors:** Francesco Tavani, Alessandro Tofoni, Eva Pietropaoli, Dragos Constantin Stoian, Wouter van Beek, Kenneth Marshall, Ida Pettiti, Alessandro Latini, Paola D’Angelo

**Affiliations:** † Dipartimento di Chimica, Università degli Studi di Roma La Sapienza, P.le A. Moro 5, I-00185 Rome, Italy; ‡ Swiss−Norwegian Beamlines, European Synchrotron Radiation Facility, 71 Avenue des Martyrs, 38000 Grenoble, France

## Abstract

Metal–organic frameworks (MOFs) hold promise as designer materials for atmospheric water harvesting, due to their unrivaled porosity, chemical tunability, and water affinity. Although an accurate understanding of the pore filling sequence is critical to developing improved MOF water harvesters, obtaining molecular-level details of the evolution of water clusters in MOFs has proven to be experimentally challenging. Here, a novel approach based on X-ray absorption spectroscopy (XAS), X-ray pair distribution function, powder X-ray diffraction, molecular dynamics (MD) simulations and in-depth theoretical XAS calculations is presented to gain quantitative insights into the structural and dynamical properties of water adsorbed within MIL-100­(Fe), a prototypical MOF with giant pores. The complementary synchrotron X-ray techniques shed light on the behavior of water confined in MIL-100­(Fe) with unprecedented structural sensitivity at the short-, intermediate- and long-range length scales, while the MD and theoretical XAS simulations revealed the order according to which water molecules populate the MOF mesopores and tracked the evolution of the hydrogen-bond network topology as a function of water content. The developed method can provide often elusive information on how the local structure affects the behavior and performance of MOF water harvesters, which is key to the development of rationally optimized MOF systems.

## Graphical abstract

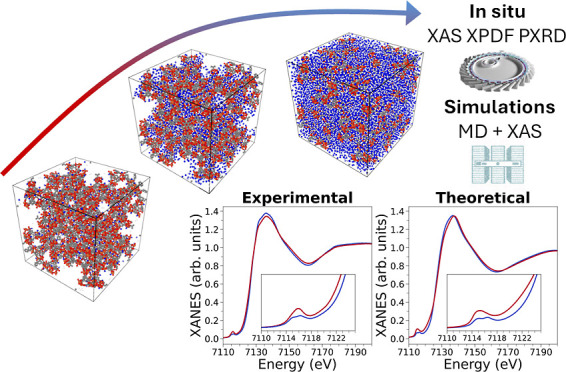

## Introduction

Humanity is on the verge of a major water crisis, with more than half of the world’s population expected to experience issues related to water scarcity by 2050.^1–4^ Metal–organic frameworks (MOFs), a class of porous solids that exhibit unprecedented porosity, chemical tunability, and ability to selectively “pluck out” molecules such as water from the atmosphere,^5–7^ have recently risen to the forefront of global efforts to address this societal challenge. MOFs may in fact be designed to exhibit high water uptake, long-term water stability, and fast sorption kinetics, characteristics that make these porous materials ideal for efficiently harvesting water from air even under desert conditions.^8–10^ MOF-based water harvesting devices have been shown to effectively harvest water from the atmosphere in both temperate and arid climates,^9–14^ while multivariate syntheses^8^ and emerging artificial intelligence methods^15^ have been employed to shape the water uptake and finely control the water adsorption behavior of state-of-the-art MOFs such as MOF-303 ^16^ and its isostructural analogues.^17^

These recent discoveries raise the question of how the MOF structural properties affect the water harvesting mechanism and performance, at the molecular level. Indeed, characterizing the structural details of the MOF water harvesting mechanisms at both short- and long-range length scales has been recognized as critical to designing MOF water harvesting systems with improved water productivity and regeneration temperatures.^16^ To date, techniques sensitive to global structural modifications such as neutron and X-ray diffraction have been applied to locate the positions of water guest molecules in the MOF pores^7,16,18–20^ and to understand the mechanism of how water-binding sites are populated,^16^ while computational methods such as molecular dynamics (MD) simulations have been used to provide an ensemble description of the MOF water harvesting process.^21–25^ However, accurate details on how the MOF local structural properties evolve as a function of water loading are often missing, since it is much harder to establish structure–property relationships around the metal sites as these require advanced experiments that reproduce realistic water harvesting conditions.

In this work, we employ for the first time complementary synchrotron X-ray techniques sensitive to short-, intermediate- and long-order ranges in combination with advanced theoretical methods to investigate the water harvesting process by MIL-100­(Fe), a prototypical water harvesting MOF with giant pores.^26^ In order to obtain accurate insights into the water absorption mechanism, we employed a combined approach using X-ray absorption spectroscopy (XAS),^27^ X-ray pair distribution function (PDF), and powder X-ray diffraction (PXRD) measurements. This innovative method allowed us to track the evolution of the structural properties of the water/MOF system at increasing water loadings across short- and long-range length scales with unprecedented sensitivity. MD simulations and ab initio theoretical calculations of the spectroscopic data were then employed to unveil the structural and dynamical properties of water confined in MIL-100­(Fe) and the changes in the topology of the hydrogen-bond network as a function of water content. Our work highlights how complementary X-ray techniques may be combined to gain an accurate understanding of both the local and higher distance structural properties of MOF water harvesters and paves the way for future studies leveraging synchrotron and computational methods to establish structure–property relationships for improved MOF performances.

## Materials and Methods

### Synthesis and Characterization of MIL-100­(Fe)

MIL-100­(Fe) was synthesized in gram quantities following a previously reported HF-free procedure^28^ with slight modifications. Phase purity and crystallinity of the final material were verified by PXRD. N_2_ physisorption measurements were carried out to determine the Brunauer–Emmett–Teller (BET) surface area of the sample, which was 1439 m^2^ g^–1^. Additional details on the synthesis and N_2_ adsorption characterization of the MIL-100­(Fe) sample are provided in Sections S1.1 and S1.2, of the Supporting Information, respectively.

### In Situ XAS-PXRD Experiments

The in situ XAS–PXRD-PDF measurements were performed at the BM31 beamline (Swiss-Norwegian beamlines, SNBL) of the ESRF. The XAS spectra were collected at the Fe K-edge continuously scanning between 7000 and 7900 eV with a step size of 0.3 eV. PXRD patterns were collected by means of a CdTe Dectris Pilatus3 2 M detector (λ = 0.25448 Å) and an acquisition time of 30 s. PXRD patterns of PDF quality were collected using a dedicated detector configuration with an acquisition time of 60 s, averaging a total of 10 scans for each protocol step. PDF profiles were then extracted with PDFgetX3.^29^ Full details on the in situ XAS–PXRD-PDF measurements and on the PXRD data pretreatment and analysis are provided in Section S1.3 of the Supporting Information.

### MD Simulations

All classical MD simulations were carried out at 25 °C by means of the LAMMPS code.^30^ The simulation cell and the coordinates of the MOF framework atoms used for all MD simulations were derived from the crystal structure available in the literature.^26^ In particular, unit cell parameters equal to 73.3402 Å (*Fd*3̅*m* space group) were employed. In all simulations the MOF structure was rigid and the simulation box comprised a single MIL-100­(Fe) unit cell. The parameters of the force field model for the interaction between the MOF framework and the water molecules were taken from the literature,^31^ while the water–water interactions were treated by means of the TIP4P-Ew model,^32^ as previously suggested.^31^ The framework flexibility was not considered in the MD calculations, as flexible framework calculations were found to worsen the agreement between the theoretical and experimental XAS spectra while also increasing the computational cost. Each system was first equilibrated in the constant volume and constant temperature (*NVT*) canonical ensemble at 25 °C for 1 ns with a 1 fs time step, while production *NVT* runs were performed for 10 ns. Dynamical information^33,34^ was obtained by means of 1 ns simulations in the constant volume and constant energy (*NVE*) microcanonical ensemble during which the temperature remained stable around 25 °C. Further details on the MD simulations and on the analyses carried out on the MD trajectories are provided in Table S2 and Section S1.4 of the Supporting Information, respectively.

### Ab Initio XAS Calculations

The Fe K-edge NEXAFS theoretical calculations were performed with the FDMNES code making use of the approximated muffin-tin potential in the calculations.^35–39^ The simulations of the XAS spectra obtained from the MD-extracted configurations were performed by considering the MOF framework atoms and water molecules within 6 Å of the photoabsorber in each calculation. For all calculations, we obtained the cluster potential by considering a neutral 3d^6^4s^2^ electronic configuration for iron without including magnetic interactions. At each water loading, theoretical XAS spectra were computed as averages of 94 distinct MOF trimeric units in the simulation box to account for slight fluctuations of the local structure around the Fe centers. A total of 118.440 individual theoretical XAS spectra were simulated for MIL-100­(Fe) at increasing water loadings. A rigid energy shift of 7114.7 eV was applied to all theoretical XAS spectra to allow comparison with the experimental data. Additional details are provided in Section S1.5 of the Supporting Information.

## Results and Discussion

### Structural Properties of MIL-100­(Fe)

MIL-100­(Fe) has been actively investigated for atmospheric water harvesting^40–42^ owing to its high chemical and hydrothermal stability, as well as to its large internal surface area arising from the giant pore framework.^26,43^ The crystal structure of MIL-100­(Fe) is built from triiron oxo-centered clusters and displays a cubic space group (*Fd*3̅*m*) with a lattice parameter of approximately 73.3 Å and a unit cell volume greater than 390,000 Å^3^.^26,44^ The Fe­(III) sites in the trimeric units are joined by BTC linkers (BTC^3–^ = benzenetricarboxylate) in a slightly distorted octahedral geometry, with two water molecules and a hydroxyl ion coordinating the metal sites in the apical positions. In particular, MIL-100­(Fe) features two types of mesopores with internal diameters of 25 Å and 29 Å, respectively, which exhibit pentagonally and hexagonally shaped pore windows (Figure 1a). Previous studies have highlighted that the water adsorption isotherm of MIL-100­(Fe) displays secondary uptake due to the dual pore structure of the MOF, with the adsorption occurring first in the smaller pores and subsequently in the larger pores.^40,44^ However, spectroscopic and diffraction methods have to the best of our knowledge not yet been applied to investigate the MIL-100­(Fe) water uptake, presumably due to the complexity of the MOF whose large unit cell makes it extremely difficult to extract reliable structural information through any individual technique alone. Here, MIL-100­(Fe) was synthesized following a green, HF-free synthetic protocol^45^ based on water reconstruction,^28,46^ which afforded a crystalline MOF sample whose phase purity was confirmed by a structureless Le Bail refinement (Figure S1). The N_2_ sorption profiles and corresponding calculated surface area of the MIL-100­(Fe) sample (Figure S2) were found to be in good agreement with those previously reported in the literature.^47,48^

**Figure 1:**
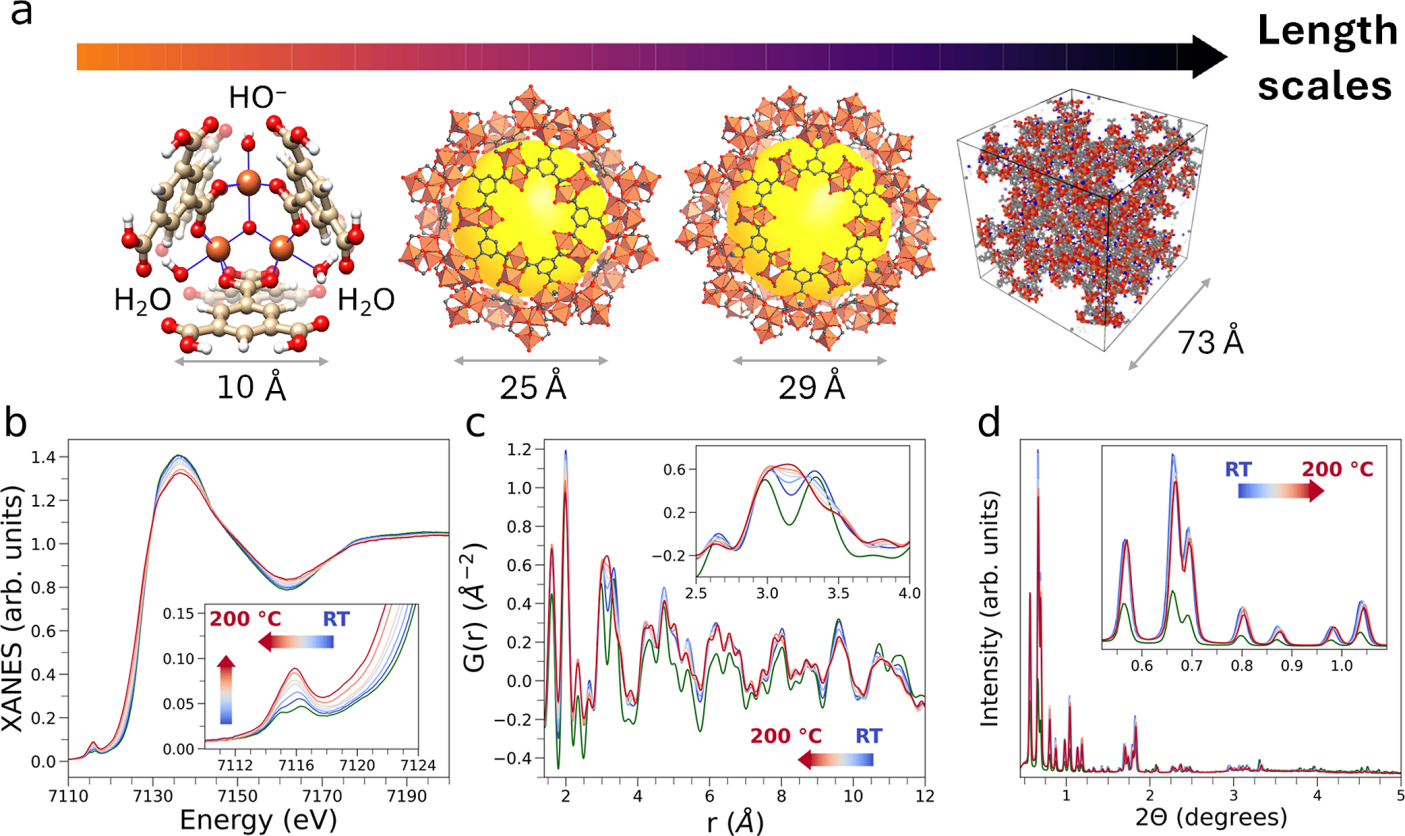
(a) Overview of the MIL-100­(Fe) crystal structure at increasing length scales. From left to right, the figure highlights the MOF trimeric unit, the two distinct MOF pore environments featuring pentagonally- and hexagonally shaped pore windows, and the MOF unit cell, respectively. Color code: iron, orange; carbon, light gold; oxygen, red; hydrogen, white. (b–d) Sequence of Fe K-edge XAS spectra (b), real-space X-ray PDF patterns (c), and PXRD patterns (d) collected on the MIL-100­(Fe) pristine sample (green) and during sample thermal activation in He flux (blue, 25 °C; red, 200 °C for 2 h). Arrows are drawn to highlight the evolution of selected features in the XAS, PDF and PXRD data.

### Thermal Activation of MIL-100­(Fe)

In the first step of our study, we investigated the nature of the structural and electronic modifications occurring during the thermal activation of MIL-100­(Fe) by employing complementary synchrotron X-ray techniques. To this end, we heated a MIL-100­(Fe) sample from room temperature (RT) to 200 °C in a pure He flux to remove physisorbed impurities and monitored the MOF thermal treatment by performing simultaneous Fe K-edge XAS, X-ray PDF and PXRD measurements. Figure 1b compares the Fe K-edge XAS spectrum of the pristine MIL-100­(Fe) sample collected in ambient air to those collected from RT to 200 °C in flowing He for 2 h. The X-ray absorption near edge structure (XANES) of pristine MIL-100­(Fe) exhibits two transitions in the pre-edge region located at 7115.0 and 7116.4 eV (inset of Figure 1b). These features are known to be due to 1s → 3d transitions into the e_g_ and t_2g_ orbitals^48,49^ and are directly related to the octahedral coordination environment of the iron sites in the pristine MOF. After exposing the MOF to a He flux and gradually increasing the temperature up to 200 °C, the two pre-edge features eventually coalesce into a single broad peak centered at 7115.9 eV (inset of Figure 1b), due to the formation of iron sites coordinated in a square pyramidal-like geometry.^48,50^ It is in fact known that, upon thermal treatment, the Fe-bound water molecules are first progressively removed from the triiron units, and that at higher temperatures a portion of the Fe-coordinated hydroxyl groups are released leading to MOF trimeric units with coordinatively unsaturated iron centers, a fraction of which are Fe­(II) sites depending on the duration and temperature of the activation treatment.^48,50^ The reduction of a fraction of the iron atoms to Fe­(II) is proved by the shift of the edge position of the XANES spectra from approximately 7124.6 to 7123.3 eV during the thermal activation process. Since the thermal activation and dehydration procedures were carried out at temperatures lower than or equal to 200 °C and most of the reduction processes occur above 225 °C, we assume herein that in our experimental conditions the amount of Fe­(II) is negligible below 150 °C and lower than ∼5% at higher temperatures, as estimated in a recent XAS study of MIL-100­(Fe).^48^

The X-ray PDF of pristine MIL-100­(Fe) collected in ambient air (Figures 1c and S3) displays structural correlations above >20 Å that are consistent with the crystalline nature of the MOF, as well as the previously reported short-range peaks at 1.98, 2.98, 3.34, and 4.68 Å, which have been attributed to Fe–O, Fe–C, Fe–Fe, Fe–O and C–O correlations.^44^ As a result of thermal treatment, the peaks at 2.98 and 3.34 Å gradually coalesce in a single peak (inset of Figure 1c) due to a decrease of the average Fe–Fe distance from 3.34 to 3.16 Å and to a slight increase of the Fe–C distance from 2.98 to 3.02 Å, in agreement with previous PDF measurements of thermally treated MIL-100­(Fe).^51^ These effects may be attributed to modifications in the MOF local structure resulting from the temperature-induced removal of guest molecules adsorbed in the MOF pores. Further, the relative intensity of the Fe–O peak at 1.98 Å is found to decrease due to the reduction in the average Fe coordination number.^51^ Structureless Le Bail refinements of the PXRD data (Figure 1d) confirmed the phase purity of the MIL-100­(Fe) sample (Figures S4–S9) in the explored temperature range and evidenced a slight contraction of the unit cell (from 73.080(1) Å at RT in air to 72.529(2) Å at 200 °C in He) due to the removal of species physisorbed within the MOF pores.^48^ Interestingly, the PXRD pattern collected while exposing the MOF to a He flux at RT (light blue curve, Figure 1d) exhibits a higher relative intensity as compared to that of the PXRD pattern of the pristine MOF measured at RT in ambient air (green curve, Figure 1d). We attribute this effect to the desorption of disordered guest molecules adsorbed in the MOF pores by He and to the contextual enhancement of the diffracted intensity resulting from the ordered MOF framework. This effect is particularly significant for the lower angle peaks that are associated with crystal planes of high d-spacings, as previously observed in a study targeting the adsorption of Xe in zeolite materials.^52^ Altogether, the XAS, PDF and PXRD measurements clearly indicate that in the activated MOF open iron sites are present while the long-range order is preserved.^48,51^

### Investigation of MOF Water Harvesting and Thermal Dehydration

To characterize the structural and electronic properties of MIL-100­(Fe) during atmospheric water harvesting across short- and long-range length scales, we then employed in situ XAS, X-ray PDF and PXRD measurements to monitor the MOF hydration and subsequent thermal dehydration in real time. Due to experimental limitations, we were unable to achieve partial water loadings on MIL-100­(Fe) during the XAS/PDF/PXRD measurements. These were indirectly explored by progressively dehydrating the MOF with thermal heating after fully hydrating the framework. To this end, we cooled the previously activated MIL-100­(Fe) sample down to RT and exposed it to a flux of He saturated with water in order to fully hydrate the MOF (the hydration protocol is detailed in Section S1.3 of the Supporting Information). We then followed the thermal dehydration of the hydrated MIL-100­(Fe) sample by applying discrete temperature increments from RT to 200 °C while keeping the sample under a flux of anhydrous He. The evolution of the Fe K-edge XAS spectra recorded during the thermal dehydration of the MOF is shown in Figure 2a. The XAS spectrum of the hydrated MOF exhibits the two transitions at 7115.0 and 7116.4 eV in the pre-edge region which, as discussed above, are related to the presence of octahedral Fe sites (inset of Figure 2a). Conversely, the pre-edge region of the XAS spectrum of the dehydrated MOF at 200 °C displays a single peak at 7115.9 eV assigned to the presence of open, square-pyramidal iron sites (red curve, Figure 2a). Furthermore, while dehydrating the MOF there is a shift of the main XANES edge to lower energy and a decrease in the intensity of the XANES white-line transition at about 7136 eV (Figure 2a). These spectral variations may be attributed to the temperature-dependent release of water molecules and of a minor fraction of hydroxyl ligands, in a practically equivalent manner as that observed during the initial thermal activation. The magnitude of the Fourier Transform (FT) of the extended X-ray absorption fine structure (EXAFS) spectra calculated in the 1.8–9.0 Å^–1^ *k* range is shown in Figure 2b. As the temperature increases, the intensities of the first and second peaks of the FT’s diminish. This is due to the decrease in the number of oxygen-based ligands bound to the iron sites during dehydration as well as to the increase of the Debye–Waller factor associated with thermal and structural disorder. This observation is fully consistent with the evolution of the XANES region in the spectra and may also be attributed to the progressive removal of water and hydroxyl ligands coordinated to the iron sites in the trimeric units of hydrated MIL-100­(Fe).

**Figure 2:**
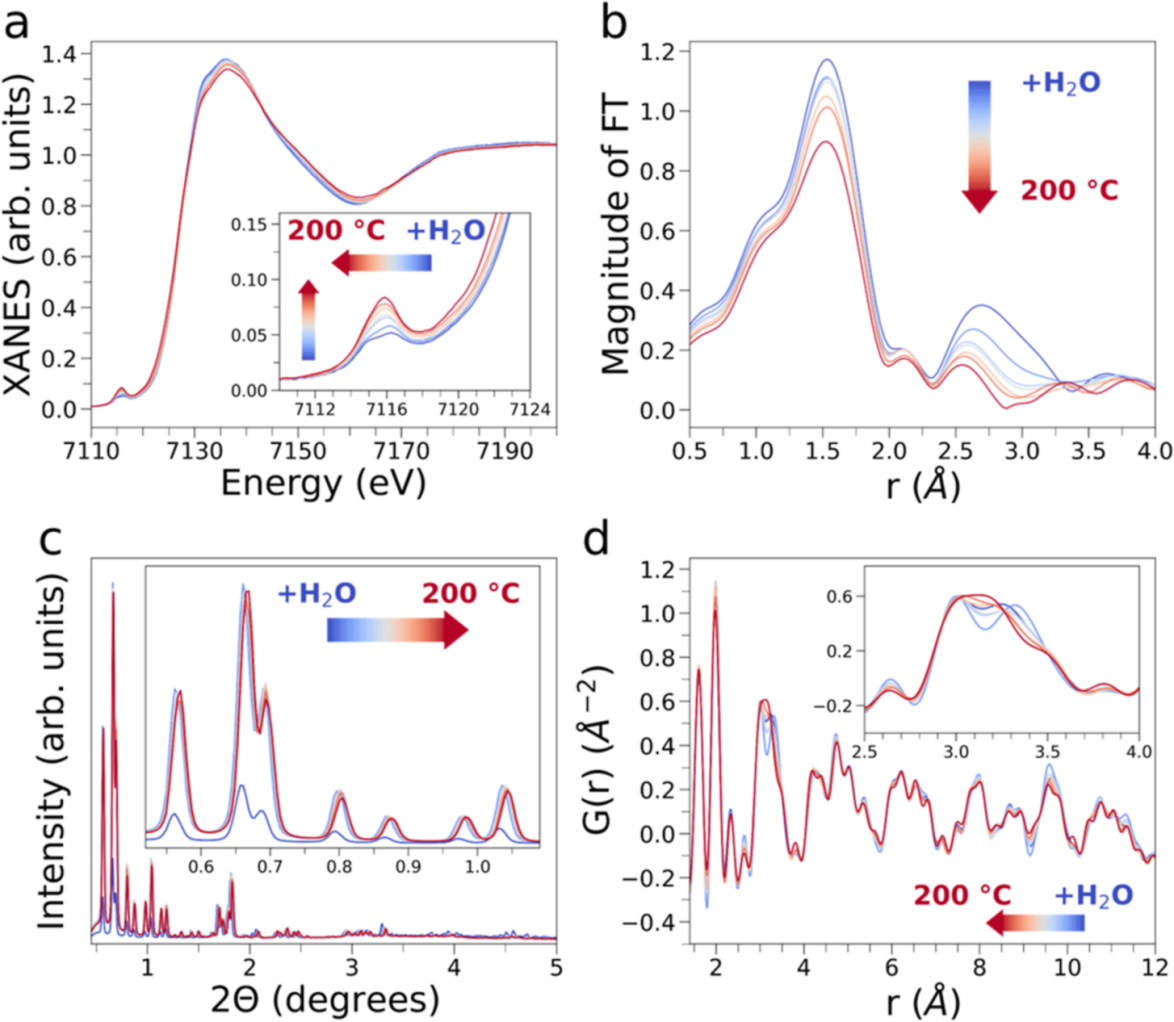
Thermal dehydration of MIL-100­(Fe) monitored between RT and 200 °C by complementary synchrotron X-ray techniques. Sequence of (a) Fe K-edge XAS spectra, (b) nonphase shift corrected FT magnitudes of the EXAFS signal, (c) PXRD patterns and (d) real-space X-ray PDF patterns simultaneously collected on the hydrated MIL-100­(Fe) sample (dark blue) and during sample thermal dehydration in He flux (light blue, 25 °C; dark red, 200 °C). Arrows are drawn to highlight the evolution of selected features in the XAS, FT, PXRD and PDF data.

Upon hydration, the unit cell parameter expands from 72.785(2) to 73.448(1) Å and then progressively decreases up to 72.523(2) Å during thermal dehydration, as evidenced by the structural parameters obtained in the structureless Le Bail refinements reported in Table S1 (see Figures S11–S16). A low intensity is observed in the PXRD patterns of the hydrated sample (dark blue curve, Figure 2c) and a subsequent increase is detected upon dehydration (light blue to dark red curves, Figure 2c) due to the uptake and release of water from the MOF pores, respectively, as previously discussed. The PDF of hydrated MIL-100­(Fe) (dark blue curve, Figure 2d) presents all of the long- and short-range correlations described for the PDF of the pristine MOF (see Figure 1c for comparison). During the thermal dehydration the PDFs display variations similar to those measured during the sample thermal pretreatment. In particular, the double peak present in the region between 2.9 and 3.5 Å, that is associated with the Fe–Fe contribution, becomes a single peak confirming that when the thermally activated MOF is exposed to wet He a slight and reversible elongation of the trimeric nodes occurs.^51^ Moreover, the intensity of the peak at 1.98 Å decreases, due to the release of water and of, likely, a small fraction of hydroxyl groups from the MOF. Importantly, the complementary EXAFS and PDF measurements allow to disentangle how the first- and second-shell structural properties of the MOF Fe sites evolve. The decrease in intensity of the first peak at 1.5 Å in the FT transform and of the peak at 1.98 Å in the PDF are due to the progressive departure of the water molecules directly bound to the iron centers. Further, the removal of water molecules located beyond the first coordination shell of the metal sites leads to an intensity decay and a shift toward lower distances of the peak at about 2.7 Å in the FT spectrum.

### MD Simulations of the Water Harvesting Process

In order to obtain local and global insights into the mechanism of the MOF water uptake, we performed a computational analysis of the structural and dynamical properties of water confined within MIL-100­(Fe). In particular, we employed classical MD simulations to describe the behavior of water by means of the TIP4P-Ew water force field^32^ together with a recently proposed density functional theory (DFT)-derived interaction model, a combination that was found able to correctly reproduce the water adsorption isotherm of MIL-100­(Fe).^31^ MD simulations were performed at water loadings in the *N* = 2 to *N* = 40 range (additional details on the MD simulations are provided in Table S2) to study the full hydration of the iron local environment, where N is the number of water molecules per MOF trimeric unit. The selected water loadings were chosen in an effort to reproduce the weight percent water uptake by MIL-100­(Fe) previously determined through water adsorption isotherm measurements.^31^ The Fe–O radial distribution functions (RDFs) between the iron atom and the oxygen atoms of the water molecules for all of the explored water loadings (Figure S17) show the presence of a first peak with a maximum at about 2.1 Å (Table S3), in good agreement with previous computational work.^31^ In all cases one water molecule coordinates the iron atom in the apical position, while a second coordination sphere of water molecules is found at increasing water content at a distance of about 3.85 Å.

To shed light on the pore filling mechanism in MIL-100­(Fe), Figure 3 displays representative MD snapshots of MIL-100­(Fe) loaded with *N* = 2, 10, 20, 30, and 40 water molecules per trimeric unit, together with the density maps of water calculated from the related MD simulations. At a loading of *N* = 2, the water molecules confined in the MOF are localized near two of the three iron sites in the trimeric units, following a structural arrangement similar to that displayed in Figure 1a. As the water content increases, the Fe-bound water molecules and the hydroxyl ligands play a key role in initiating water uptake. To elucidate how water interacts with the MOF hydroxyl ligands, we have calculated the percentage of hydroxyl ligands that donate a hydrogen bond to water (Figure S18 and Section S1.4 for additional details on the analysis). The number of hydroxyl groups involved in a hydrogen bond with water steadily increases as a function of water content suggesting that this interaction is an important step during the initial water harvesting stage. Further, the evolution of the water density maps (Figure 3) indicates that the MOF water uptake process is spatially dependent, with certain unit cell regions filling before others owing to the two distinct pore environments present in MIL-100­(Fe), which are highlighted in Figure S19. By looking at Figure 3, one may note that for *N* = 10 water is more concentrated in the MOF pores with reduced diameter while for *N* = 20 these cavities have been fully populated, with the larger pores of MIL-100­(Fe) being still mostly empty. Further, when *N* = 30 the water molecules have partially filled the MOF pores with larger diameter, while for *N* = 40 also the larger pores have been effectively occupied by water. Representative MD snapshots and calculated density maps of water for all investigated water loadings are displayed in Figure S20 and fully support these trends. Overall, the MD analysis indicates that as larger amounts of water are adsorbed within the MOF, water molecules first populate the MOF pores with pentagonally shaped windows before filling the pores with hexagonally shaped windows, as expected from the double step observed in the experimental water adsorption isotherm of MIL-100­(Fe) and in agreement with previously reported MD calculations.^31^ Importantly, the MD simulations indicate that the observed pore filling order arises mostly from thermodynamic factors and is not limited by kinetic factors. This finding is in line with the small van der Waals radius of H_2_O with respect to the small- and large-pore window apertures of MIL-100­(Fe) (∼9 and ∼5 Å, respectively).^26^

**Figure 3:**
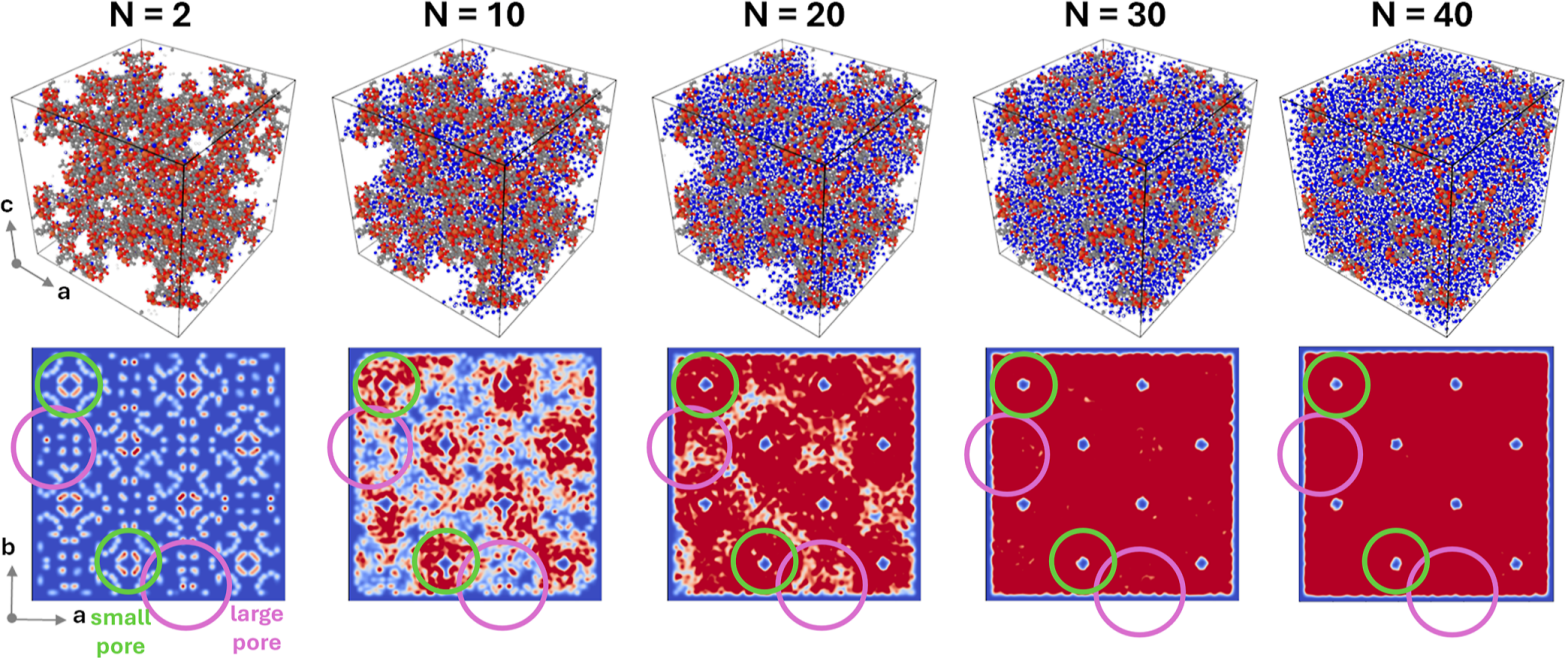
Side views of MD snapshots of MIL-100­(Fe) loaded with *N* = 2, 10, 20, 30, and 40 water molecules per trimeric unit (top panels). The iron, carbon, oxygen and hydrogen atoms of the MOF are show in orange, gray, red, and white, respectively. The oxygen and hydrogen atoms of the water molecules are displayed in blue and white, respectively. Density maps of water (bottom panels) calculated from MD simulations of MIL-100­(Fe) loaded with *N* = 2, 10, 20, 30, and 40 water molecules per trimeric unit (darker red colors are used to depict regions with higher water density). Representative regions of space occupied by large and small pores are highlighted by purple and green circles, respectively.

To elucidate the evolution of hydrogen bonding among water molecules within MIL-100­(Fe) as a function of N, Figure 4a shows the distribution of different hydrogen-bonding topologies at increasing water loadings. Here, each water molecule has been classified in terms of the average number of hydrogen bonds either donated (HBD) or accepted (HBA). In particular, two water molecules were defined as hydrogen bonded if they were located within a distance *R*_D–A_ ≤ 3.0 Å between donor (D) and acceptor (A) oxygen atoms while forming an angle θ_DHA_ ≥ 150°.^23,25^ At a loading of *N* = 3, most (∼42%) water molecules do not participate in hydrogen bonds (0HBD–0HBA), while a minor fraction (∼22%) donates and accepts a single hydrogen bond (1HBD–1HBA). On the other hand, at a water loading of *N* = 4 the 0HBD–0HBA fraction of water molecules decreases to about 24% while the 1HBD–1HBA fraction becomes dominant (32%) reaching a maximum value of ∼37% for *N* = 6. For loadings of 6 < *N* ≤ 16 the following trends are observed: (i) the 0HBD–0HBA and 1HBD–1HBA values progressively decrease below 2% and 22%, respectively, (ii) the fraction of water molecules donating two hydrogen bonds and accepting one hydrogen bond (2HBD–1HBA) increases up to 20%, (iii) the relative portion of water molecules donating one hydrogen bond and accepting two hydrogen bonds (1HBD–2HBA) and the number of water molecules donating and accepting two hydrogen bonds (2HBD–2HBA) reach ∼20% and ∼19%, respectively. Once the water content reaches *N* = 18, the relative abundance of water molecules donating and accepting two hydrogen bonds (i.e., those behaving like liquid-phase water) becomes predominant, and gradually increases up to ∼45% for *N* = 40. In addition, the increase of the 2HBD–2HBA water fraction also appears to occur at the expense of the 2HBD–1HBA and 1HBD–2HBA fractions of water molecules which gradually begin to decrease for *N* > 24 and *N* > 26, respectively.

**Figure 4:**
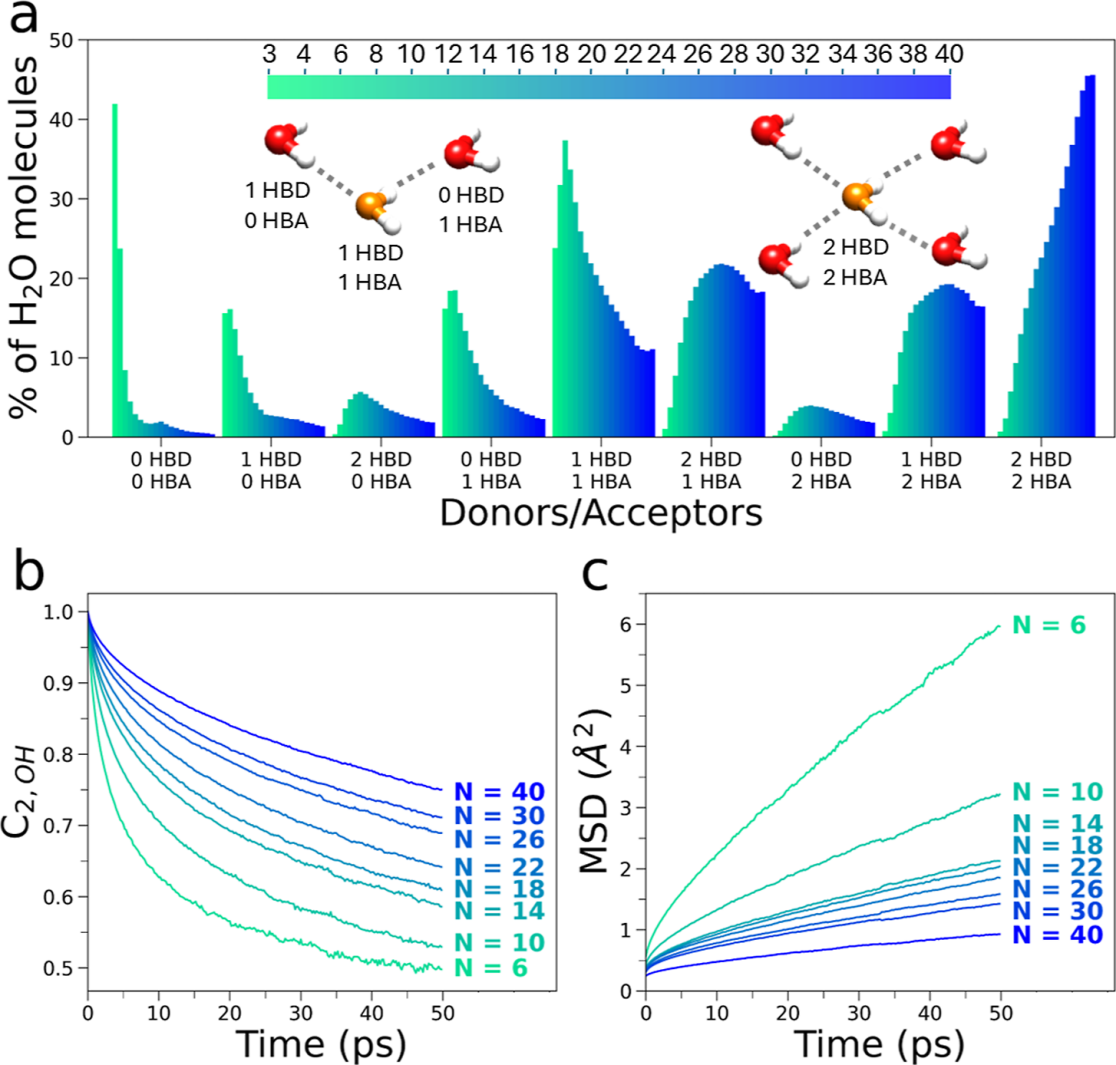
(a) Evolution of hydrogen-bonding topologies of water molecules confined within MIL-100­(Fe) calculated from MD simulations carried out at increasing water content (the considered water loadings are listed above with the color bar). The oxygen atoms of water are displayed in red and orange, while hydrogen atoms are displayed in white. Lighter green and darker blue colors represent the distribution of hydrogen bond topologies calculated for lower and higher water loadings, respectively. The number of hydrogen bond donors and acceptors is expressed as a percentage of the total number of water molecules in MIL-100­(Fe). Representative clusters of water molecules with different hydrogen-bonding topologies are shown. (b,c) Water orientational correlation functions (b) and water mean squared displacement (c) obtained from MD simulations of MIL-100­(Fe) at increasing water loadings.

To obtain insights into the dynamics of the water hydrogen-bonding network, we calculated the hydrogen bond time autocorrelation function^53^ at increasing water loadings (further details are provided in Section S1.4 of the Supporting Information). Consistently, we found that the time autocorrelation of the water–water hydrogen bonds decays more slowly as a function of water content (Figure S21), indicating that the underlying interactions are strengthened as a more effective hydrogen bond network is established.

Further insights into the dynamical behavior of water were obtained from the MD simulations by calculating the orientational correlation function of the O–H bond, C_2,OH_, as a function of N (Figures 4b and S22). In particular, the orientational correlation function was evaluated as

1 $$C_{2,OH}=\left\langle P_{2}\left[û\left(t=0\right)\cdot û\left(t\right)\right]\right\rangle$$

where $P_{2}\left[û\left(t=0\right)\cdot û\left(t\right)\right]$ is the second-order Legendre polynomial of the angle formed by the OH unit vector of a specific H_2_O molecule at a certain time *t* and the unit vector of the same molecule at *t* = 0, while the brackets indicate an ensemble average over all water molecules.^21^ Altogether, we found that the reorientational mobility of water molecules adsorbed within the MOF slows down as the increase of water content leads to a more effective hydrogen-bond network that hinders the reorientation ability of water. Indeed, Figure S22 shows that the Fe-bound water molecules reorient faster when alone (*N* = 2) if compared to the water molecules engaged for the most part in hydrogen-bonding clusters (*N* = 6). Further, the reorientation rate of the water molecules progressively slows down as a function of *N* (Figure 4b), a result consistent with computational studies of water within other MOF systems.^21,24^ On the other hand, the translational mobility of water adsorbed in MIL-100­(Fe) decreases as a function of water loading. In fact, the mean squared displacement (MSD) of water calculated for 6 ≤ *N* ≤ 40 decreases as a function of *N*, as shown in Figure 4c (see Section S1.4 of the Supporting Information for additional details on the MSD analysis). This dynamical behavior may be attributed to the strengthening of the hydrogen-bond network as a function of N, which in turn limits the orientational and translational ability of water.

### Theoretical XAS Analysis of the Water Harvesting Process

An in-depth analysis of the Fe K-edge XAS spectra was then carried out by combining the description of the water uptake process obtained from the MD simulations together with ab initio XAS simulations. To this end, Fe K-edge XAS theoretical average spectra were calculated from 20 snapshots of the MD simulations carried out for all investigated water loadings in the *N* = 2 to *N* = 40 range (details on the XAS calculations are provided in Section S1.5 of the Supporting Information). In particular, the theoretical XAS calculations have been performed over 94 distinct iron trimeric units for each MD simulation (Figure S23) to minimize the role of slight differences in the node structures due to the complexity of the structural model. The convergence of the theoretical average spectra as a function of the number of MD snapshots employed in the calculations was also thoroughly monitored. The theoretical average XAS spectra calculated from 100 snapshots of the MD simulations and over 3 selected iron trimeric units are shown in Figures S24–S44, highlighting the spectral contribution of each individual iron site. Further, Figures S45–S47 show that the theoretical average spectra only display minimal differences when evaluated for a number of MD snapshots greater than 20, indicating that in all cases the XAS theoretical curves are well-converged when 20 MD snapshots are averaged.

The nature of the interactions established between the water molecules and the MOF framework were further dissected by analyzing how increasing the water content contributes to the theoretical Fe K-edge XAS spectra. Figure 5a briefly illustrates the evolution of the Fe coordination environment when the open Fe­(III) sites interact with one or more water molecules. A comparison of the experimental XANES spectra collected for the hydrated and dehydrated MIL-100­(Fe) sample is shown in Figure 5b, while the corresponding theoretical spectra calculated from the MD simulation of the fully hydrated MOF (*N* = 40) and from the dehydrated MIL-100­(Fe) unit cell with solely hydroxylated trimeric units are shown in Figure 5c. One may note that the single peak in the pre-edge region, which is ascribed to the presence of Fe centers coordinated in a square-pyramidal configuration, is correctly reproduced in the calculation. Notably, the full removal of the hydroxyl ligands coordinating the Fe sites afforded theoretical XAS spectra exhibiting a less intense white–line transition, a shift toward lower energies of the main XANES absorption edge, and also a more intense pre-edge feature (Figure S48). These theoretical results are in good agreement with the previously discussed formation of open Fe­(II) sites in MIL-100­(Fe) upon thermal treatment above 225 °C, which is known to lead to a less intense white-line transition in the XAS spectra, as well as to a shift of the main absorption edge toward lower energies and a more intense pre-edge peak.^48,50^ Further, the theoretical XAS spectrum of the fully loaded MOF (Figure 5c) correctly reproduces the relative energy positions and intensities of the two features in the pre-edge region of the XAS experimental spectrum of hydrated MIL-100­(Fe) (Figure 5b). The simulated XAS spectra also closely reproduce the energy shift and relative intensity of the white-line transition in the experimental XAS spectra of the hydrated and activated MOF. Overall, the excellent agreement between theoretical and experimental XAS data supports the validity of our computational model.

**Figure 5:**
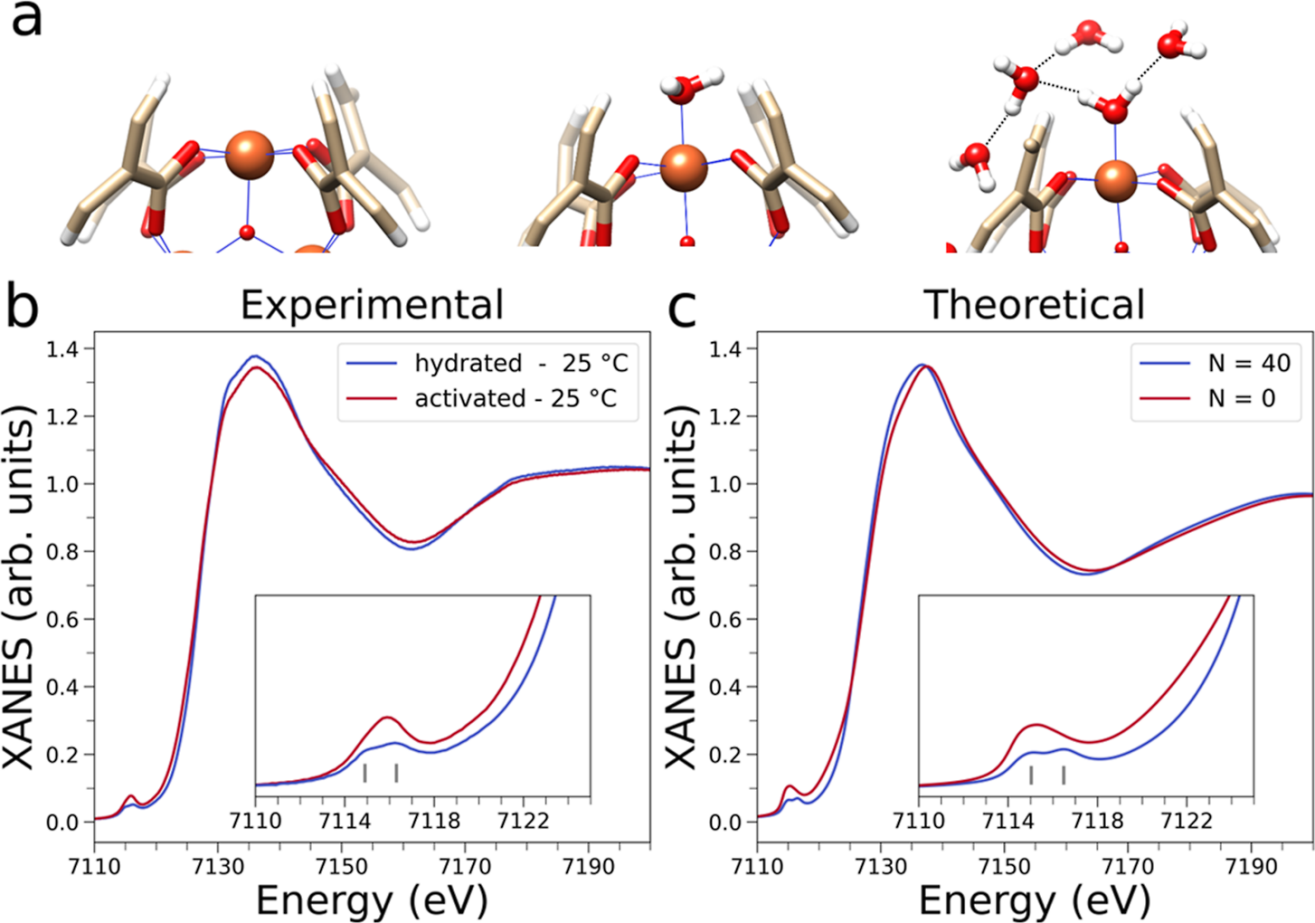
(a) Evolution of the Fe site environment with increasing water loadings in the MD simulations: open metal site (left), water-coordinated Fe site (middle) and hydrogen-bonded cluster (right). Iron, oxygen, carbon and hydrogen atoms are depicted in orange, red, light yellow and white, respectively. (b) Experimental Fe K-edge XAS spectra of hydrated MIL-100­(Fe) (blue curve) and of the thermally activated MOF (red curve) collected at 25 °C. In the inset, vertical gray lines highlight the two transitions of the pre-edge region. (c) Theoretical Fe K-edge XAS spectra. The blue curve is calculated from MD simulations of MIL-100­(Fe) loaded with *N* = 40 water molecules per trimeric unit, while the red curve is calculated starting from the dehydrated MOF structure. In the inset, vertical gray lines highlight the two transitions of the pre-edge region.

To gain statistical insights into the relationship between the water harvesting mechanism of MIL-100­(Fe) and the local structure encoded by the theoretical average XAS spectra calculated as a function of N, Figure 6 shows the results of a uniform manifold approximation and projection (UMAP)^54^ analysis of the simulated XAS data. UMAP is a relatively recent algorithm for dimensionality reduction that has been found able to overcome limitations, including representation overcrowding and loss of large-scale information, that affect more common data reduction techniques such as principal component analysis (PCA) and *t*-distributed stochastic neighborhood embedding (*t*-SNE).^55,56^ UMAP visually revealed that the theoretical XAS average spectra cluster separately as a function of N and in close connection to the prevalent hydrogen-bonding topology of water at the given water loading. In particular, the theoretical XAS spectra calculated for *N* = 2 and *N* = 3, corresponding to MD configurations where the 0HBD–0HBA fraction of water molecules is prevalent, occupy very close regions in the UMAP space. At a water loading of *N* = 2, the direct binding of water molecules to the MOF open iron sites yields a variation in the theoretical XANES signal, most notably with the appearance of two distinct features in the pre-edge region which are sensitive fingerprints of the coordination geometry change from square pyramidal to octahedral (Figures S49 and S50).

**Figure 6:**
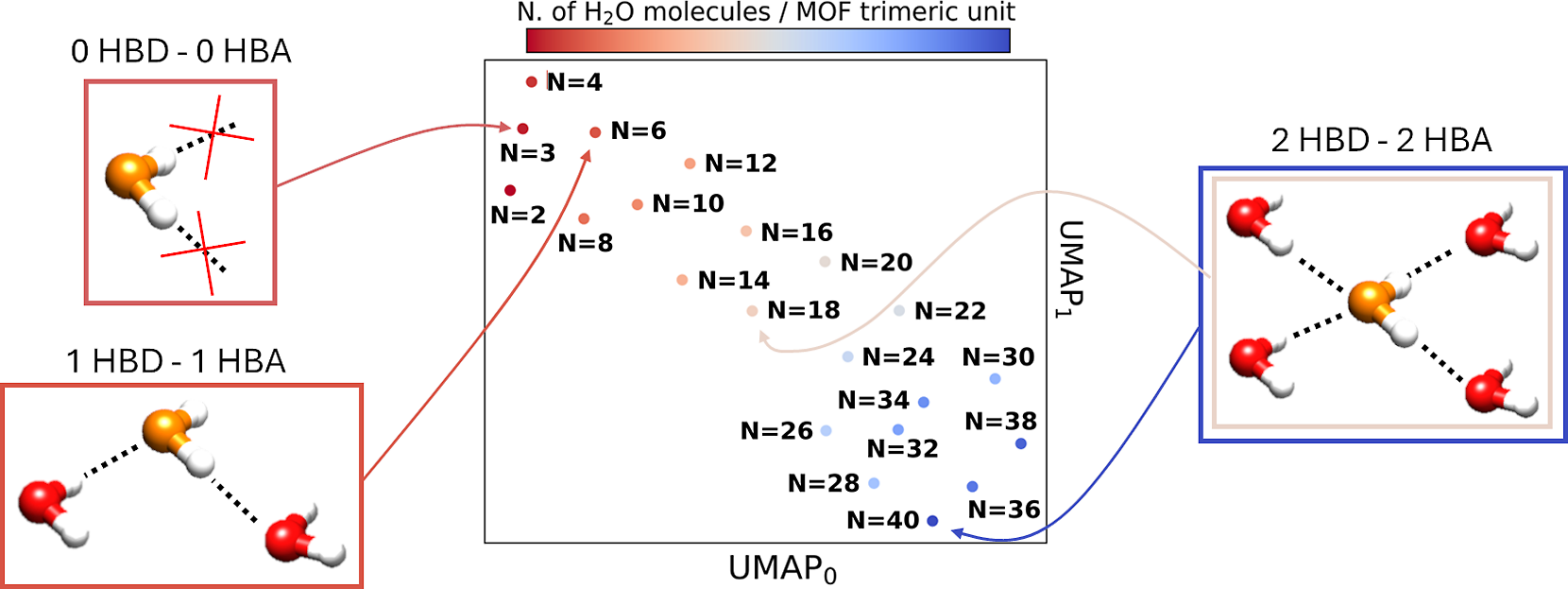
UMAP representation of the theoretical Fe K-edge XAS average spectra calculated as a function of N. Representative clusters of water molecules exhibiting the hydrogen-bonding topology prevalent at the given water loading are shown.

These first-shell water molecules directly interact with the iron sites at a distance of about 2.1 Å, do not diffuse away from the iron centers and exhibit a high reorientational freedom. As the water loading is increased, the evolution of the topological properties of the water hydrogen-bond network is closely reflected by consistent changes in the theoretical XAS spectra. In particular, at a water loading of *N* = 3 there is a slight decrease in intensity of the first XANES pre-edge transition at 7115.0 eV if compared to that of the XANES calculated for *N* = 2 (Figure S50). This first spectral variation may be ascribed to the change in local structure around the iron sites stemming from the formation of hydrogen bonds at a water loading of *N* = 3, with 22% water molecules donating and accepting one hydrogen bond (Figure 7a). At a water loading of *N* = 6 the hydrogen-bond topology significantly changes, with the great majority of water molecules now donating and accepting a single hydrogen bond (Figure 7c). This topology variation is accompanied by a second slight change in the associated theoretical XAS signal. As shown in Figure 7d, the XAS spectrum simulated for *N* = 6 exhibits a decrease in intensity of the first pre-edge feature if compared to that of the XAS spectrum calculated for *N* = 3. For water loadings in the 4 ≤ *N* ≤ 16 range, the majority of water molecules donate and accept a single hydrogen bond. Accordingly, for 4 ≤ *N* ≤ 16 the related theoretical XAS averages progressively cluster in a different region of the UMAP space (Figure 6) and exhibit intensities of the first XANES pre-edge transition at 7115.0 eV that smoothly decrease and become very similar to those of the feature at 7116.4 eV (Figure S50). Also in this case the UMAP clustering behavior appears to be connected to the variation in hydrogen-bonding topology, as for these water loadings the 1HBD–1HBA fraction of water molecules remains the greatest. As the amount of water is further increased, the hydrogen bond topological properties change again, with the fraction of water molecules donating and accepting two hydrogen bonds becoming predominant for *N* = 18 (Figure 7e). This ulterior hydrogen-bond topology variation is followed by a visible intensity decrease of the low-energy feature in the pre-edge of the XANES spectrum calculated for *N* = 18 (Figure 7f), which is less intense than that of the XAS spectrum simulated for *N* = 6 (Figure 7d). This more evident spectral variation may be explained as due to the increase of the 2HBD–2HBA, 1HBD–2HBA and 2HBD–1HBA fractions of water molecules and to the decrease of the 1HBD–1HBA fraction (Figure 7e). Finally, when increasing the water loadings up to *N* = 40 there is a further small decrease in intensity of the low-energy pre-edge feature (Figure 7h) that appears to be linked to the further increase of the 2HBD–2HBA fraction up to ∼45% (Figure 7g). This structural–spectral relationship is further supported by the UMAP analysis of the XANES data (Figure 6), which indicates that for *N* ≥ 18 the associated XAS spectra cluster in close vicinity.

**Figure 7:**
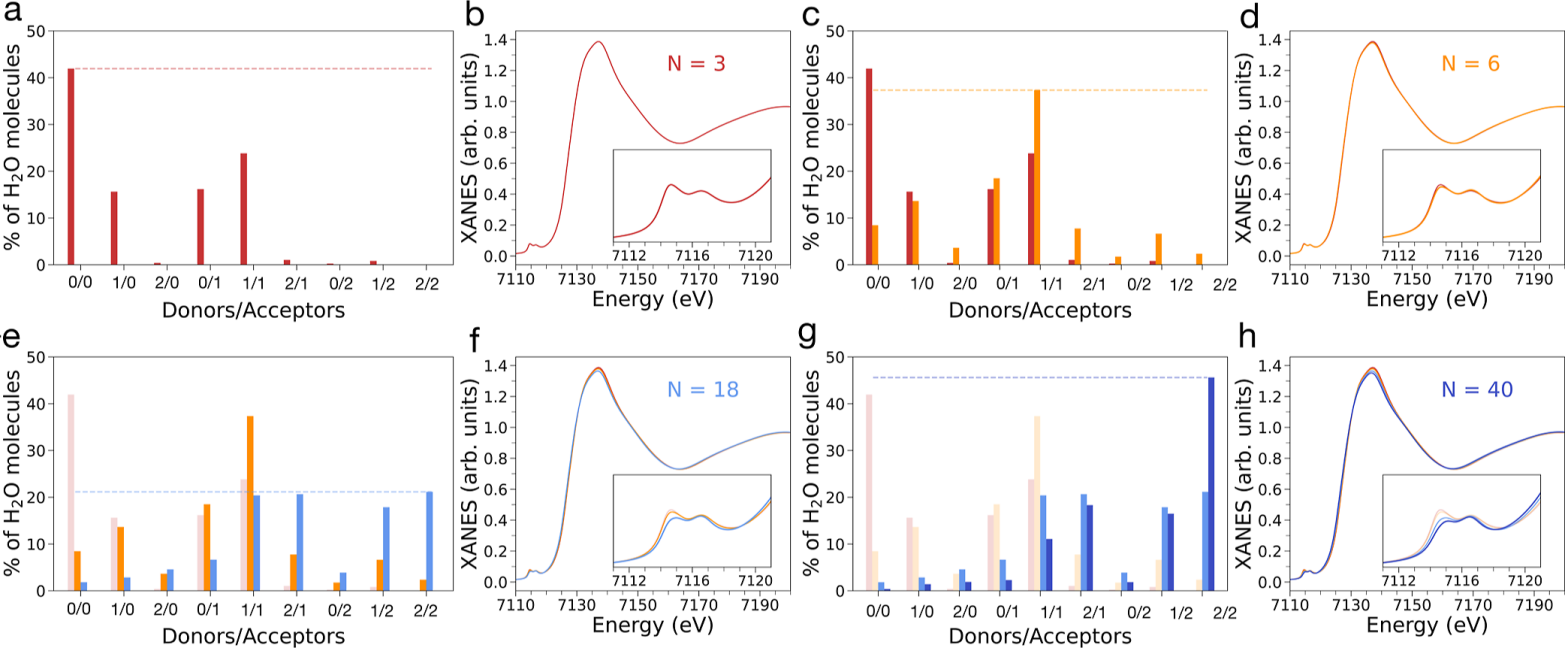
Hydrogen-bonding topologies of water molecules (panels a, c, e, g) confined within MIL-100­(Fe) and corresponding theoretical Fe K-edge XAS spectra (panels b, d, f, h) calculated from MD simulations carried out at water loadings of *N* = 3, 6, 18 and 40, respectively.

These theoretical findings evidence the sensitivity of XAS to rearrangements of the hydrogen-bond network of water confined within MIL-100­(Fe), which influence the local structural and electronic properties of the Fe sites and are therefore promptly probed by the variations of the XANES signal.

## Conclusions

In this work, we employed a new approach based on Fe K-edge XAS, PXRD and X-ray PDF in combination with MD simulations and ab initio theoretical XAS calculations to gain a comprehensive picture of the water harvesting process by MIL-100­(Fe), moving progressively from the local to global length scale. The applied strategy, making use of complementary synchrotron X-ray techniques to probe the evolution of the structural and electronic properties of MIL-100­(Fe) in real time across short-, intermediate- and long-range distances, was able to overcome the experimental challenges associated with collecting quantitative local structural information during MOF water harvesting. In particular, the complementary EXAFS and PDF measurements allowed us to shed light on how the first- and second-shell structural properties of the MOF Fe sites evolve. We exploited experimental XAS data to benchmark MD simulations, which in turn allowed us to identify the sequence of MOF pore filling, the key MOF structural modifications during the distinct water uptake stages, the evolution of the dynamical properties of water as well as the modifications of the topological properties of the water hydrogen-bond network, as a function of water content. Altogether, our experimental and theoretical approach revealed the Fe-bound water and hydroxyl moieties as key initiators of MOF pore filling processes, with subsequently adsorbed water molecules clustering first in the smaller mesopores before filling the larger mesopores. The water molecules exhibited increasingly reduced mobility and reorientational ability, due to the establishment of a progressively denser hydrogen bond network with higher water content. Notably, the XAS technique was proven to be sensitive not only to the first- and second-shell structural modifications induced by increasing water content, but also to the evolution of the hydrogen-bond network of water adsorbed in the structurally complex MIL-100­(Fe). This work not only represents an innovative approach to investigate porous materials for water harvesting applications, but also holds significant potential to highlight often elusive properties of water confined in MOFs, paving the way for a more widespread integration of X-ray synchrotron methods in the reticular chemistry innovation cycle.

## Supplementary Material



## Data Availability

All data presented in this study are available from the corresponding authors upon reasonable request.

## Notes

Supporting Information synthesis of MIL-100­(Fe), XAS/PXRD experimental details and data treatment, MD simulation analysis details, Figures S1–S52, Tables S1–S4 (PDF)
