# Supplementary material for: Decoding the Water Harvesting Mechanism of MIL-100(Fe) Across Short- and Long-Range Length Scales
Source: J Am Chem Soc. 2025 Oct 28;147(44):40507–18. doi: 10.1021/jacs.5c12269 (PMC12593329; doi:10.1021/jacs.5c12269)
Supplement: Supplementary file 1 [file ja5c12269_si_001.pdf]

Supporting Information: Decoding the water harvesting mechanism of MIL-100(Fe) across short- and long-range length scales

Francesco Tavani,*,† Alessandro Tofoni,*,† Eva Pietropaoli,† Dragos Costantin Stoian,‡ Wouter van Beek,‡ Kenneth Marshall,‡ Ida Pettiti,† Alessandro Latini,† and Paola D'Angelo*,†

†*Dipartimento di Chimica, Università degli Studi di Roma "La Sapienza", P.le A. Moro 5, I-00185 Rome, Italy*

‡*Swiss–Norwegian Beamlines, European Synchrotron Radiation Facility, 71 Avenue des Martyrs, 38000 Grenoble, France*

E-mail: francesco.tavani@uniroma1.it; alessandro.tofoni@uniroma1.it; p.dangelo@uniroma1.it

Contents

1 Experimental Methods **S-2**

1.1 Synthesis of MIL-100(Fe) S-2

1.2 N_2 Adsorption Measurements S-3

1.3 Details on the *in situ* XAS/PXRD Experiments and Data Treatment S-3

1.4 Analysis of the structural and dynamical properties of water in the MD simulations S-4

1.5 *Ab Initio* XAS Calculations S-6

2 Supplementary Figures (Figures S1–S52) S-8

3 Supplementary Tables (Tables S1–S4) S-53

References S-56

1 Experimental Methods

1.1 Synthesis of MIL-100(Fe)

All reagents were purchased from Sigma Aldrich and employed as received. In the first step of the synthetic procedure, ferrous sulfate heptahydrate (3.168 g, 11.4 mmol) was dissolved in 98 cm^3 of distilled water, while trimesic acid (1.600 g, 7.6 mmol) was dissolved in 23 cm^3 of an aqueous 1 M NaOH solution while applying magnetic stirring. Subsequently, the solution of trimesic acid was added dropwise into the green solution of $FeSO_4 \cdot 7H_2O$ under magnetic stirring. The stirring was maintained for 40 h at room temperature (RT). The orange-brown solid product was collected by centrifugation and washed sequentially with deionized water for 3 times and ethanol for 3 times in order to remove the unreacted species. Between each washing step the MOF product was immersed in approximately 15 mL of water or ethanol for 45 minutes, mixed using a vortex mixer for 1 minute, and ultrasonically treated for 3 minutes. The MIL-100(Fe) product was then dried in air under a fume hood at RT. Subsequently, a green reconstruction procedure of the MIL-100(Fe) sample was carried out following the work by Souza et al.[S1] with slight modifications. The previously dried MOF sample was immersed in 100 mL of deionized water and magnetically stirred (300 rpm) at RT for 48 hours. Finally, the reconstructed solid product was recovered by centrifugation and air-dried at RT.

1.2 N_2 Adsorption Measurements

N_2 adsorption measurements were carried out on the MIL-100(Fe) sample with a a Micromeritics 3Flex 3500 instrument at -196 °C. Prior to measurement, the powdered sample was outgassed at 150 °C under dynamic vacuum (10^{-3} mbar) for 24 h. The calculated Brunauer-Emmett-Teller (BET) surface area was 1439 m^2g^{-1}. Figure S2 shows the N_2 adsorption/desorption isotherms on MIL-100(Fe).

1.3 Details on the *in situ* XAS/PXRD Experiments and Data Treatment

In situ XAS-XRD measurements were performed at the BM31 beamline (Swiss-Norwegian beamlines, SNBL) at the ESRF synchrotron. The ring was operating at 6 GeV with a current of 190 mA in multibunch mode. The XAS Fe K-edge spectra were measured using a Si(111) double crystal monochromator, and a Fe foil was employed for calibration. Calibration of the PXRD patterns was performed with NIST Si powder as a standard, and raw images were azimuthally integrated with the SNBL BUBBLE software.[S2] PXRD patterns of pair distribution function (PDF) quality were collected using a 0.25448 Å wavelength and a dedicated detector configuration with an acquisition time of 60 s. A total of 10 patterns were accumulated for each PDF collection and then masked, averaged, dark-corrected and azimuthally integrated using an in-house software developed by the SNBL staff. The PDF profiles were then extracted using PDFgetX3.[S3] All extraction parameters, including the MOF composition, were kept fixed throughout the entire dataset.

For the data collection, about 7 mg of MIL-100(Fe) was loaded in a 0.7 mm quartz capillary and plugged with quartz wool. The capillary was mounted on the sample stage of the beamline and connected to the gas flow system. Subsequently, the sample was heated to 200 °C by employing a hot air blower while being exposed to a 5 mL/min pure He flux at 1 atm. All gas lines were heated at 80 °C with electrical heating tape. The sample was

maintained at 200 °C for 2 h, cooled down to RT and then exposed for 45 min to a 5 mL/min flux of He bubbled through a water saturator placed in a isothermal bath at 25 °C. After collecting the X-ray data at RT, the gas flow was switched back to pure, andhydrous He and the sample was heated up to 200 °C at a 10 °C/min rate while stopping at 50, 75, 100, 150 and 200 °C for data collection. XAS spectra and PXRD-PDF patterns were alternately recorded by automatically switching between the PXRD detector and the XAS-dedicated ion chambers at each protocol step.

Full powder pattern Le bail refinements[S4] were carried out with the GSAS-II software.[S5]

1.4 Analysis of the structural and dynamical properties of water in the MD simulations

In all MD simulations, the velocity-Verlet algorithm was employed to propagate the equations of motion, and a Nosé-Hoover thermostat was used to maintain the temperature at 25 °C. A cutoff radius of 12 Å was employed for all nonbonded interactions.

To determine the hydrogen-bonding topology in MIL-100(Fe) at increasing water loadings, two water molecules were defined as hydrogen bonded if they were located within a distance $R_{D-A} \leq 3.0$ Å between donor (D) and acceptor (A) oxygen atoms while forming an an angle $\theta_{DHA} \geq 150°$. The distribution of water molecules donating or accepting a specific number of hydrogen bonds was determined as an average over the whole NVT trajectory for each MD simulation. The same criteria were applied to evaluate the hydrogen-bonding between the MOF hydroxyl groups and the adsorbed water molecules.

The hydrogen bond time autocorrelation function was defined as:[S6]

$$C_{HB}(t) = \langle \frac{\Sigma h_{ij}(t_0) h_{ij}(t_0 + t)}{\Sigma h_{ij}(t_0)^2} \rangle \tag{1}$$

where h_{ij} is a measure of the presence ($h_{ij} = 1$) or not ($h_{ij} = 0$) of a hydrogen bond between

atoms i and j, while the summation is performed over all possible water oxygen atom pairings ij and the function is averaged over many different trajectory time origins t_0.[S6–S8]

To obtain insights on how the reorientational ability of water changes as a function of water loadings, the O-H orientational function, $C_{2,OH}$, was calculated as:

$$C_{2,OH} = \langle P_2[\hat{\mathbf{u}}(t=0) \cdot \hat{\mathbf{u}}(t)] \rangle \tag{2}$$

where $P_2[\hat{\mathbf{u}}(t=0) \cdot \hat{\mathbf{u}}(t)]$ is the second-order Legendre polynomial of the angle formed by the OH unit vector of a specific H_2O molecule at a certain time t and the unit vector of the same molecule at $t = 0$.[S9] The brackets indicate an ensemble average over both OH bonds of all water molecules. $C_{2,OH}$ may be taken as an indication of the orientational anisotropy of water molecules, which relax (and thus lose orientational correlation) faster when they are not linked by hydrogen bonds.

The Mean Squared Displacement (MSD) calculation was computed with the Einstein formula:

$$MSD = \langle \Delta r(t)^2 \rangle \tag{3}$$

where $r(t)$ is the position of a given water molecule at time t and $\Delta r(t)$ is the displacement after a certain time amount t. For each given water loading, the MSD was calculated as an average over the free water molecules and the given NVT trajectory. Both the $C_{2,OH}$ and MSD calculations were performed by dividing the first 500 ps of the 1 ns NVE trajectory into ten segments of 50 ps each. MSD and $C_{2,OH}$ were then computed for each segment and averaged to improve the statistical accuracy of the results. All of the above-mentioned analyses, in addition to radial distribution function calculations, were carried out using the MDAnalysis python package.[S7,S8]

The uniform manifold approximation and projection (UMAP) analysis was carried out using the latest python implementation of the UMAP algorithm (umap-learn).[S10] UMAP is a newly developed dimension reduction technique that can be exploited for data visualiza-

tion,[S11,S12] which is based on the assumpions that (i) the data lie on a uniformly distributed Riemannian manifold, (ii) the Riemannian metric is at least approximately locally constant and (iii) the manifold is locally connected. Following these assumptions, topological considerations yield a low-dimensional projection of the data that has the closest possible equivalent fuzzy topological structure. In the simplest application of UMAP, this low-dimensional space has only two dimensions, named $UMAP_0$ and $UMAP_1$: these do not bear any physical or statistical meaning, at variance with principal component analysis, and should only be interpreted as abstract coordinates that capture the relationships between data points in the original high-dimensional space, and therefore maximize the degree of clustering between similar data.

1.5 *Ab Initio* XAS Calculations

The Fe K-edge absorption spectra were calculated using the FDMNES code,[S13,S14] within the muffin-tin approximation to estimate the cluster potential and the real Hedin-Lundqvist self-energy correction.[S15] Specifically, the absorption cross section $\sigma(\omega)$ was calculated as:

$$\sigma(\omega) = 4\pi^2\alpha\hbar\omega \sum_j \sum_{f,g} |\langle\Psi_f|\Theta|\Psi_g^{(j)}\rangle|^2 \delta(\hbar\omega - (E - E_g^{(j)})) \quad (4)$$

where $\hbar\omega$ is the energy of the photon, α the fine structure constant, E_g and E are the energies of the ground state $\Psi_g^{(j)}$ and Ψ_f, respectively, while the summation over j includes the contribution of all the atoms in the unit cells possessing index j.[S16]

The calculated cross-sections were convoluted in a post-processing step by an energy-dependent arctangent function (Γ) in order to account for the spectral broadening due to core-hole lifetime and photoelectron kinetic energy losses effects. Γ is defined as follows:

$$\Gamma = \Gamma_i + \Gamma_f(\frac{1}{2} + \frac{1}{\pi}arctan(\frac{\pi\Gamma_f}{3E_w}(\frac{\boldsymbol{E} - E_f}{E_c} - \frac{E_c^2}{(\boldsymbol{E} - E_f)^2}))) \quad (5)$$

where $\boldsymbol{E}$ is the energy scale of the Fe K–edge NEXAFS spectrum, Γ_i and Γ_f are the core-level and final-state widths, respectively, E_c and E_w are the center and width of the arctangent function, respectively, while E_f is the Fermi energy.[S17]

2 Supplementary Figures (Figures S1–S52)

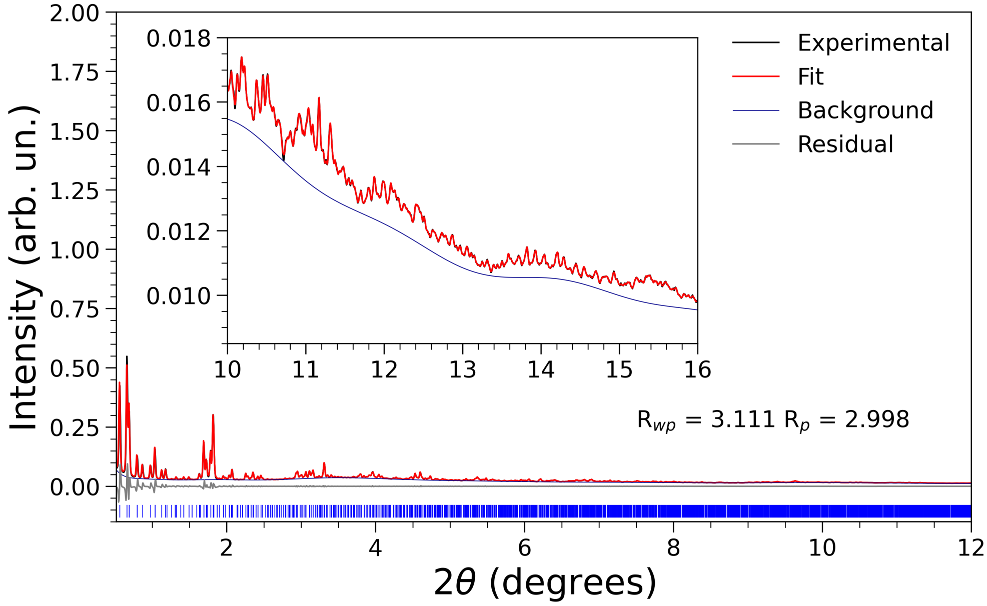

Figure S1: Whole powder pattern Le Bail refinement for the synchrotron PXRD pattern collected on the MIL-100(Fe) sample in air prior to thermal activation. Experimental, calculated and difference curves are reported in black, red, and gray, respectively. The positions of the calculated Bragg reflections are indicated by vertical blue bars. The figures of merit of the Le Bail refinement are reported in the plot. Space Group: Fd$\bar{3}$m, a=73.086 Å, λ=0.25448 Å.

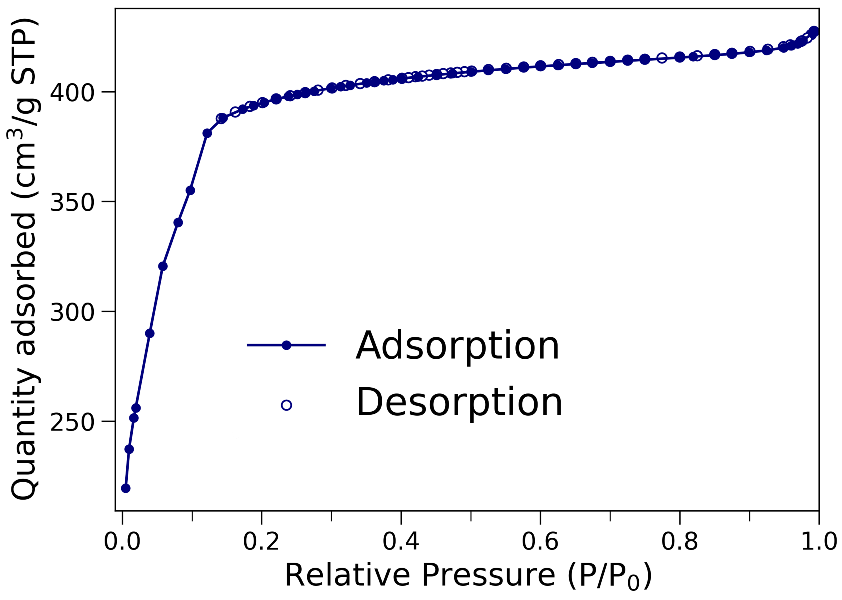

Figure S2: N_2 adsorption isotherm collected at -196 °C for MIL-100(Fe) activated under vacuum at 150 °C for 24 hours. BET surface area: 1439 m^2g^{-1}.

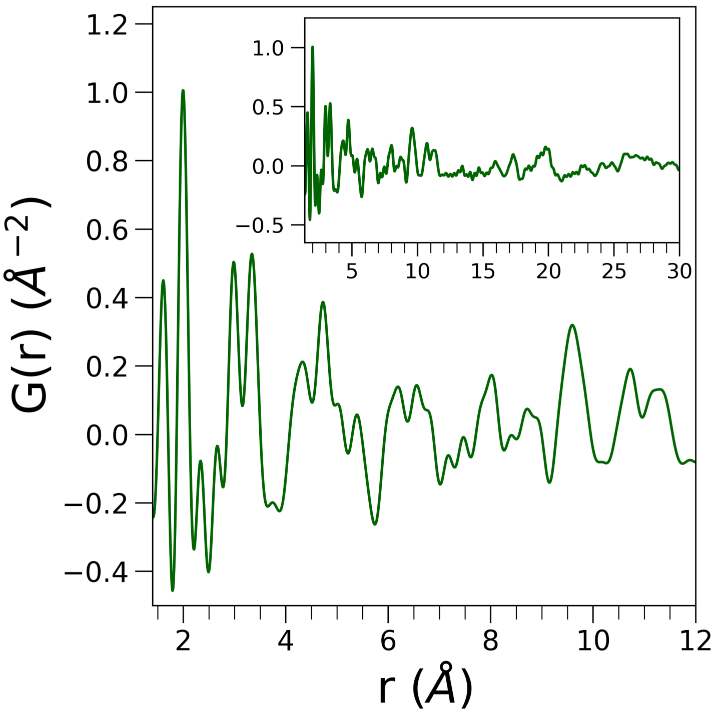

Figure S3: X-ray PDF of pristine MIL-100(Fe) collected at RT in ambient air.

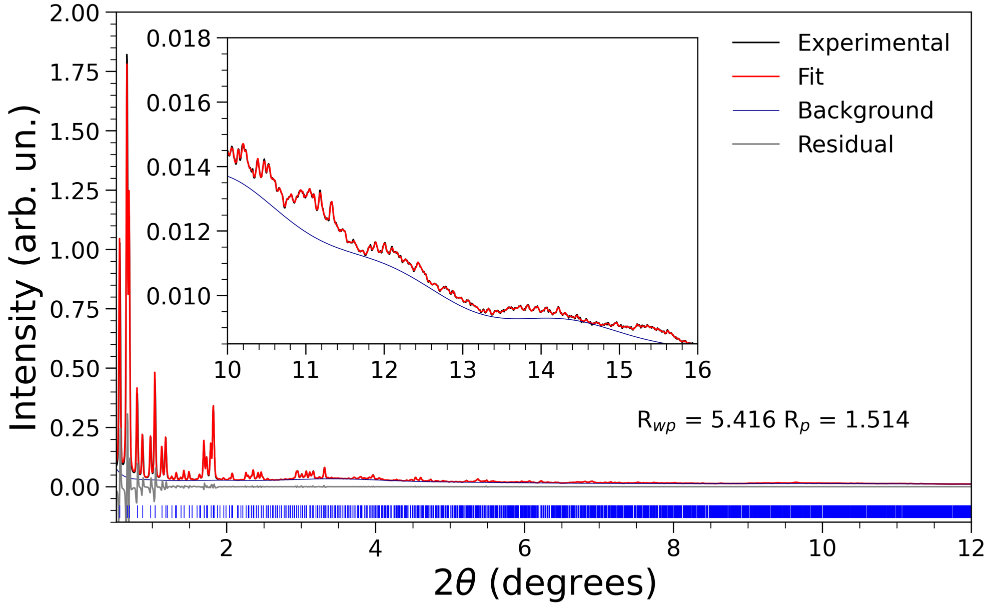

Figure S4: Whole powder pattern Le Bail refinement for the synchrotron PXRD pattern collected on the MIL-100(Fe) at 25°C in pure He flux during the thermal activation procedure. Experimental, calculated and difference curves are reported in black, red, and gray, respectively. The positions of the calculated Bragg reflections are indicated by vertical blue bars. The figures of merit of the Le Bail refinement are reported in the plot. λ=0.25448 Å.

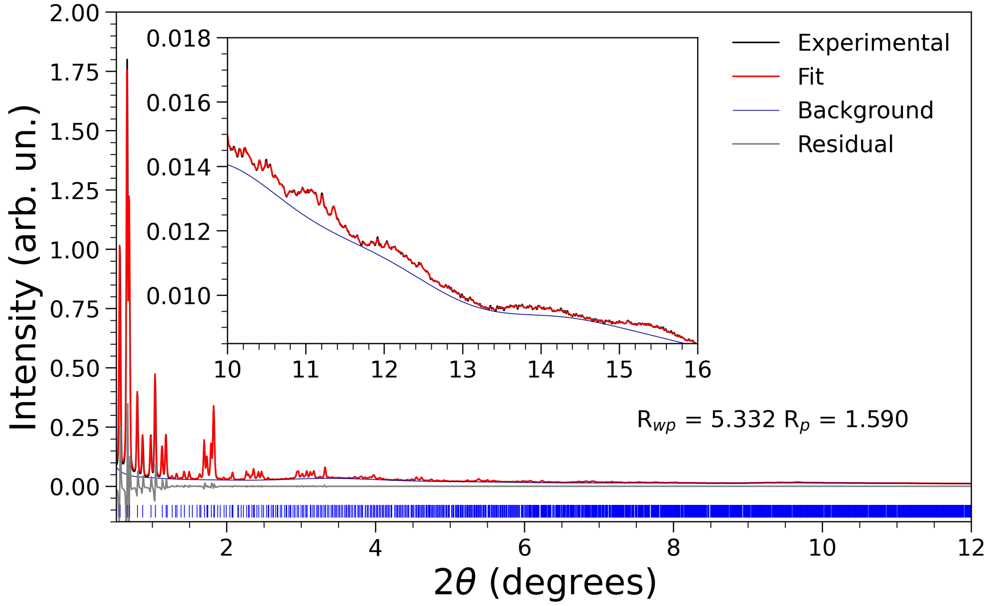

Figure S5: Whole powder pattern Le Bail refinement for the synchrotron PXRD pattern collected on the MIL-100(Fe) at 50°C in pure He flux during the thermal activation procedure. Experimental, calculated and difference curves are reported in black, red, and gray, respectively. The positions of the calculated Bragg reflections are indicated by vertical blue bars. The figures of merit of the Le Bail refinement are reported in the plot. λ=0.25448 Å.

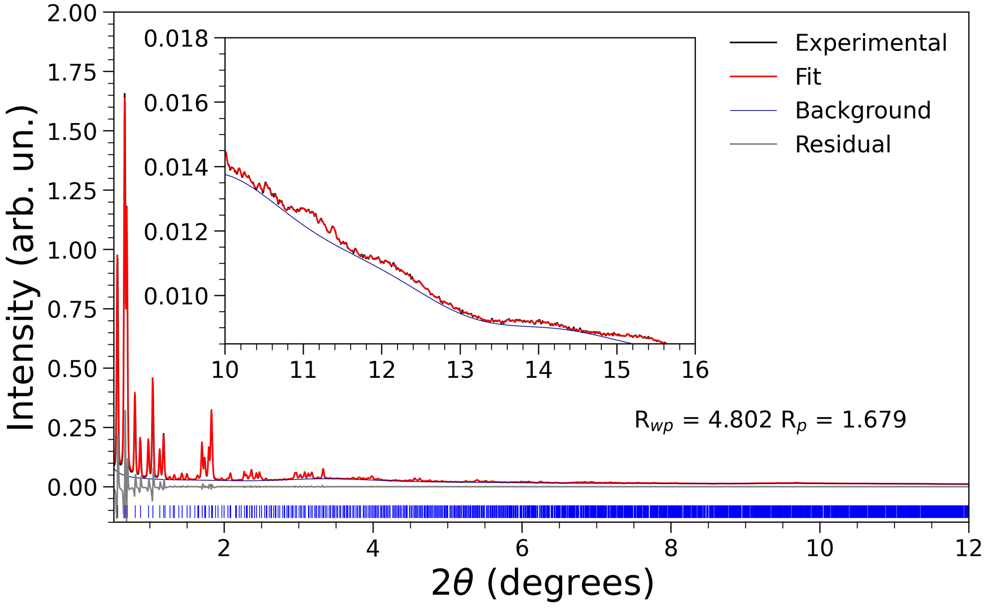

Figure S6: Whole powder pattern Le Bail refinement for the synchrotron PXRD pattern collected on the MIL-100(Fe) at 100°C in pure He flux during the thermal activation procedure. Experimental, calculated and difference curves are reported in black, red, and gray, respectively. The positions of the calculated Bragg reflections are indicated by vertical blue bars. The figures of merit of the Le Bail refinement are reported in the plot. λ=0.25448 Å.

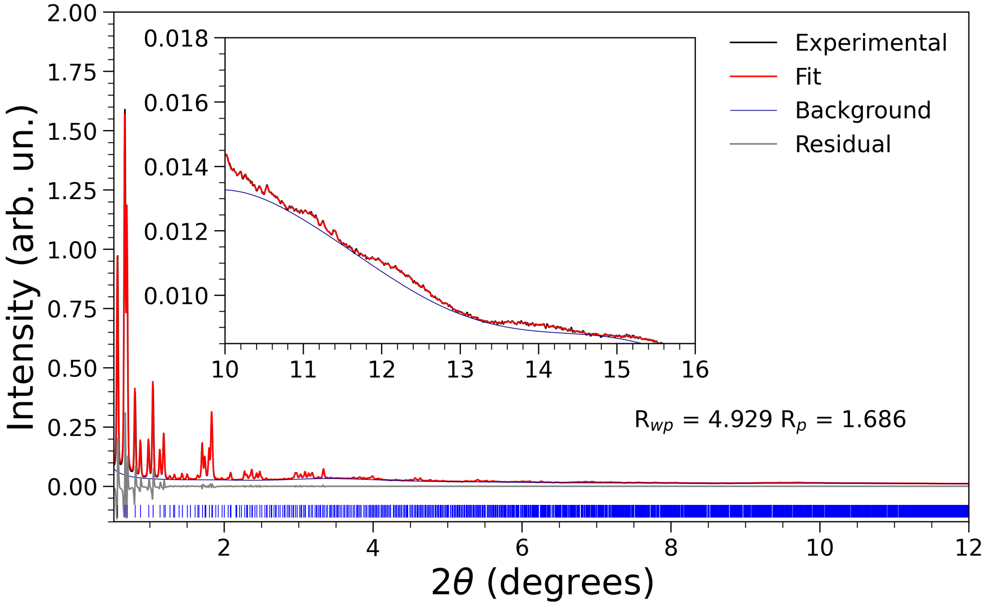

Figure S7: Whole powder pattern Le Bail refinement for the synchrotron PXRD pattern collected on the MIL-100(Fe) at 150°C in pure He flux during the thermal activation procedure. Experimental, calculated and difference curves are reported in black, red, and gray, respectively. The positions of the calculated Bragg reflections are indicated by vertical blue bars. The figures of merit of the Le Bail refinement are reported in the plot. λ=0.25448 Å.

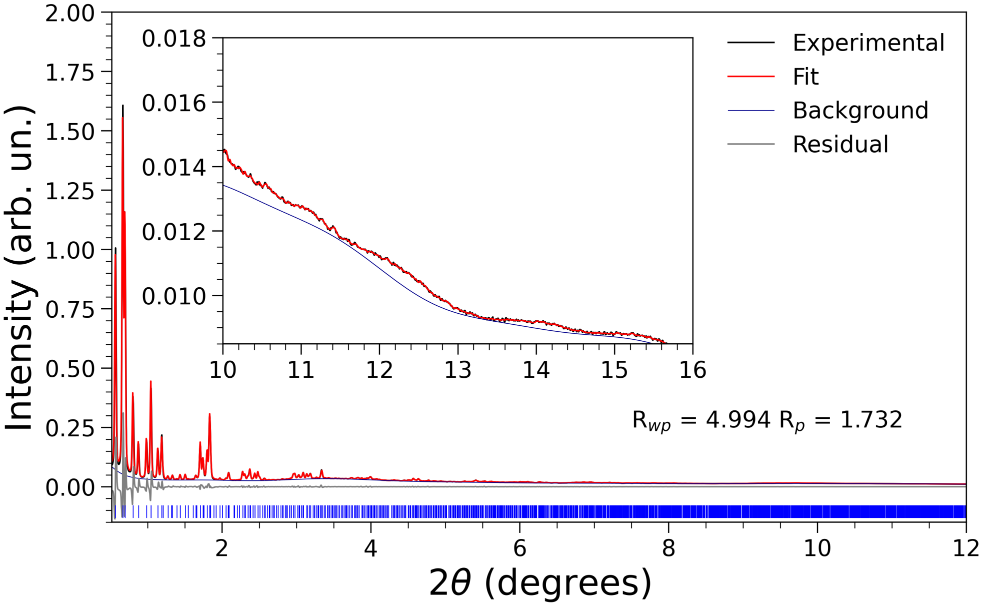

Figure S8: Whole powder pattern Le Bail refinement for the synchrotron PXRD pattern collected on the MIL-100(Fe) at 200°C in pure He flux during the thermal activation procedure. Experimental, calculated and difference curves are reported in black, red, and gray, respectively. The positions of the calculated Bragg reflections are indicated by vertical blue bars. The figures of merit of the Le Bail refinement are reported in the plot. λ=0.25448 Å.

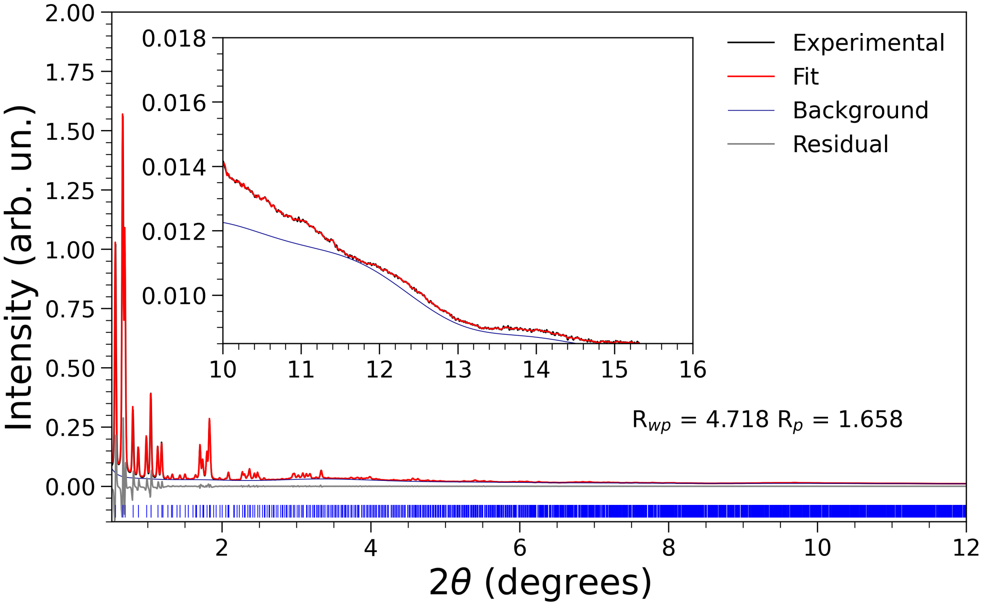

Figure S9: Whole powder pattern Le Bail refinement for the synchrotron PXRD pattern collected on the MIL-100(Fe) thermally treated at 200 °C in pure He flux for 2 h. Experimental, calculated and difference curves are reported in black, red, and gray, respectively. The positions of the calculated Bragg reflections are indicated by vertical blue bars. The figures of merit of the Le Bail refinement are reported in the plot. λ=0.25448 Å.

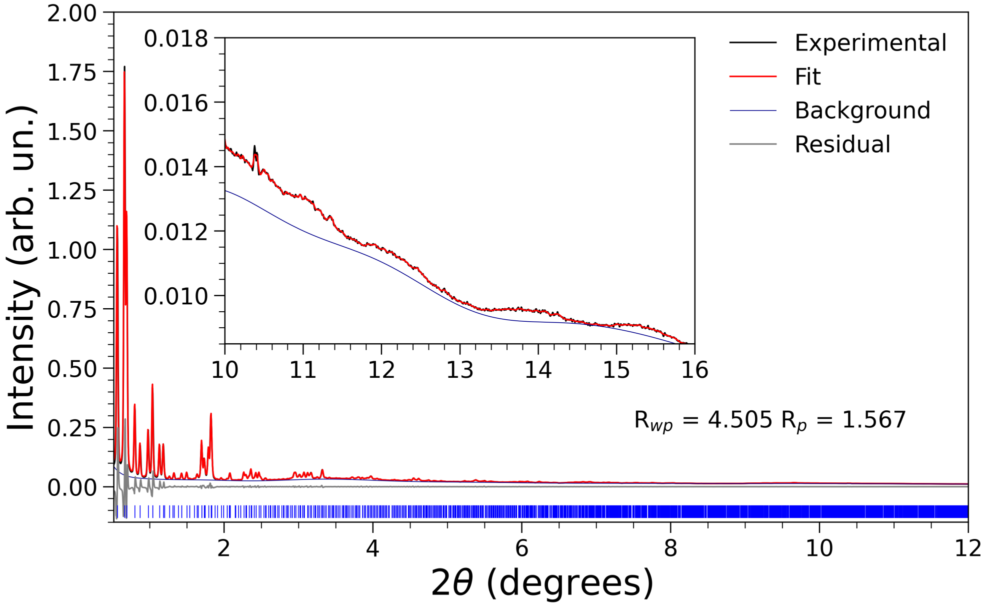

Figure S10: Whole powder pattern Le Bail refinement for the synchrotron PXRD pattern collected on the MIL-100(Fe) at 25 °C in pure He flux after the thermal activation procedure. Experimental, calculated and difference curves are reported in black, red, and gray, respectively. The positions of the calculated Bragg reflections are indicated by vertical blue bars. The figures of merit of the Le Bail refinement are reported in the plot. λ=0.25448 Å.

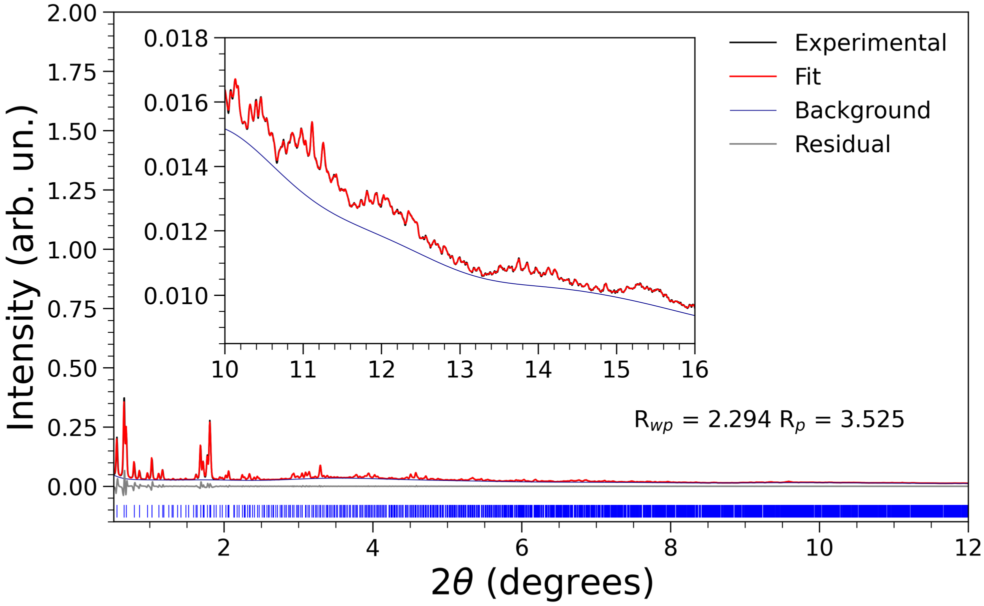

Figure S11: Whole powder pattern Le Bail refinement for the synchrotron PXRD pattern collected on the MIL-100(Fe) at 25 °C in a water-saturated He flux. Experimental, calculated and difference curves are reported in black, red, and gray, respectively. The positions of the calculated Bragg reflections are indicated by vertical blue bars. The figures of merit of the Le Bail refinement are reported in the plot. λ=0.25448 Å.

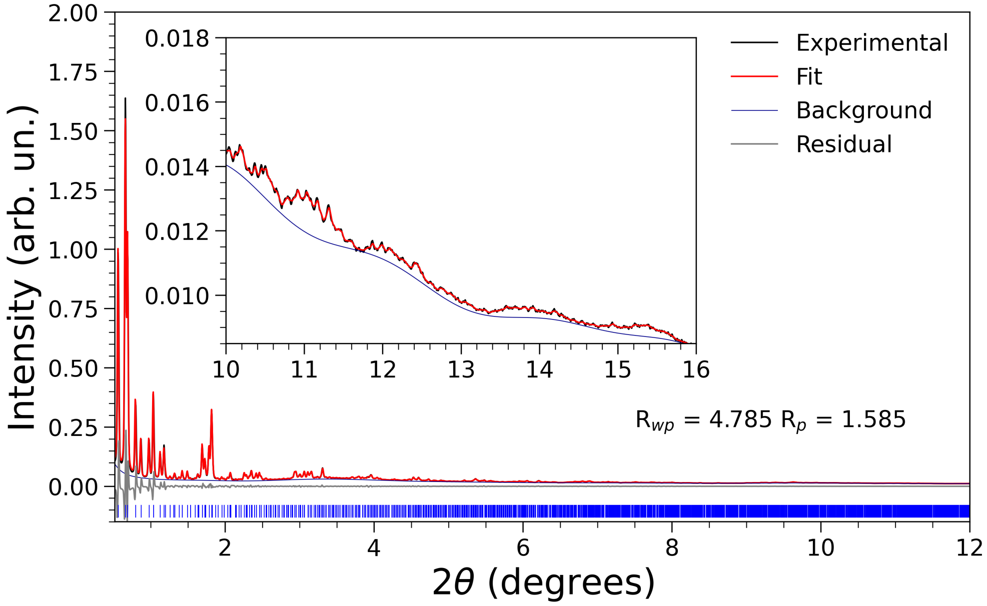

Figure S12: Whole powder pattern Le Bail refinement for the synchrotron PXRD pattern collected on the MIL-100(Fe) at 25°C in pure He flux during the thermal dehydration procedure. Experimental, calculated and difference curves are reported in black, red, and gray, respectively. The positions of the calculated Bragg reflections are indicated by vertical blue bars. The figures of merit of the Le Bail refinement are reported in the plot. λ=0.25448 Å.

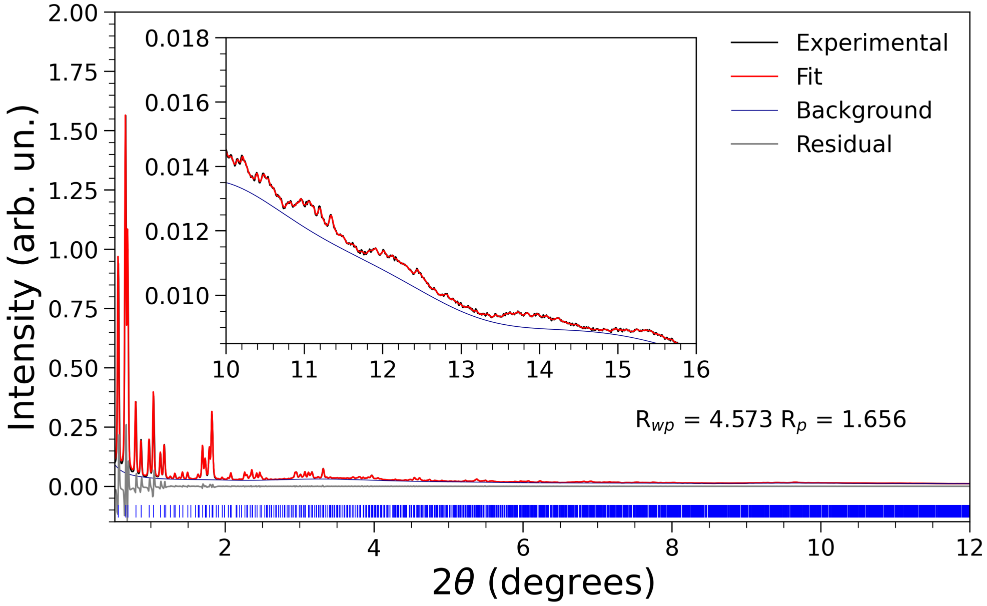

Figure S13: Whole powder pattern Le Bail refinement for the synchrotron PXRD pattern collected on the MIL-100(Fe) at 50°C in pure He flux during the thermal dehydration procedure. Experimental, calculated and difference curves are reported in black, red, and gray, respectively. The positions of the calculated Bragg reflections are indicated by vertical blue bars. The figures of merit of the Le Bail refinement are reported in the plot. λ=0.25448 Å.

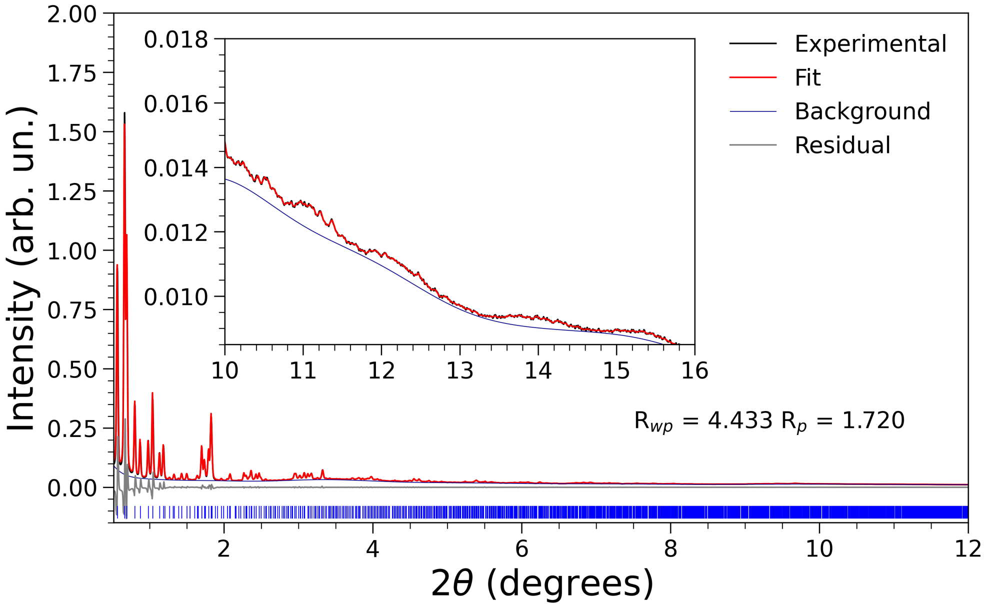

Figure S14: Whole powder pattern Le Bail refinement for the synchrotron PXRD pattern collected on the MIL-100(Fe) at 100°C in pure He flux during the thermal dehydration procedure. Experimental, calculated and difference curves are reported in black, red, and gray, respectively. The positions of the calculated Bragg reflections are indicated by vertical blue bars. The figures of merit of the Le Bail refinement are reported in the plot. λ=0.25448 Å.

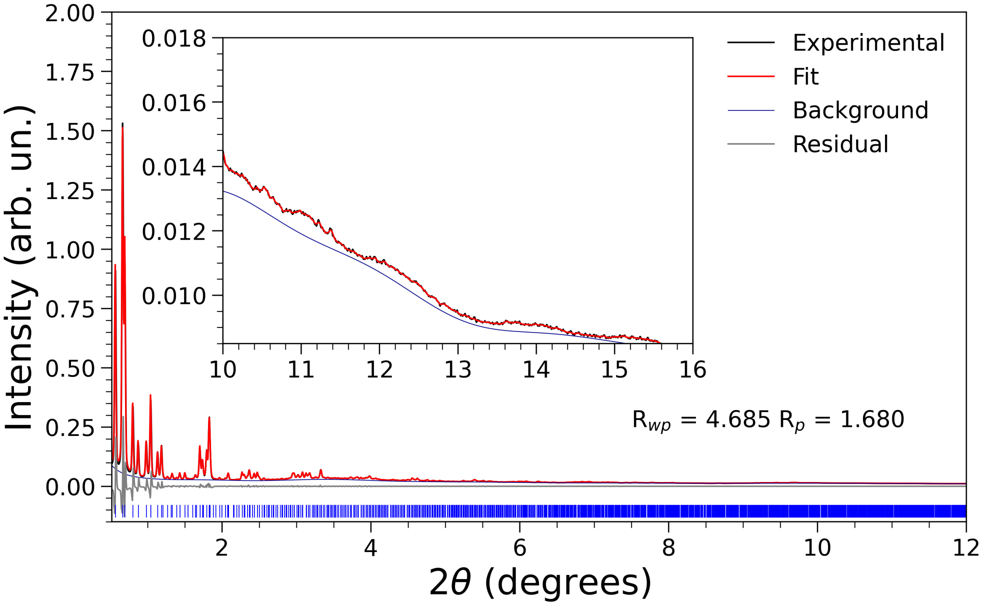

Figure S15: Whole powder pattern Le Bail refinement for the synchrotron PXRD pattern collected on the MIL-100(Fe) at 150°C in pure He flux during the thermal dehydration procedure. Experimental, calculated and difference curves are reported in black, red, and gray, respectively. The positions of the calculated Bragg reflections are indicated by vertical blue bars. The figures of merit of the Le Bail refinement are reported in the plot. λ=0.25448 Å.

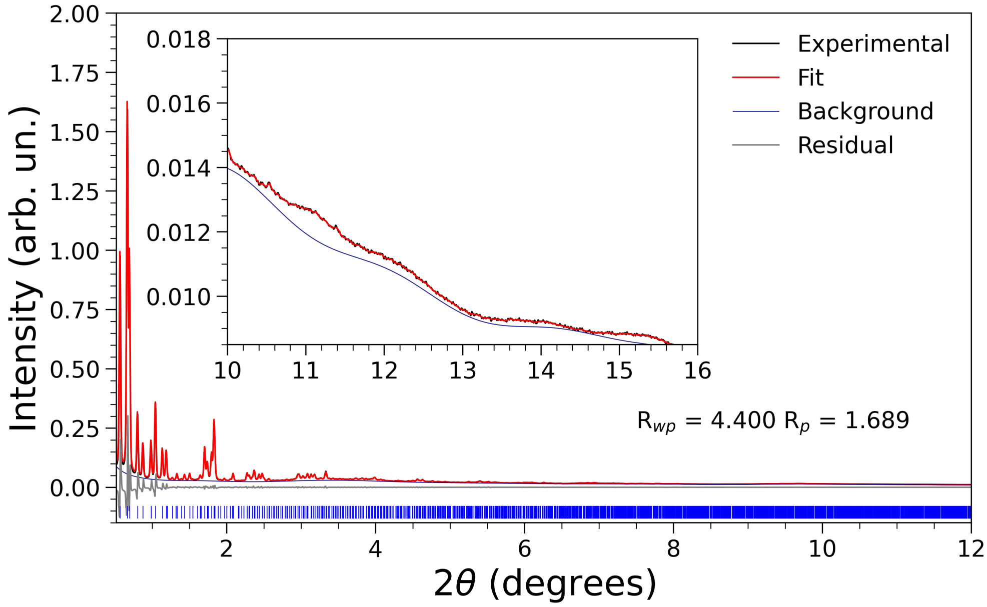

Figure S16: Whole powder pattern Le Bail refinement for the synchrotron PXRD pattern collected on the MIL-100(Fe) at 200°C in pure He flux during the thermal dehydration procedure. Experimental, calculated and difference curves are reported in black, red, and gray, respectively. The positions of the calculated Bragg reflections are indicated by vertical blue bars. The figures of merit of the Le Bail refinement are reported in the plot. λ=0.25448 Å.

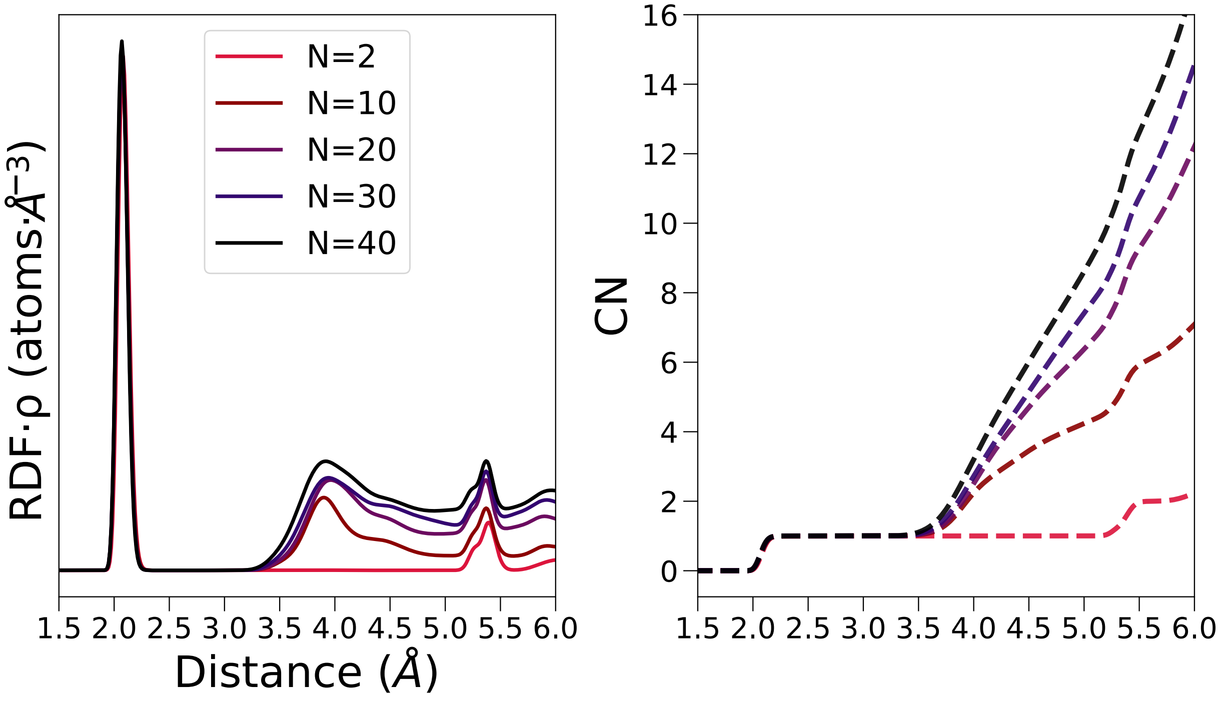

Figure S17: Fe-O radial distribution functions multiplied by the numerical densities of the observed atoms (left panel) calculated between the iron sites of MIL-100(Fe) and the oxygen of water molecules at increasing water loadings (N=2 - N=40) and corresponding coordination numbers (right panel).

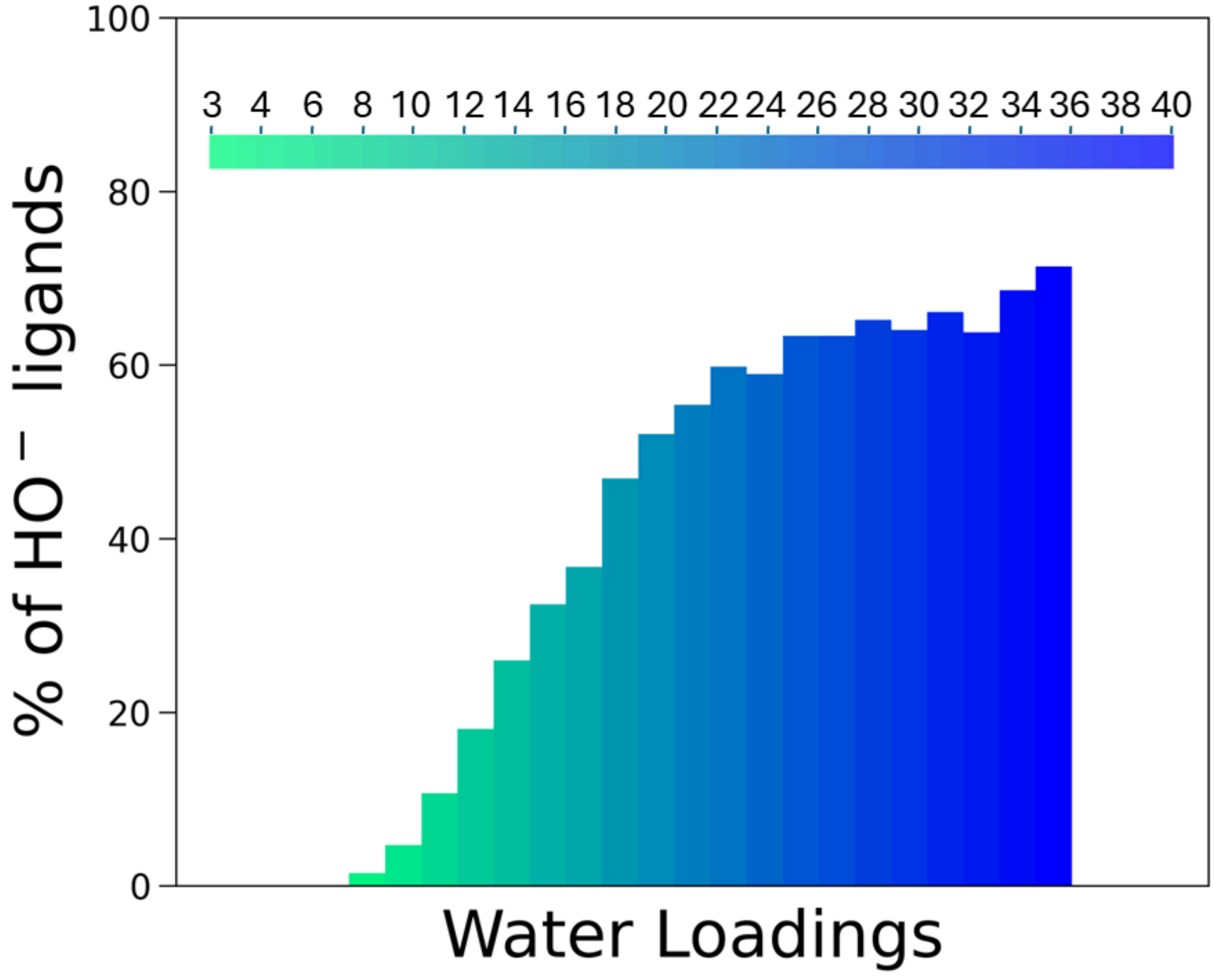

Figure S18: Percentage of hydroxyl ligands of the MOF framework that donate a hydrogen bond to water molecules, as calculated from MD simulations of MIL-100(Fe) at increasing water loadings. The considered water loadings are listed above with the color bar.

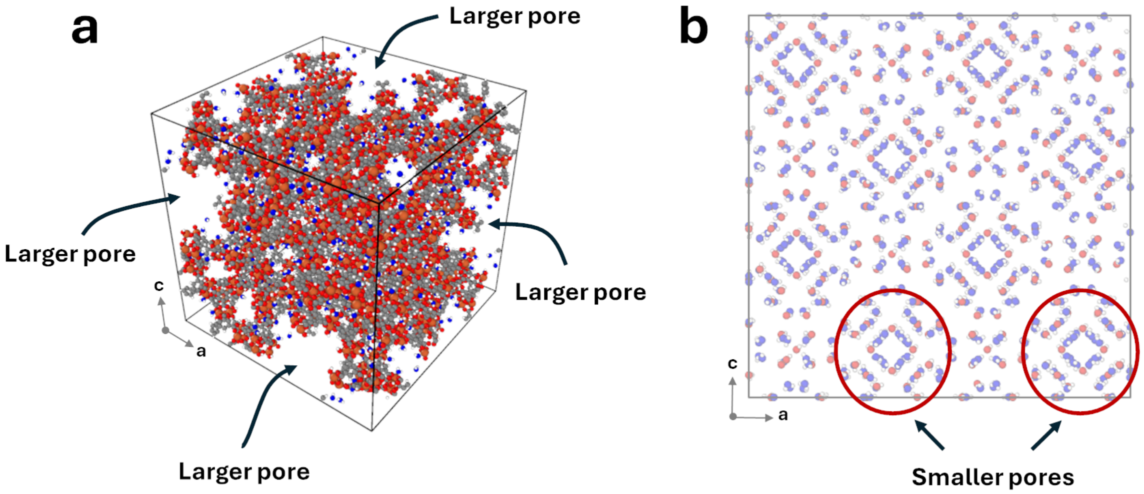

Figure S19: Side (a) and front (b) views of MIL-100(Fe) at a N=2 water loading. Examples of the larger (a) and smaller (b) MOF pore environents are indicated in the two panels. In panel (b), solely the hydroxyl ligands and water molecules are displayed for visualization purposes.

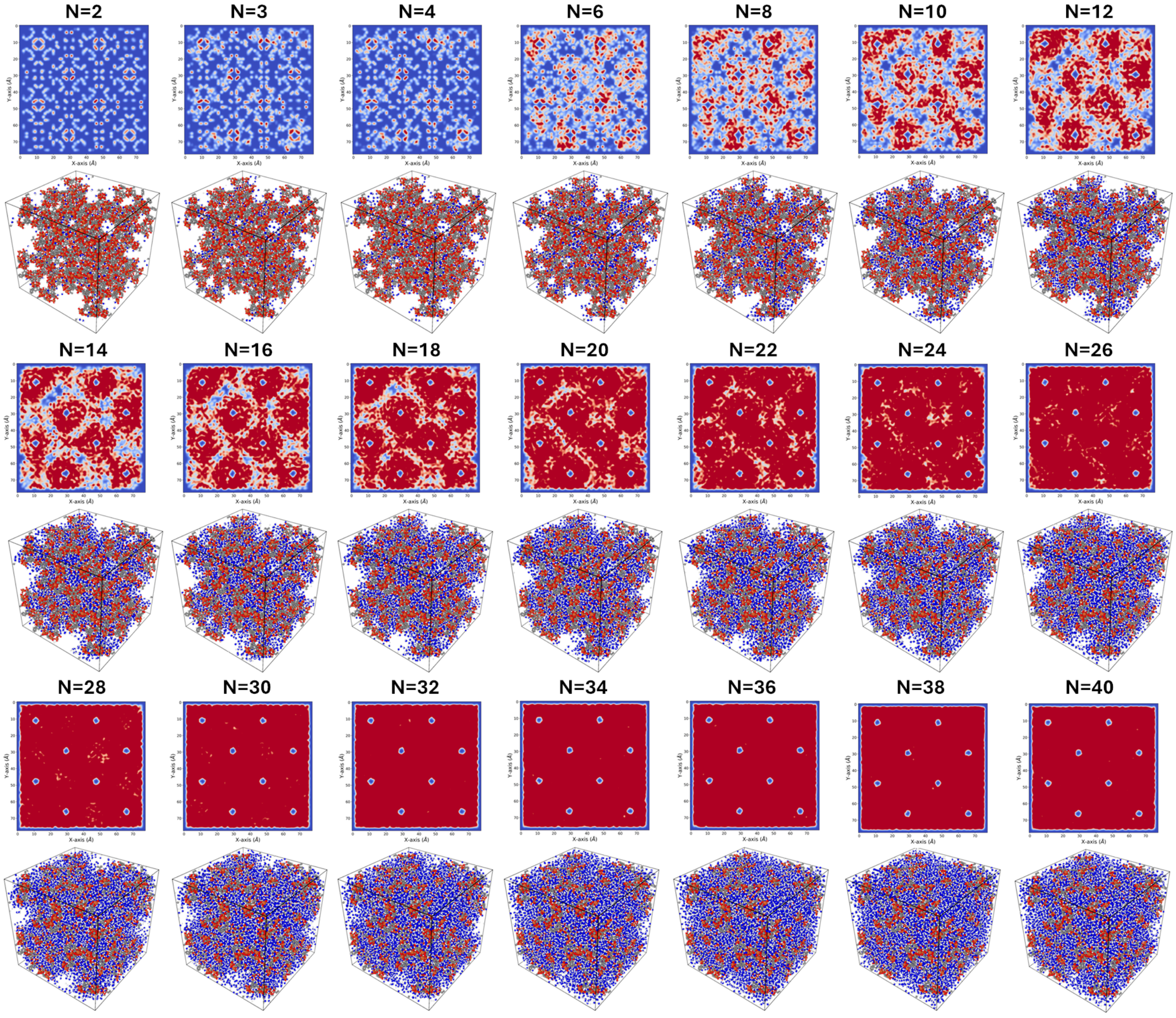

Figure S20: MD snapshots of MIL-100(Fe) loaded with N=2 to N=40 water molecules (top panels). The oxygen atoms of the water molecules are displayed in blue. Density maps of water (bottom panels) calculated from MD simulations of MIL-100(Fe) loaded with N=2 to N=40 water molecules. Darker red colors are used to depict regions with higher water density.

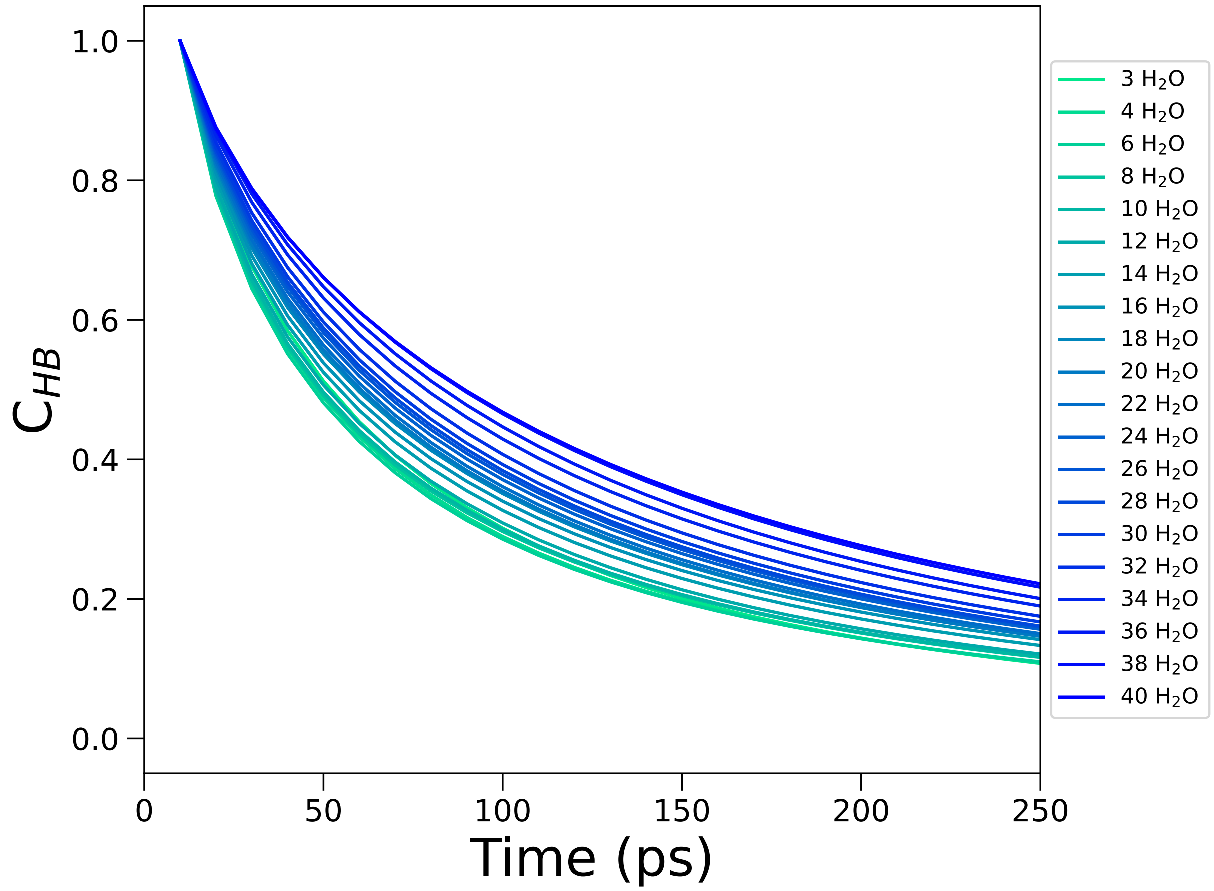

Figure S21: Hydrogen bond time autocorrelation functions obtained from MD simulations of MIL-100(Fe) at increasing water loadings.

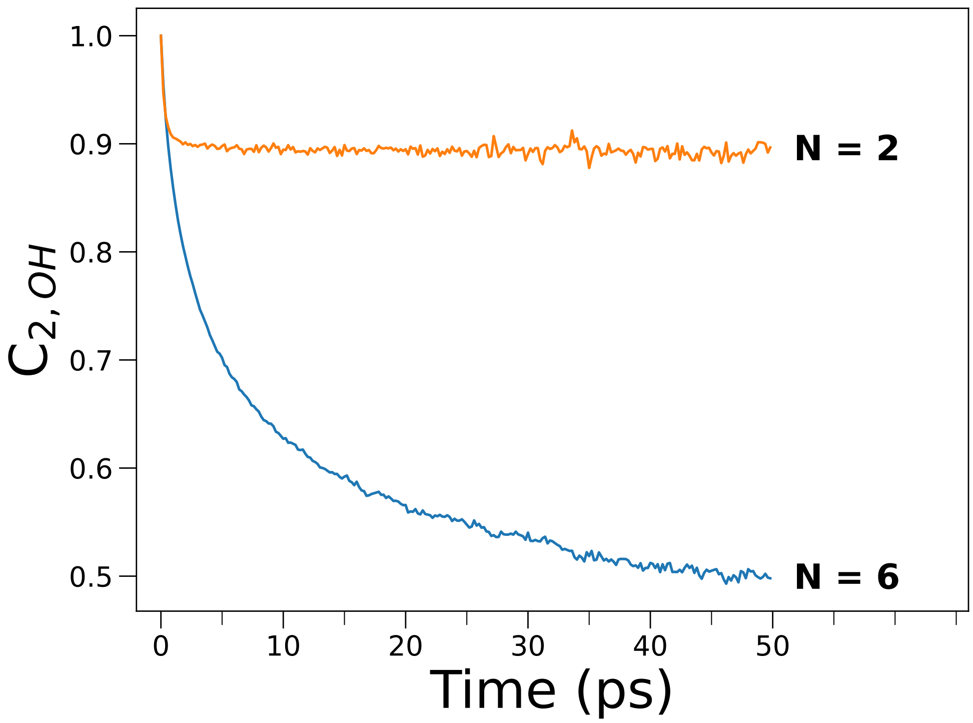

Figure S22: Water orientational correlation functions obtained from MD simulations of MIL-100(Fe) at water loadings of N=2 and N=6.

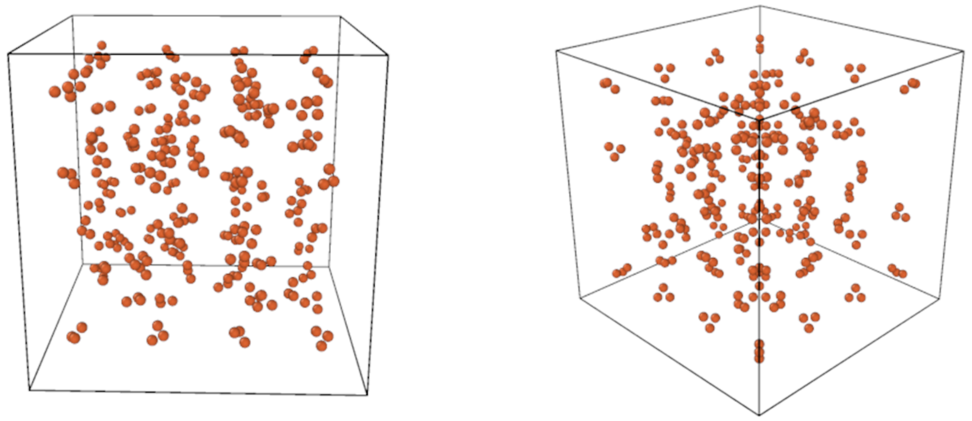

Figure S23: Distinct Fe trimeric units (amounting to 94 in total) located within the unit cell of MIL-100(Fe) and selected for the theoretical XAS calculations.

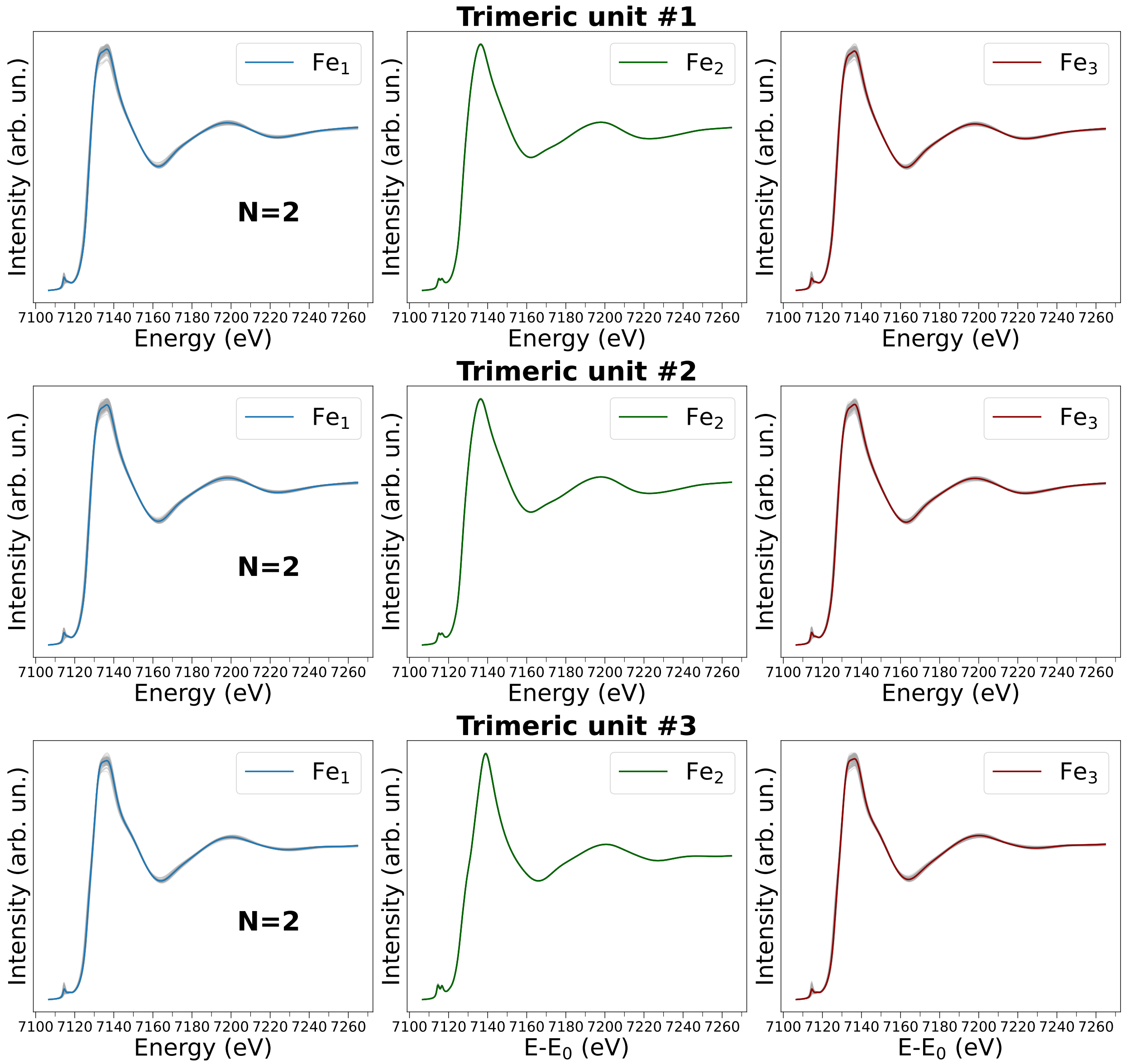

Figure S24: Theoretical Fe K-edge XANES spectra (gray lines) calculated from 100 MD snapshots of MIL-100(Fe) loaded with N=2 water molecules per trimeric unit and converged XANES averages of the 100 spectra (full lines). The three rows display the XANES spectra evaluated for three distinct trimeric units in the MOF unit cell. The three columns display the XANES spectra calculated for each individual Fe site in the given trimeric unit (Fe_1/Fe_3 and Fe_2 correspond to the Fe sites directly coordinated by water and hydroxyl ligands, respectively).

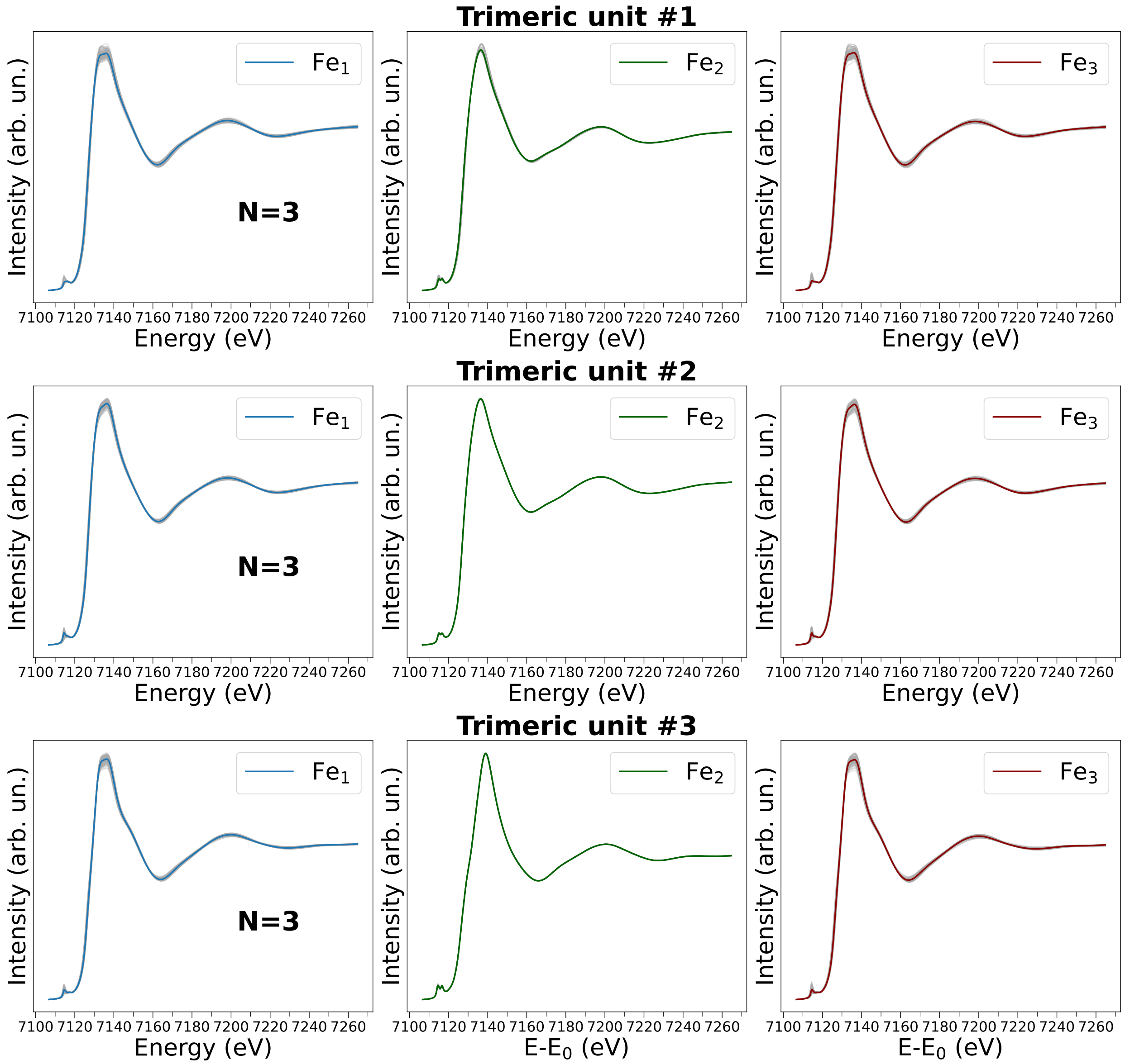

Figure S25: Theoretical Fe K-edge XANES spectra (gray lines) calculated from 100 MD snapshots of MIL-100(Fe) loaded with N=3 water molecules per trimeric unit and converged XANES averages of the 100 spectra (full lines). The three rows display the XANES spectra evaluated for three distinct trimeric units in the MOF unit cell. The three columns display the XANES spectra calculated for each individual Fe site in the given trimeric unit (Fe_1/Fe_3 and Fe_2 correspond to the Fe sites directly coordinated by water and hydroxyl ligands, respectively).

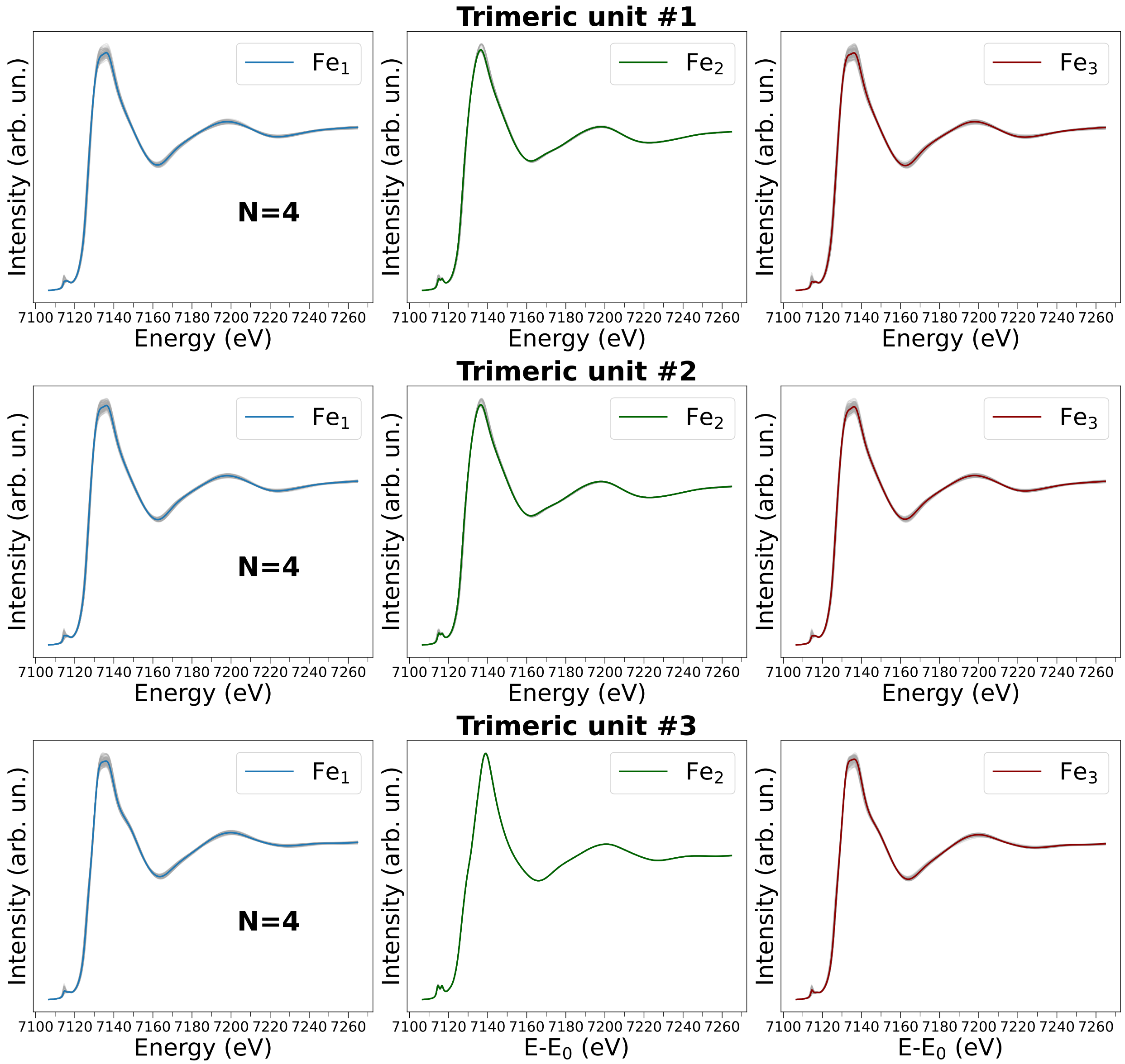

Figure S26: Theoretical Fe K-edge XANES spectra (gray lines) calculated from 100 MD snapshots of MIL-100(Fe) loaded with N=4 water molecules per trimeric unit and converged XANES averages of the 100 spectra (full lines). The three rows display the XANES spectra evaluated for three distinct trimeric units in the MOF unit cell. The three columns display the XANES spectra calculated for each individual Fe site in the given trimeric unit (Fe_1/Fe_3 and Fe_2 correspond to the Fe sites directly coordinated by water and hydroxyl ligands, respectively).

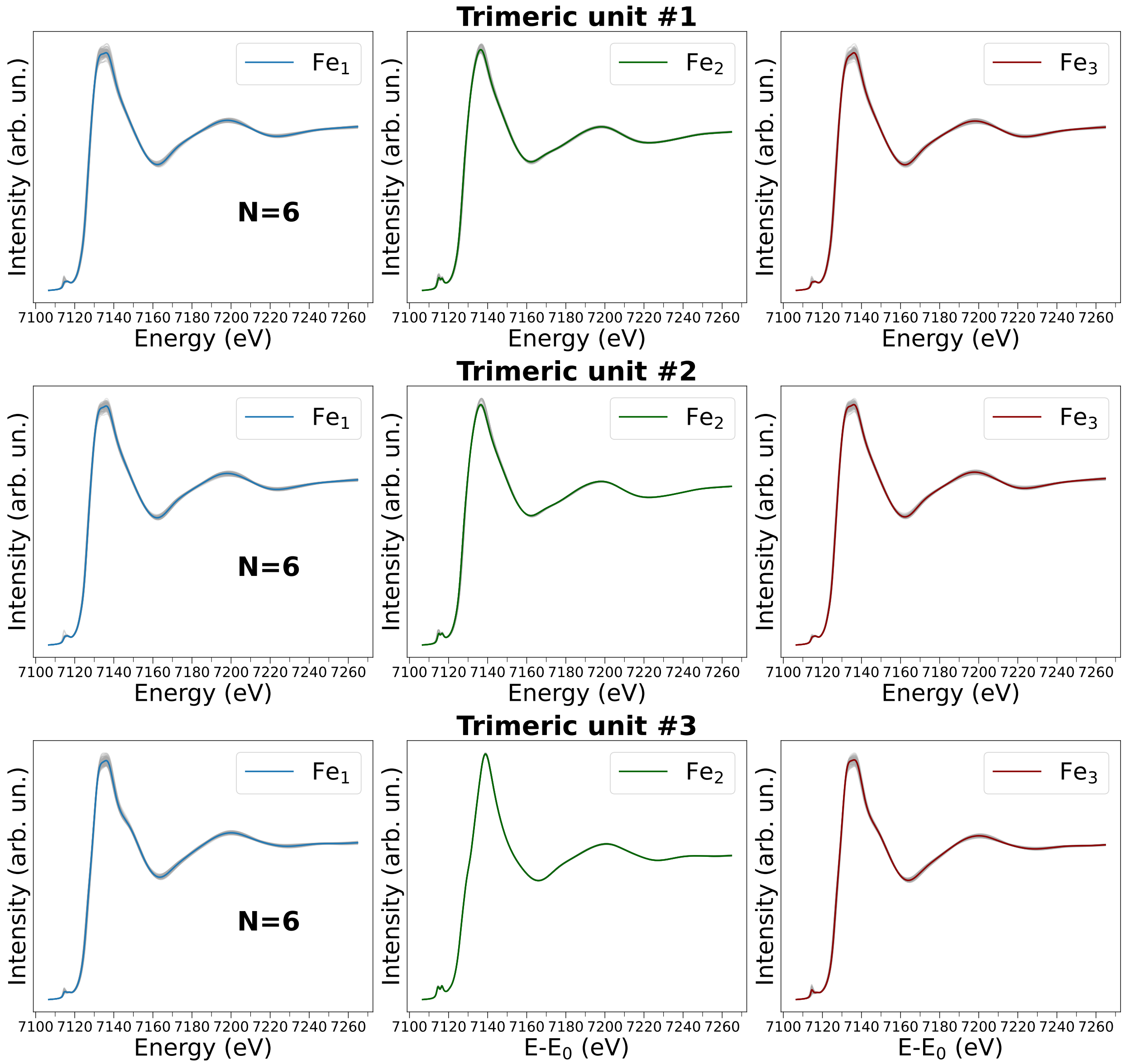

Figure S27: Theoretical Fe K-edge XANES spectra (gray lines) calculated from 100 MD snapshots of MIL-100(Fe) loaded with N=6 water molecules per trimeric unit and converged XANES averages of the 100 spectra (full lines). The three rows display the XANES spectra evaluated for three distinct trimeric units in the MOF unit cell. The three columns display the XANES spectra calculated for each individual Fe site in the given trimeric unit (Fe_1/Fe_3 and Fe_2 correspond to the Fe sites directly coordinated by water and hydroxyl ligands, respectively).

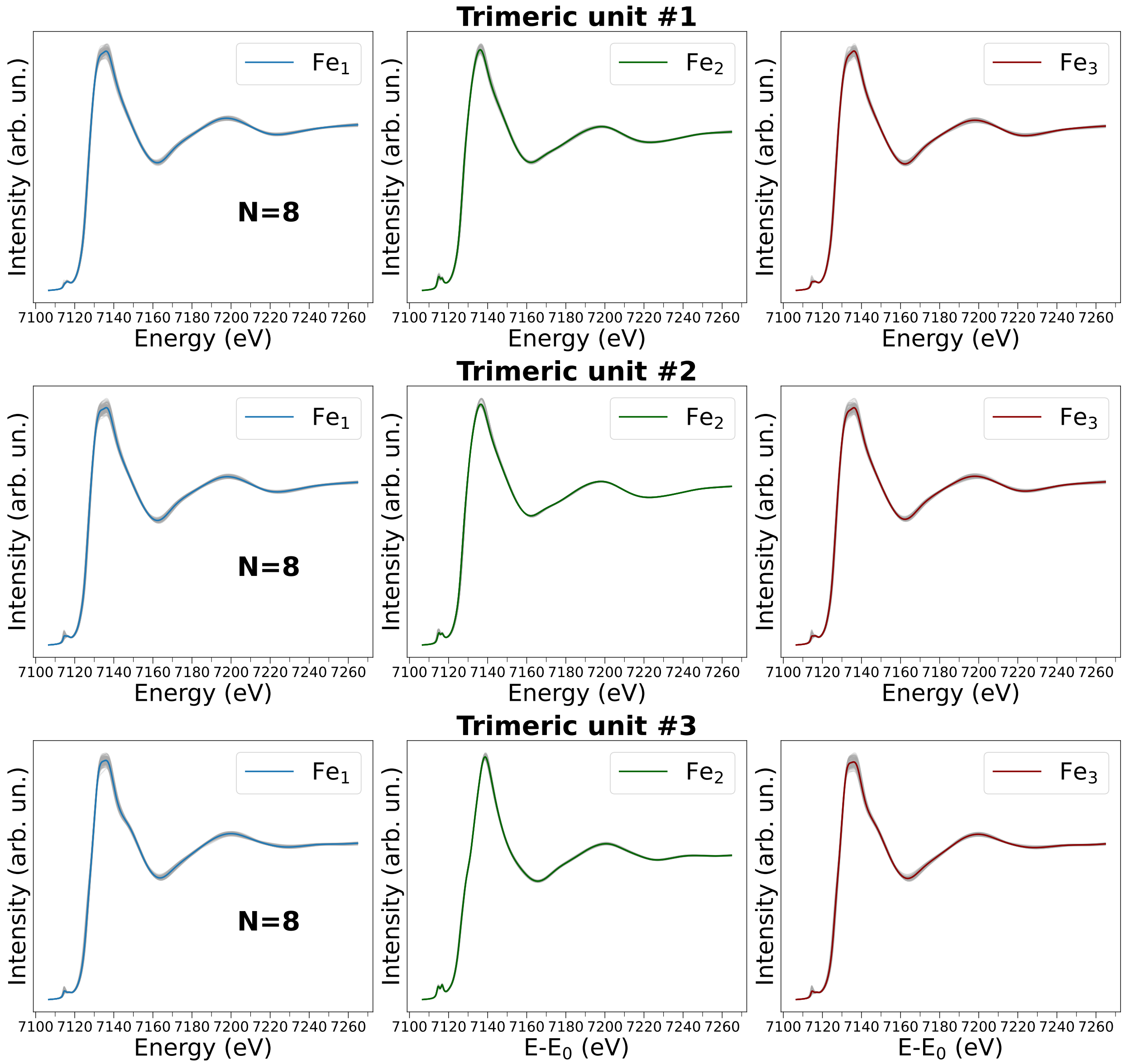

Figure S28: Theoretical Fe K-edge XANES spectra (gray lines) calculated from 100 MD snapshots of MIL-100(Fe) loaded with N=8 water molecules per trimeric unit and converged XANES averages of the 100 spectra (full lines). The three rows display the XANES spectra evaluated for three distinct trimeric units in the MOF unit cell. The three columns display the XANES spectra calculated for each individual Fe site in the given trimeric unit (Fe_1/Fe_3 and Fe_2 correspond to the Fe sites directly coordinated by water and hydroxyl ligands, respectively).

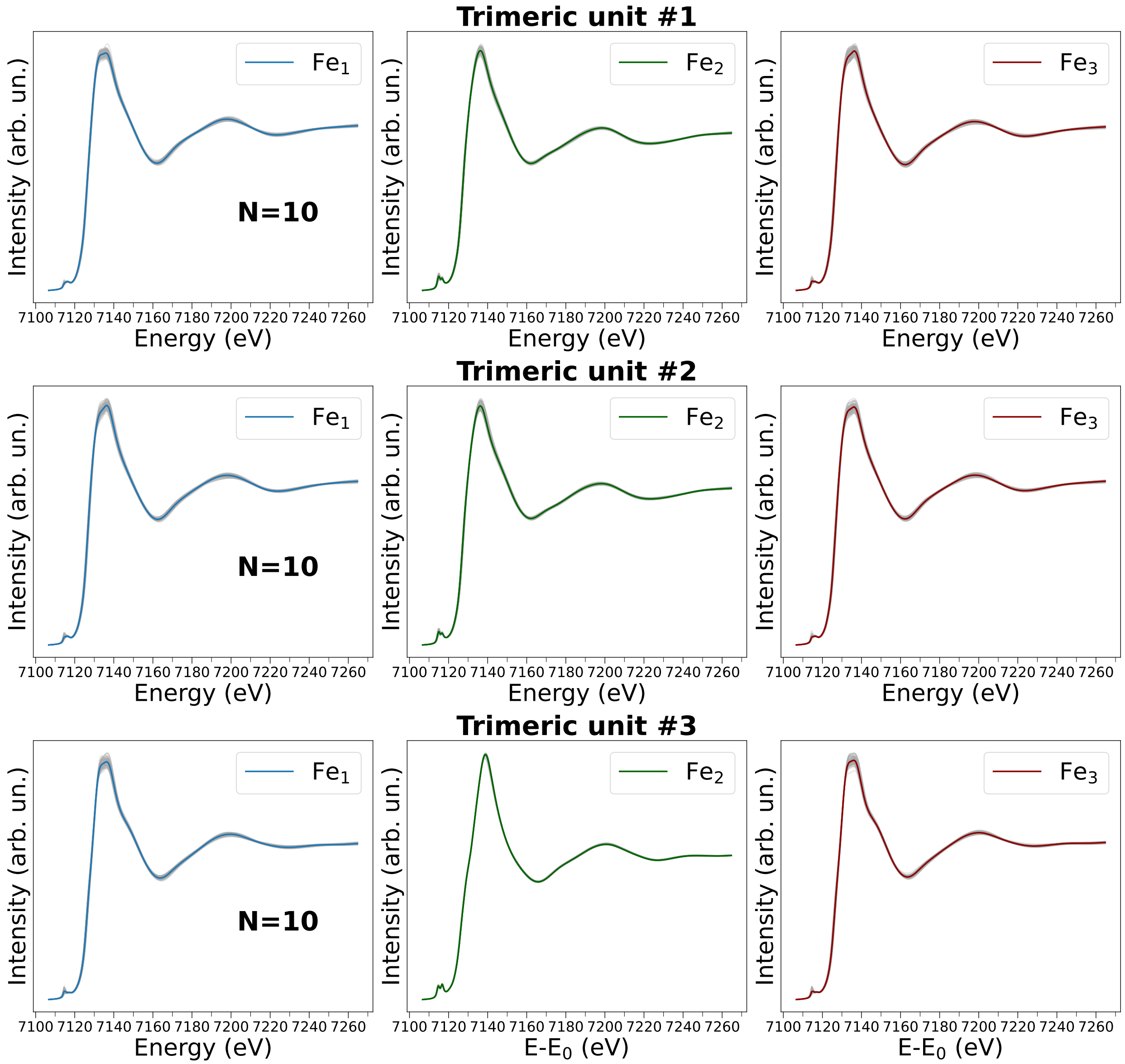

Figure S29: Theoretical Fe K-edge XANES spectra (gray lines) calculated from 100 MD snapshots of MIL-100(Fe) loaded with N=10 water molecules per trimeric unit and converged XANES averages of the 100 spectra (full lines). The three rows display the XANES spectra evaluated for three distinct trimeric units in the MOF unit cell. The three columns display the XANES spectra calculated for each individual Fe site in the given trimeric unit (Fe_1/Fe_3 and Fe_2 correspond to the Fe sites directly coordinated by water and hydroxyl ligands, respectively).

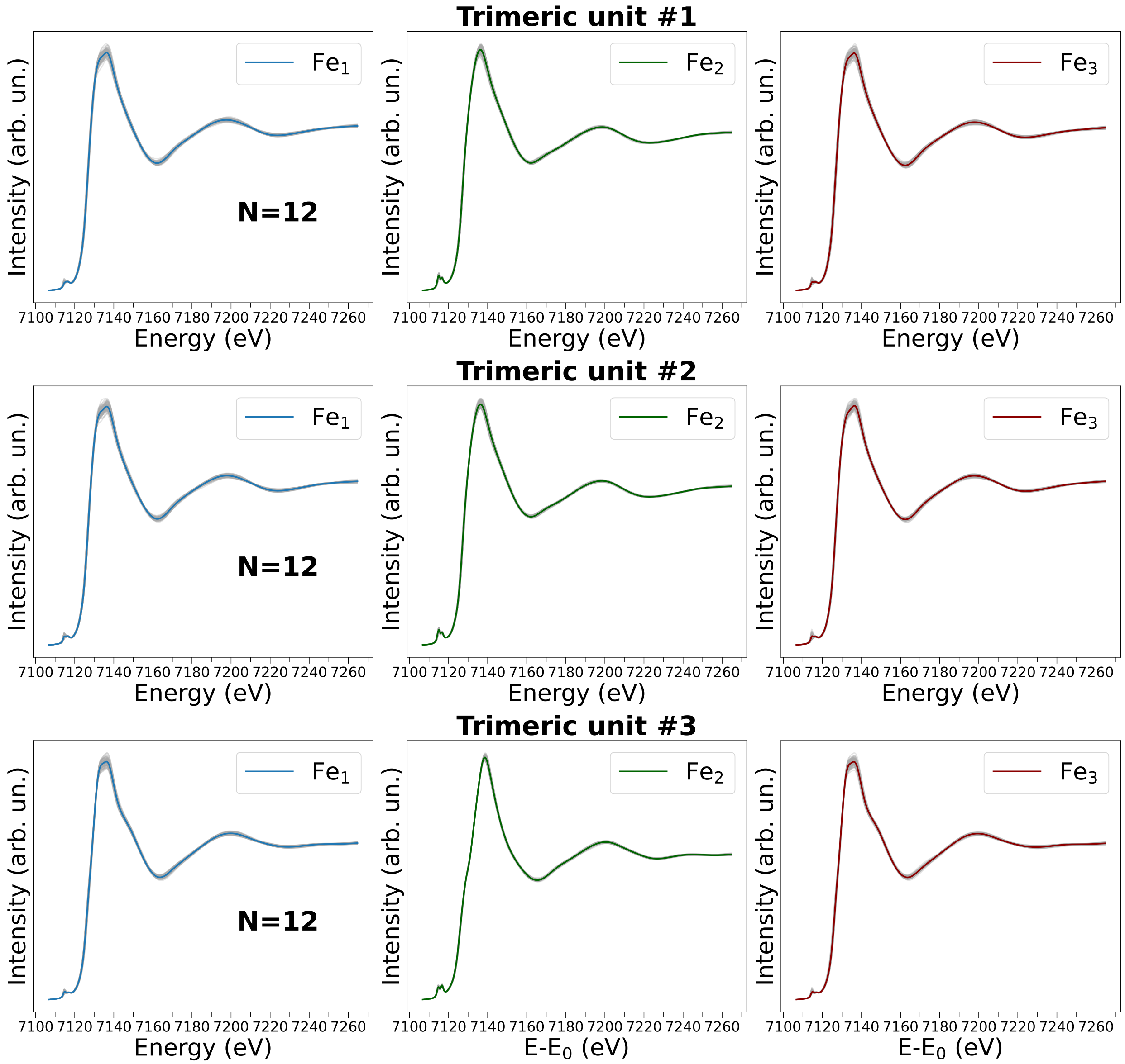

Figure S30: Theoretical Fe K-edge XANES spectra (gray lines) calculated from 100 MD snapshots of MIL-100(Fe) loaded with N=12 water molecules per trimeric unit and converged XANES averages of the 100 spectra (full lines). The three rows display the XANES spectra evaluated for three distinct trimeric units in the MOF unit cell. The three columns display the XANES spectra calculated for each individual Fe site in the given trimeric unit (Fe_1/Fe_3 and Fe_2 correspond to the Fe sites directly coordinated by water and hydroxyl ligands, respectively).

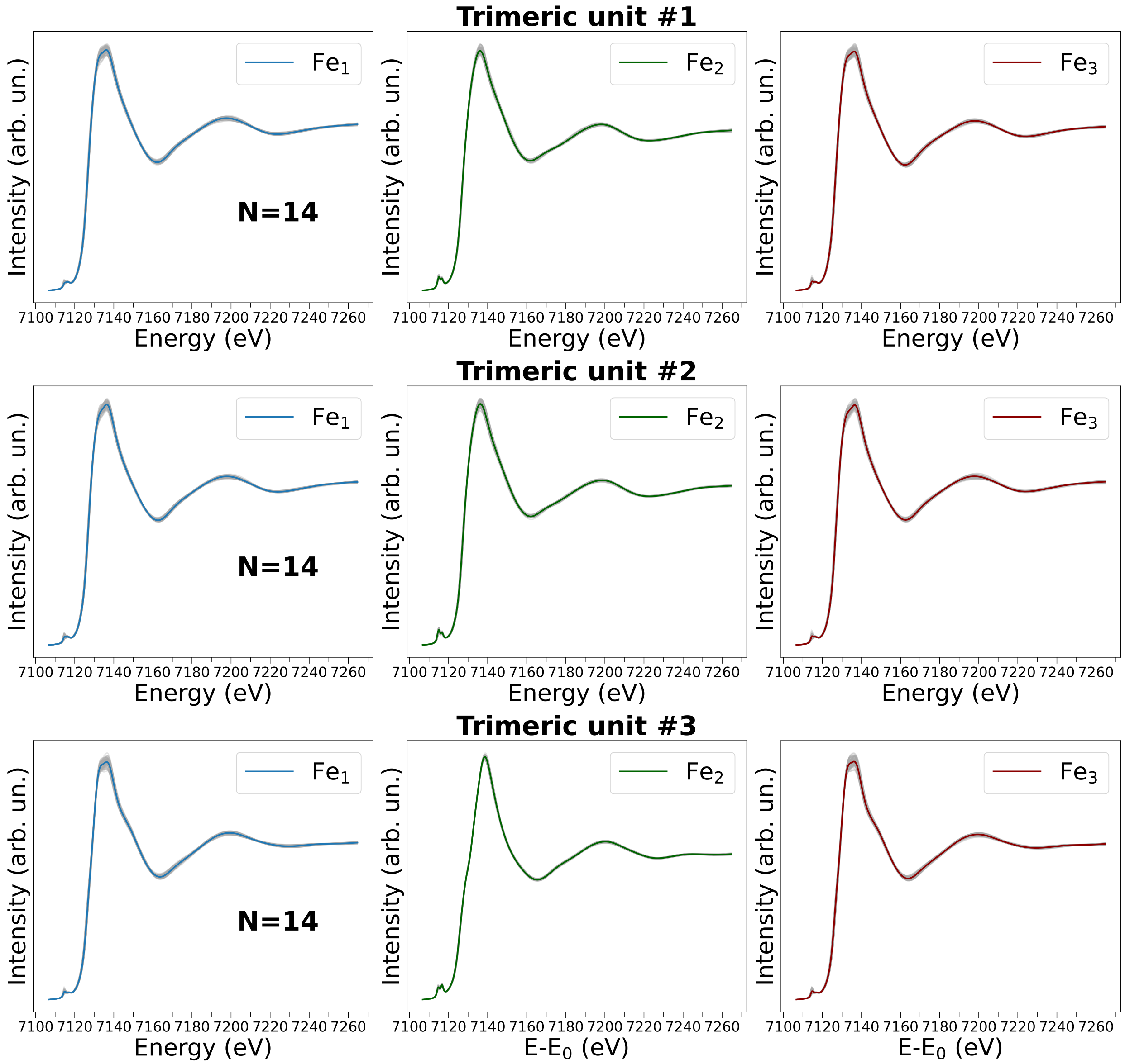

Figure S31: Theoretical Fe K-edge XANES spectra (gray lines) calculated from 100 MD snapshots of MIL-100(Fe) loaded with N=14 water molecules per trimeric unit and converged XANES averages of the 100 spectra (full lines). The three rows display the XANES spectra evaluated for three distinct trimeric units in the MOF unit cell. The three columns display the XANES spectra calculated for each individual Fe site in the given trimeric unit (Fe_1/Fe_3 and Fe_2 correspond to the Fe sites directly coordinated by water and hydroxyl ligands, respectively).

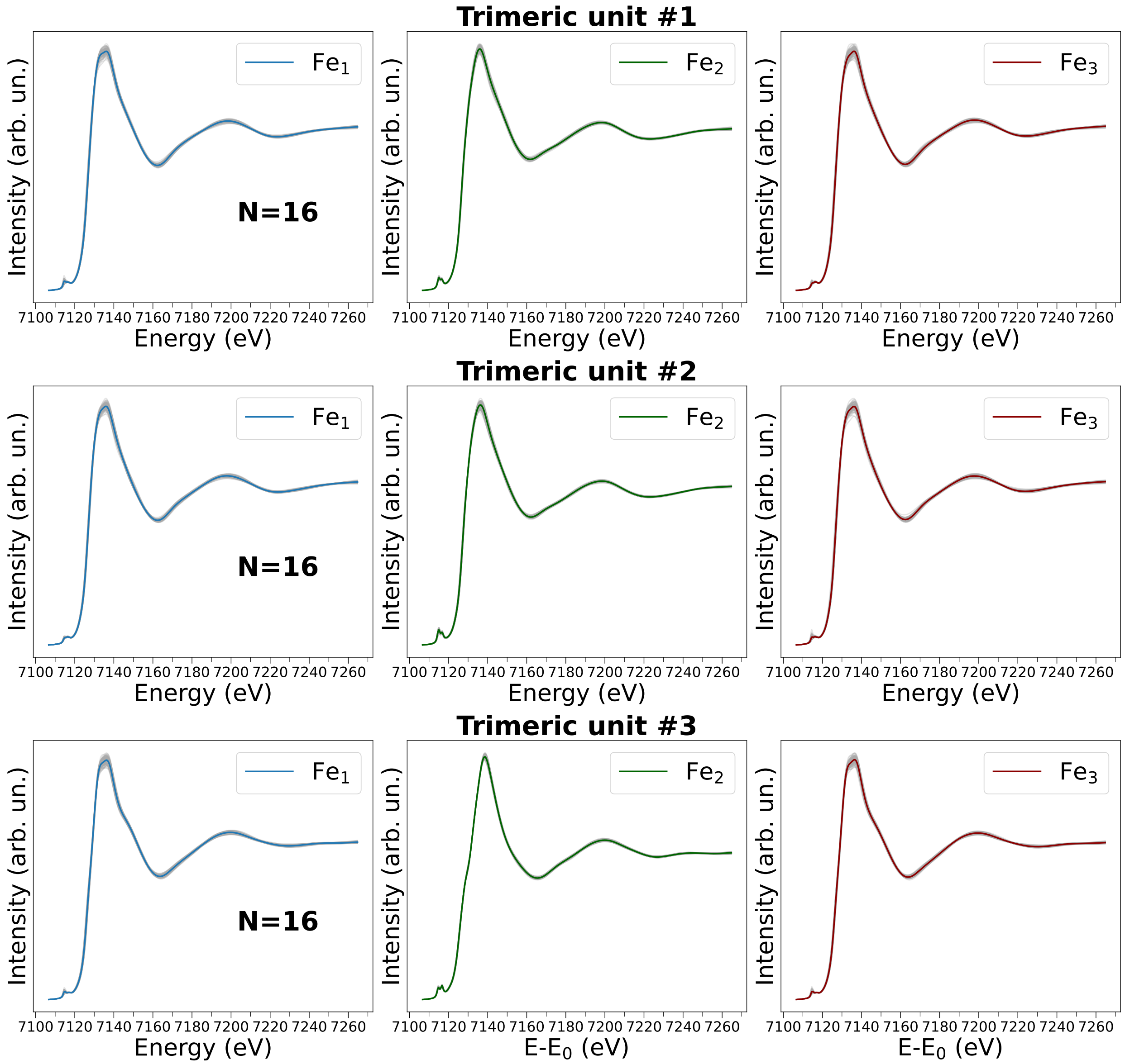

Figure S32: Theoretical Fe K-edge XANES spectra (gray lines) calculated from 100 MD snapshots of MIL-100(Fe) loaded with N=16 water molecules per trimeric unit and converged XANES averages of the 100 spectra (full lines). The three rows display the XANES spectra evaluated for three distinct trimeric units in the MOF unit cell. The three columns display the XANES spectra calculated for each individual Fe site in the given trimeric unit (Fe_1/Fe_3 and Fe_2 correspond to the Fe sites directly coordinated by water and hydroxyl ligands, respectively).

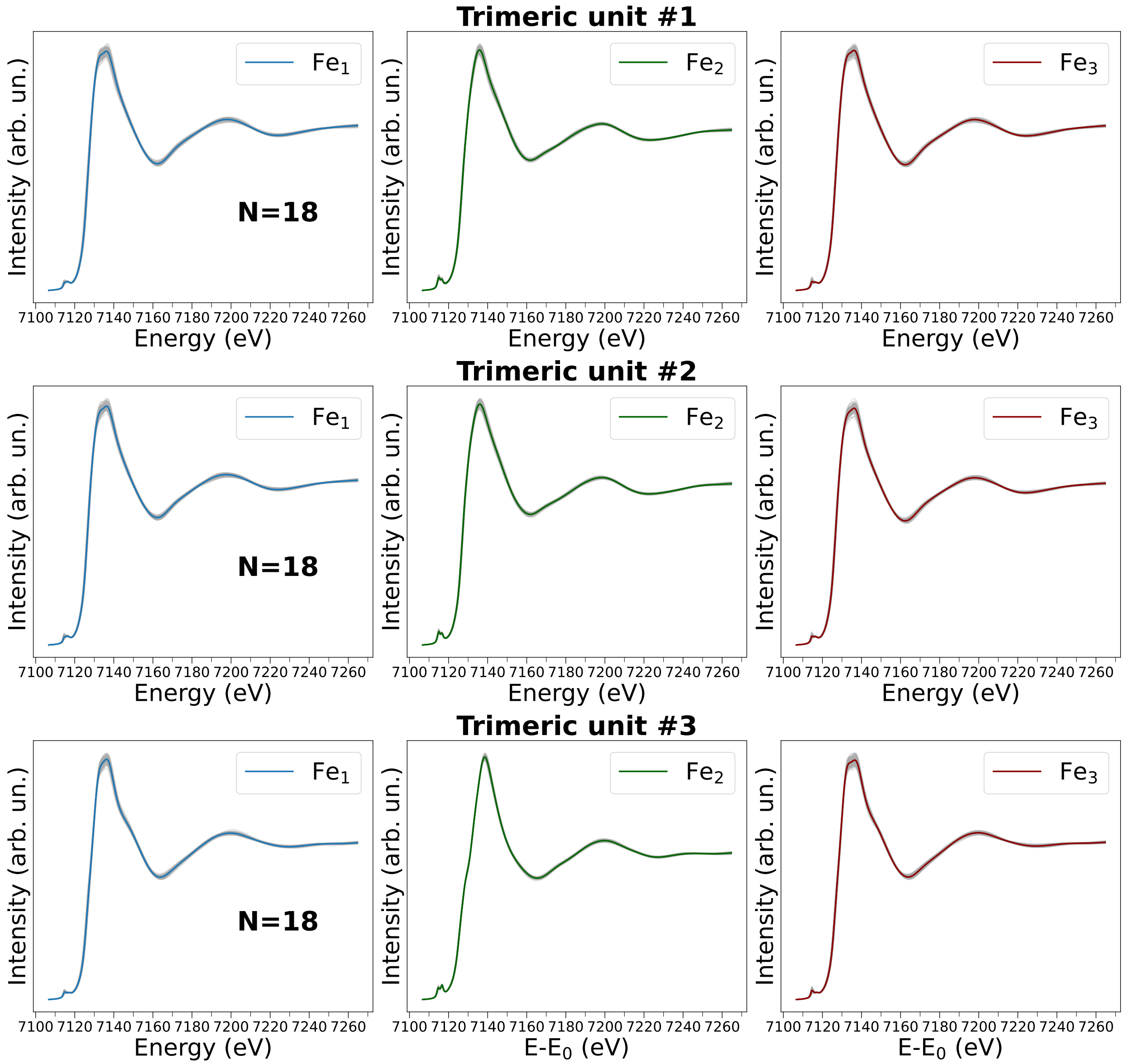

Figure S33: Theoretical Fe K-edge XANES spectra (gray lines) calculated from 100 MD snapshots of MIL-100(Fe) loaded with N=18 water molecules per trimeric unit and converged XANES averages of the 100 spectra (full lines). The three rows display the XANES spectra evaluated for three distinct trimeric units in the MOF unit cell. The three columns display the XANES spectra calculated for each individual Fe site in the given trimeric unit (Fe_1/Fe_3 and Fe_2 correspond to the Fe sites directly coordinated by water and hydroxyl ligands, respectively).

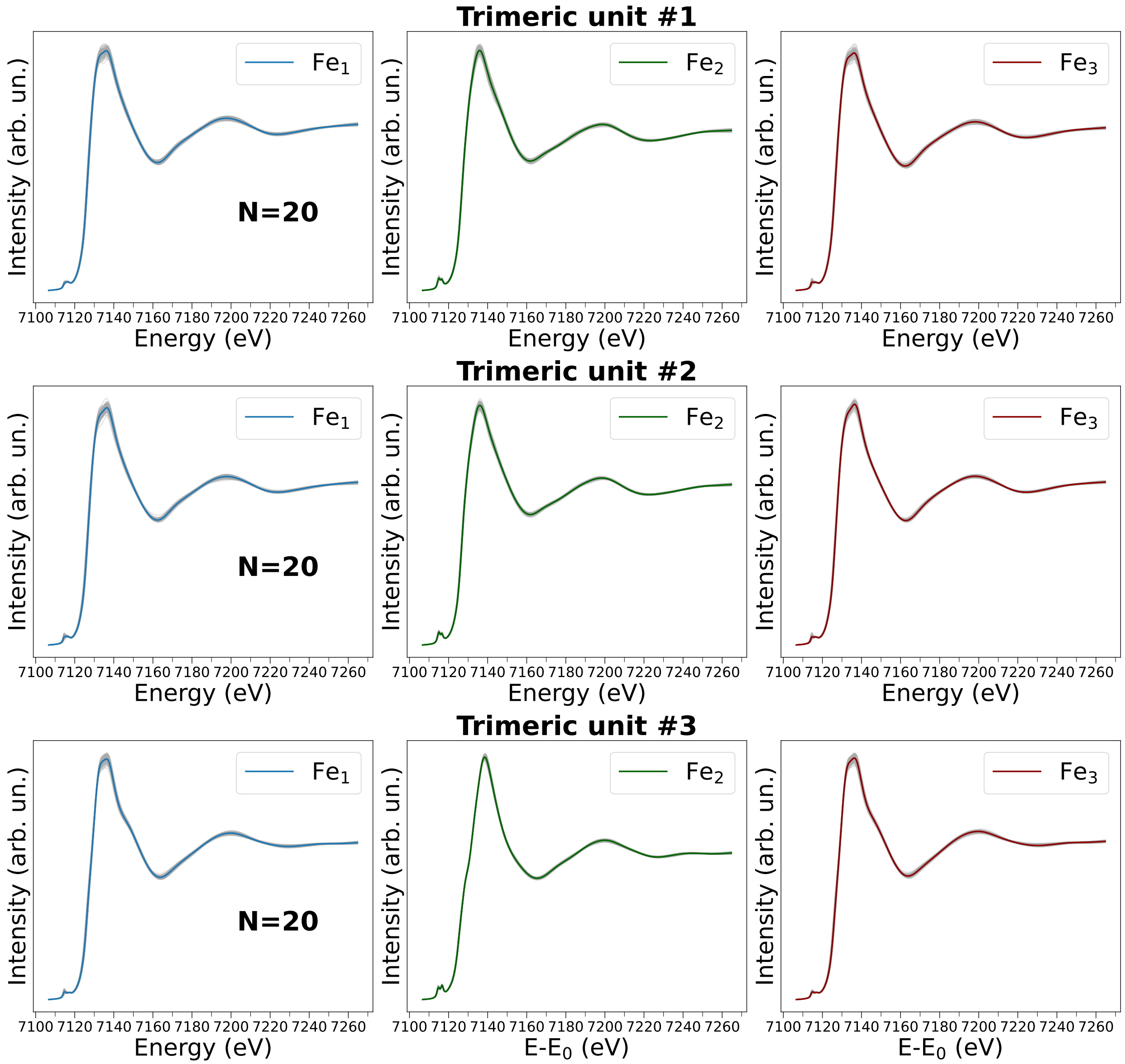

Figure S34: Theoretical Fe K-edge XANES spectra (gray lines) calculated from 100 MD snapshots of MIL-100(Fe) loaded with N=20 water molecules per trimeric unit and converged XANES averages of the 100 spectra (full lines). The three rows display the XANES spectra evaluated for three distinct trimeric units in the MOF unit cell. The three columns display the XANES spectra calculated for each individual Fe site in the given trimeric unit (Fe_1/Fe_3 and Fe_2 correspond to the Fe sites directly coordinated by water and hydroxyl ligands, respectively).

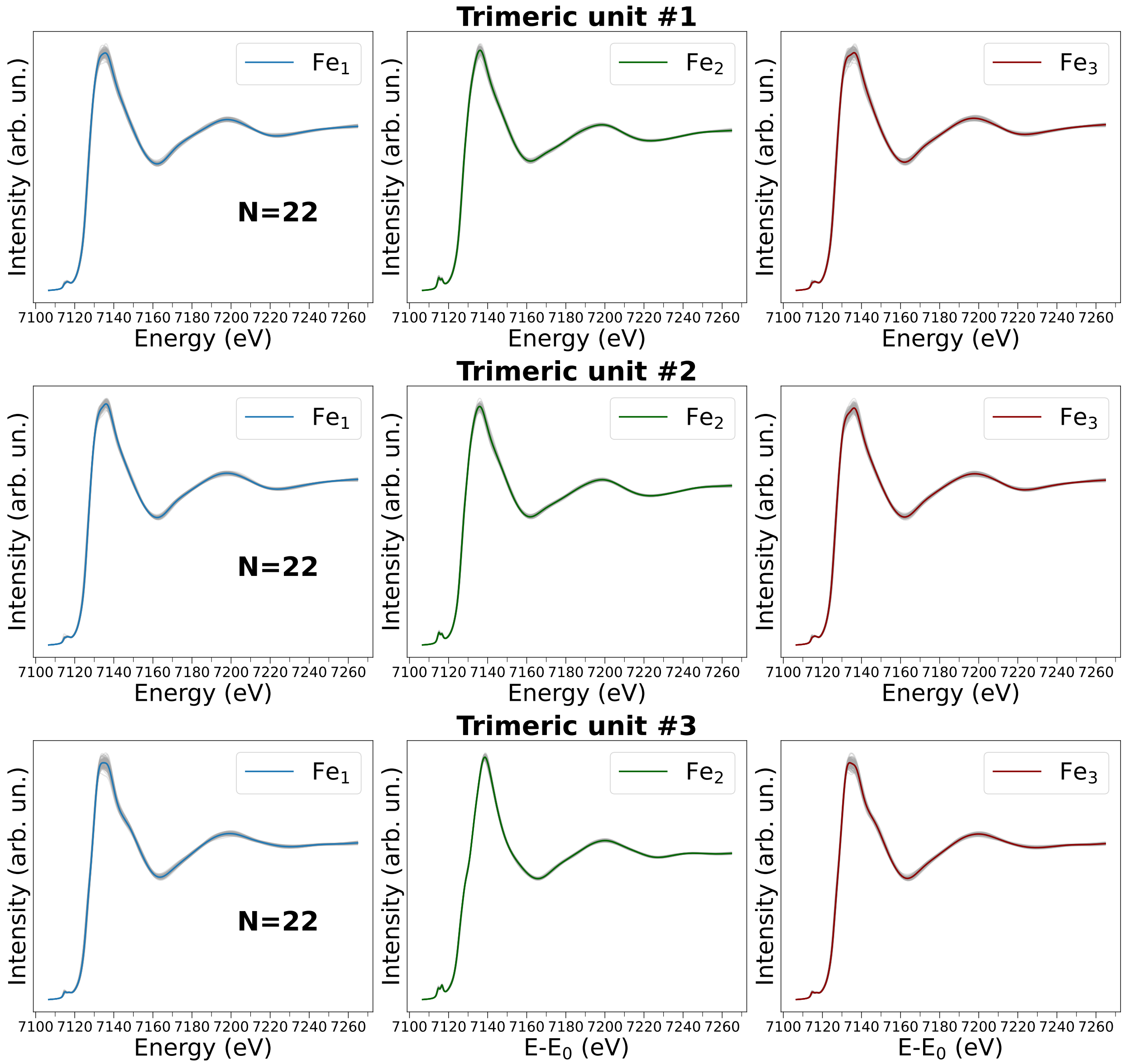

Figure S35: Theoretical Fe K-edge XANES spectra (gray lines) calculated from 100 MD snapshots of MIL-100(Fe) loaded with N=22 water molecules per trimeric unit and converged XANES averages of the 100 spectra (full lines). The three rows display the XANES spectra evaluated for three distinct trimeric units in the MOF unit cell. The three columns display the XANES spectra calculated for each individual Fe site in the given trimeric unit (Fe_1/Fe_3 and Fe_2 correspond to the Fe sites directly coordinated by water and hydroxyl ligands, respectively).

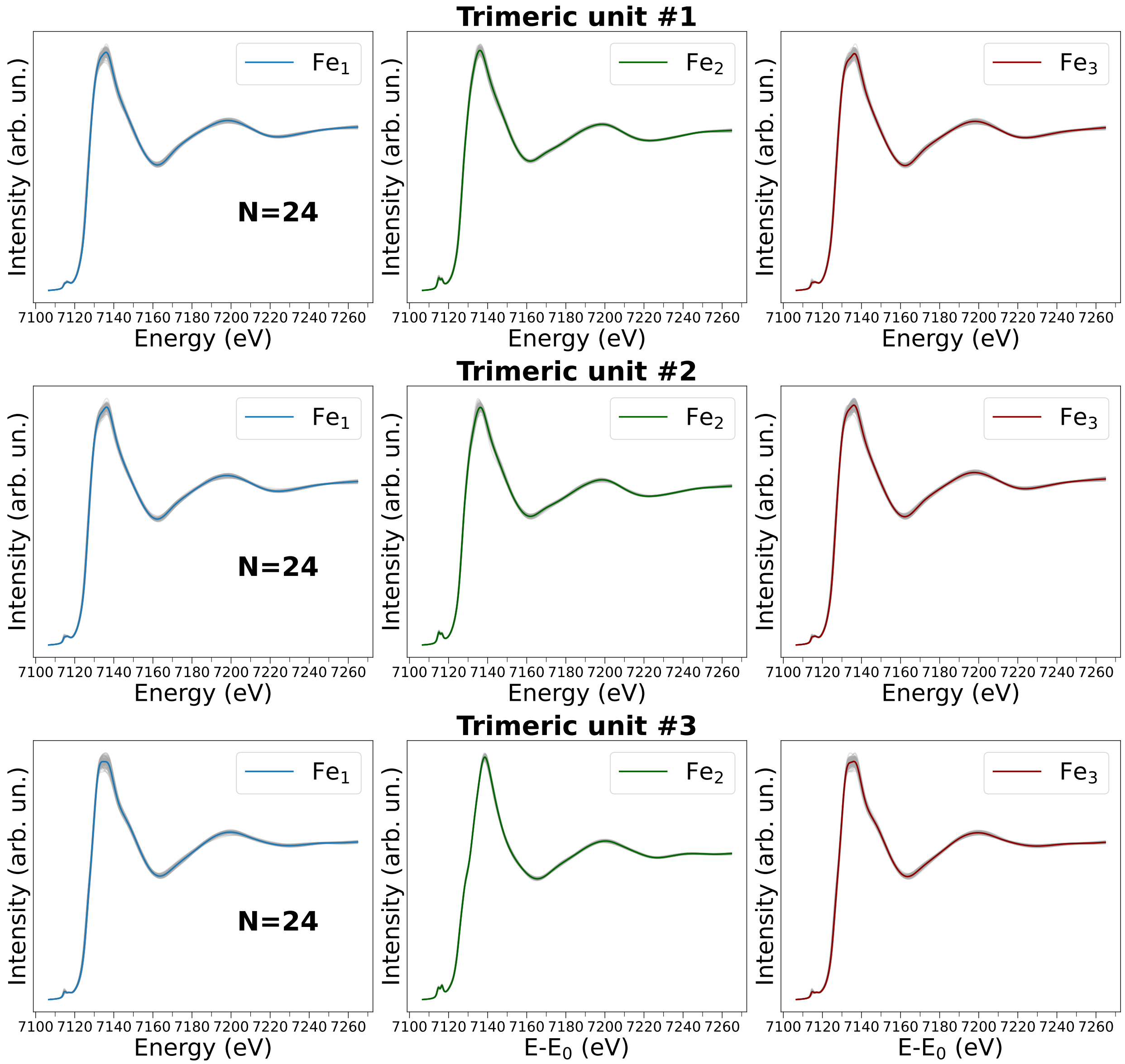

Figure S36: Theoretical Fe K-edge XANES spectra (gray lines) calculated from 100 MD snapshots of MIL-100(Fe) loaded with N=24 water molecules per trimeric unit and converged XANES averages of the 100 spectra (full lines). The three rows display the XANES spectra evaluated for three distinct trimeric units in the MOF unit cell. The three columns display the XANES spectra calculated for each individual Fe site in the given trimeric unit (Fe_1/Fe_3 and Fe_2 correspond to the Fe sites directly coordinated by water and hydroxyl ligands, respectively).

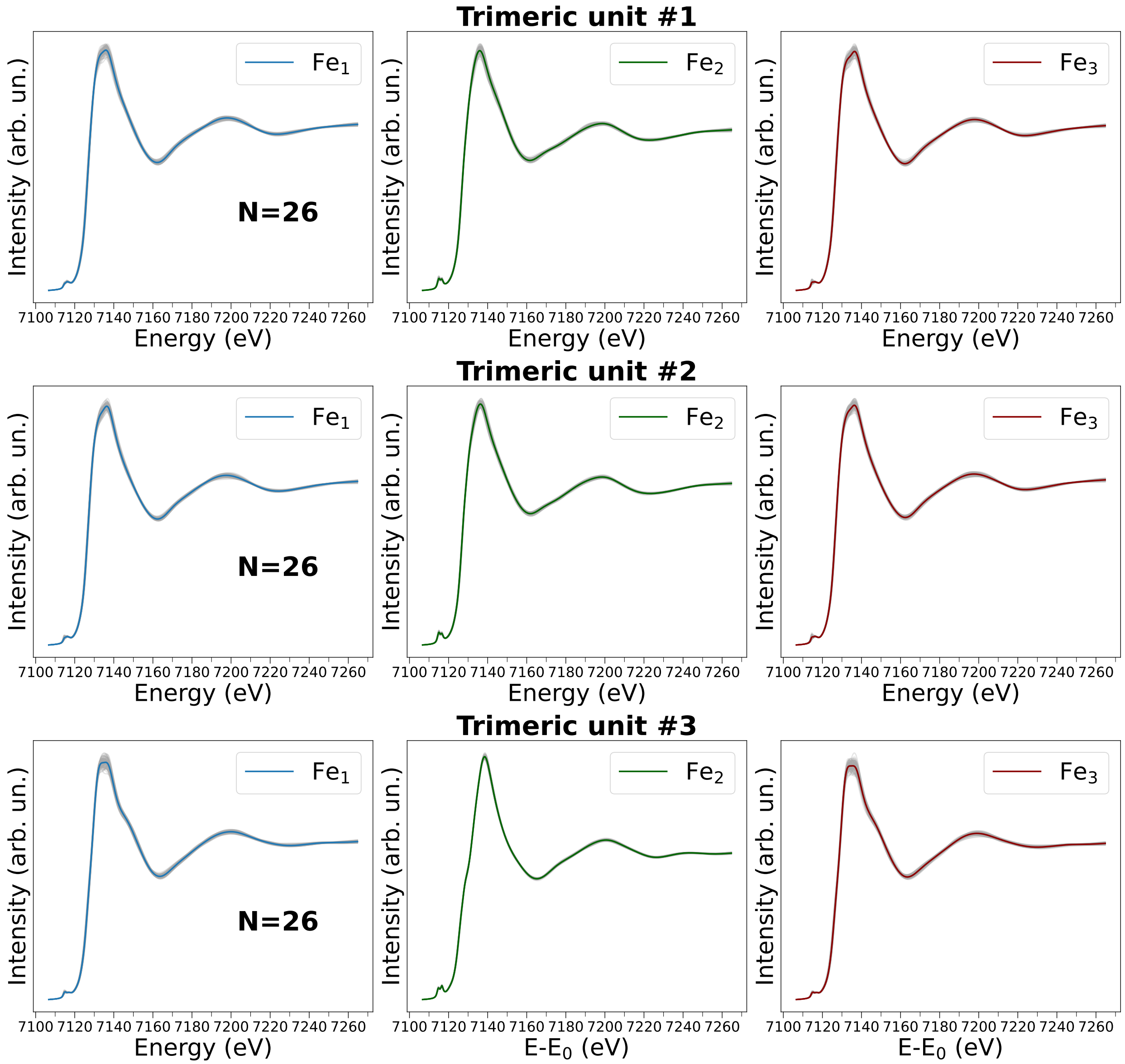

Figure S37: Theoretical Fe K-edge XANES spectra (gray lines) calculated from 100 MD snapshots of MIL-100(Fe) loaded with N=26 water molecules per trimeric unit and converged XANES averages of the 100 spectra (full lines). The three rows display the XANES spectra evaluated for three distinct trimeric units in the MOF unit cell. The three columns display the XANES spectra calculated for each individual Fe site in the given trimeric unit (Fe_1/Fe_3 and Fe_2 correspond to the Fe sites directly coordinated by water and hydroxyl ligands, respectively).

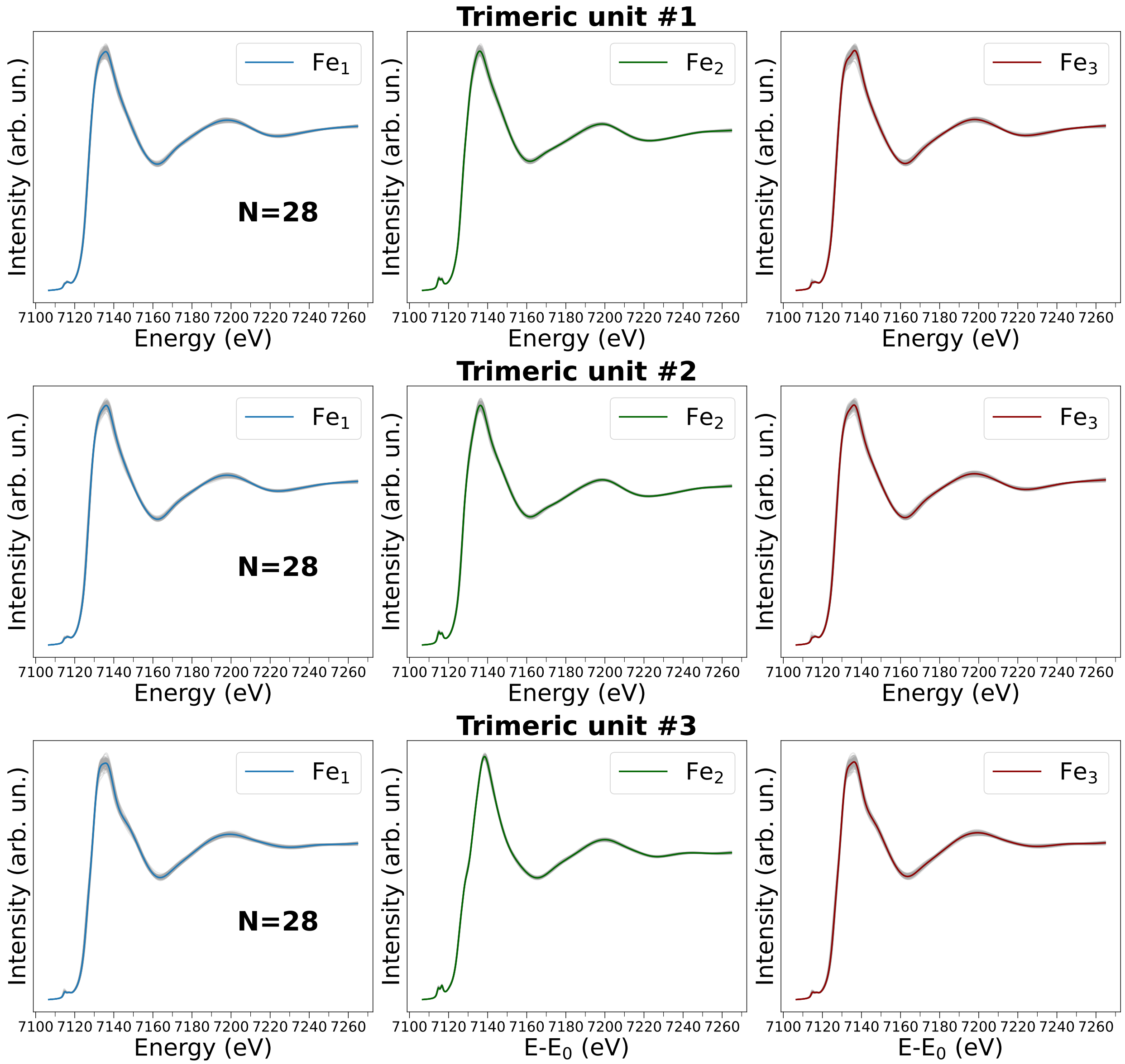

Figure S38: Theoretical Fe K-edge XANES spectra (gray lines) calculated from 100 MD snapshots of MIL-100(Fe) loaded with N=28 water molecules per trimeric unit and converged XANES averages of the 100 spectra (full lines). The three rows display the XANES spectra evaluated for three distinct trimeric units in the MOF unit cell. The three columns display the XANES spectra calculated for each individual Fe site in the given trimeric unit (Fe_1/Fe_3 and Fe_2 correspond to the Fe sites directly coordinated by water and hydroxyl ligands, respectively).

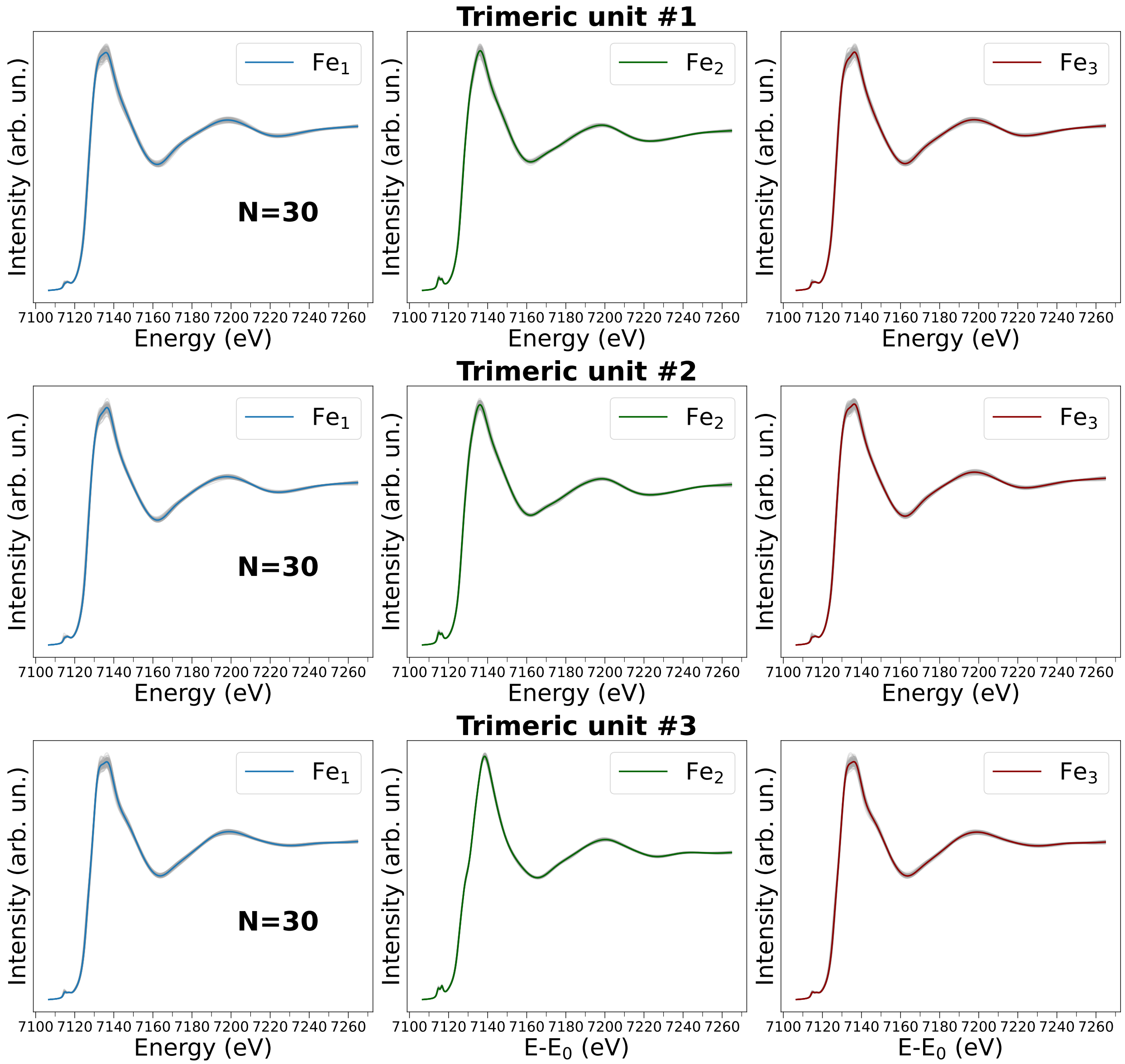

Figure S39: Theoretical Fe K-edge XANES spectra (gray lines) calculated from 100 MD snapshots of MIL-100(Fe) loaded with N=30 water molecules per trimeric unit and converged XANES averages of the 100 spectra (full lines). The three rows display the XANES spectra evaluated for three distinct trimeric units in the MOF unit cell. The three columns display the XANES spectra calculated for each individual Fe site in the given trimeric unit (Fe_1/Fe_3 and Fe_2 correspond to the Fe sites directly coordinated by water and hydroxyl ligands, respectively).

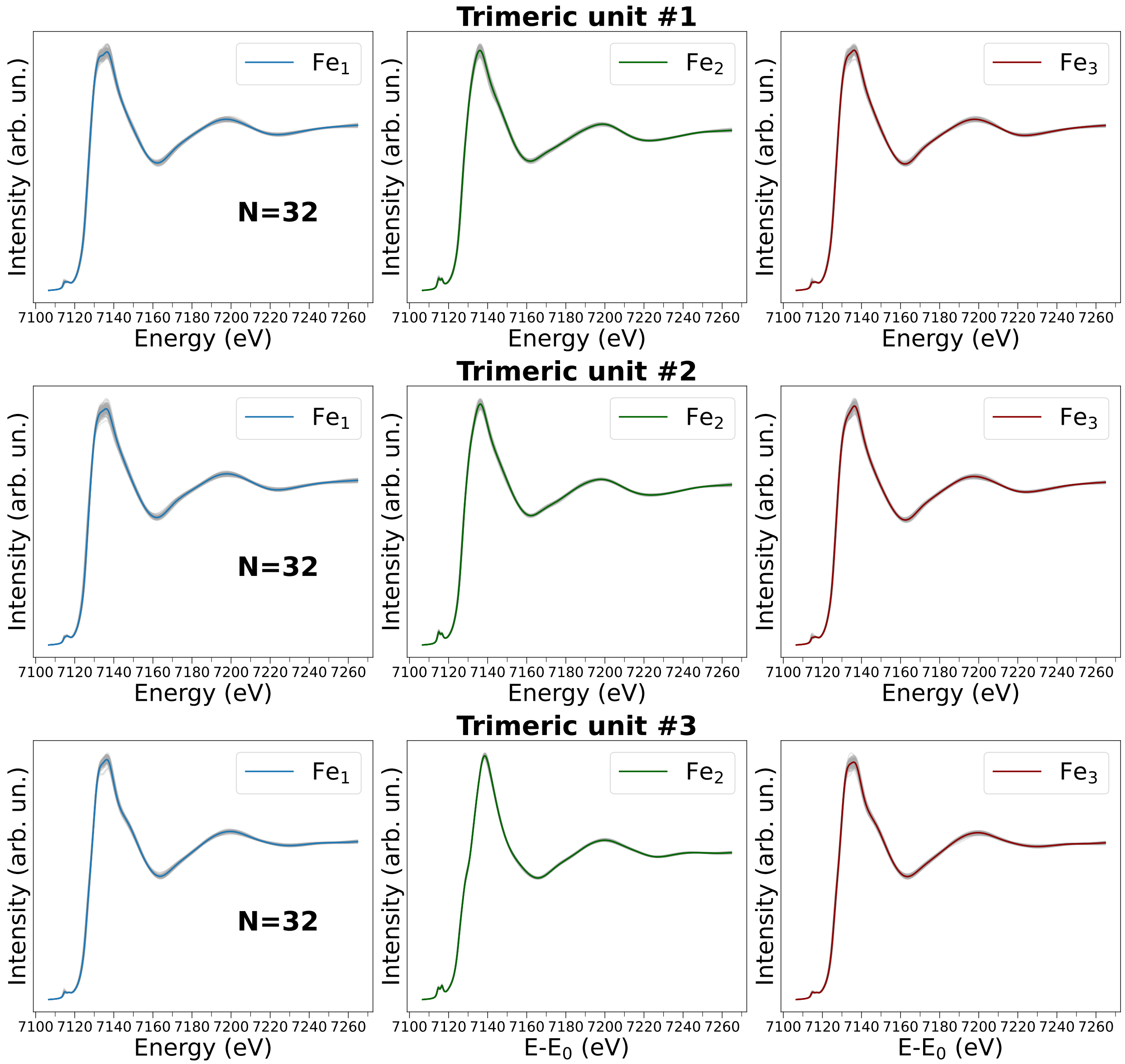

Figure S40: Theoretical Fe K-edge XANES spectra (gray lines) calculated from 100 MD snapshots of MIL-100(Fe) loaded with N=32 water molecules per trimeric unit and converged XANES averages of the 100 spectra (full lines). The three rows display the XANES spectra evaluated for three distinct trimeric units in the MOF unit cell. The three columns display the XANES spectra calculated for each individual Fe site in the given trimeric unit (Fe_1/Fe_3 and Fe_2 correspond to the Fe sites directly coordinated by water and hydroxyl ligands, respectively).

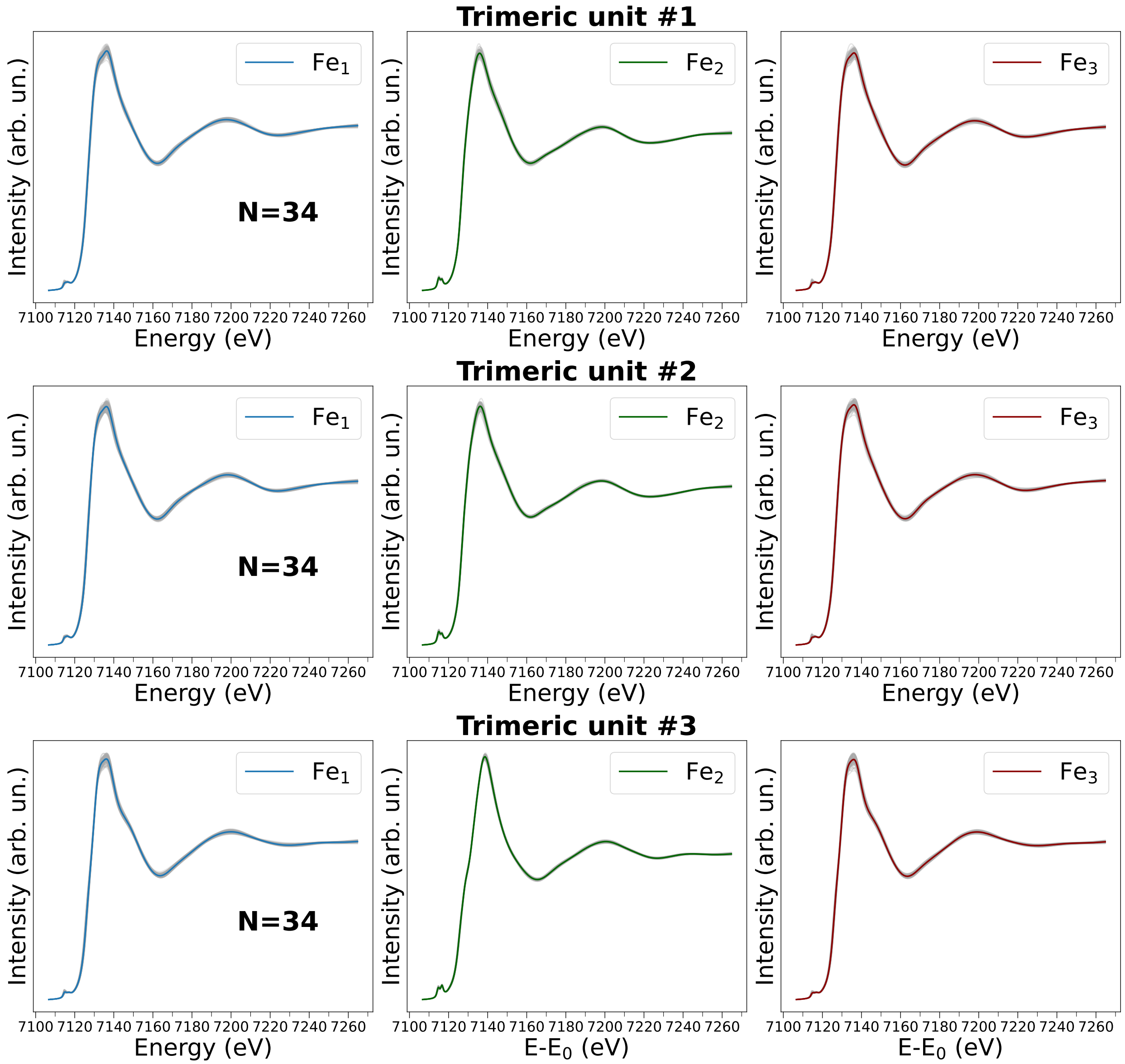

Figure S41: Theoretical Fe K-edge XANES spectra (gray lines) calculated from 100 MD snapshots of MIL-100(Fe) loaded with N=34 water molecules per trimeric unit and converged XANES averages of the 100 spectra (full lines). The three rows display the XANES spectra evaluated for three distinct trimeric units in the MOF unit cell. The three columns display the XANES spectra calculated for each individual Fe site in the given trimeric unit (Fe_1/Fe_3 and Fe_2 correspond to the Fe sites directly coordinated by water and hydroxyl ligands, respectively).

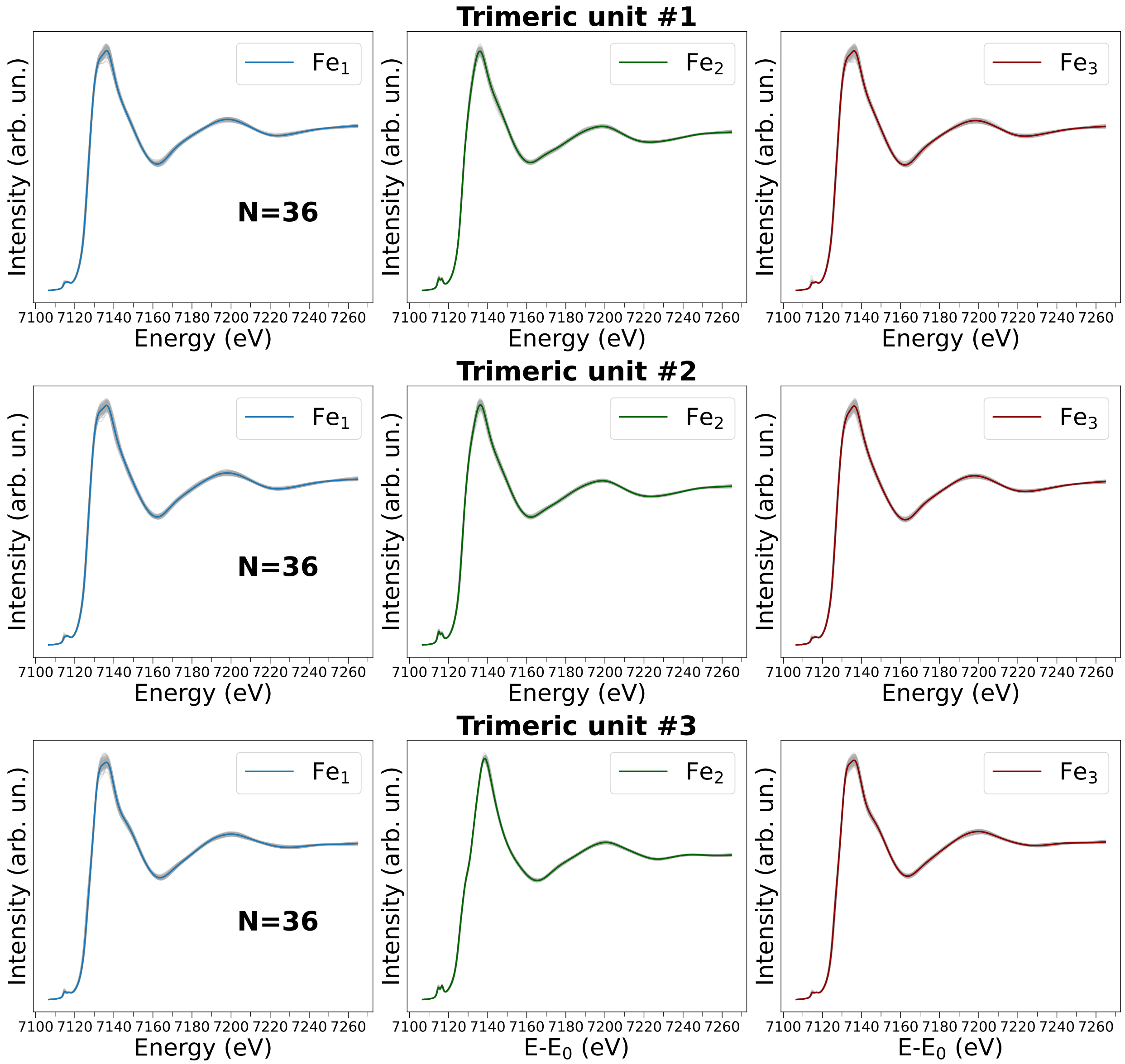

Figure S42: Theoretical Fe K-edge XANES spectra (gray lines) calculated from 100 MD snapshots of MIL-100(Fe) loaded with N=36 water molecules per trimeric unit and converged XANES averages of the 100 spectra (full lines). The three rows display the XANES spectra evaluated for three distinct trimeric units in the MOF unit cell. The three columns display the XANES spectra calculated for each individual Fe site in the given trimeric unit (Fe_1/Fe_3 and Fe_2 correspond to the Fe sites directly coordinated by water and hydroxyl ligands, respectively).

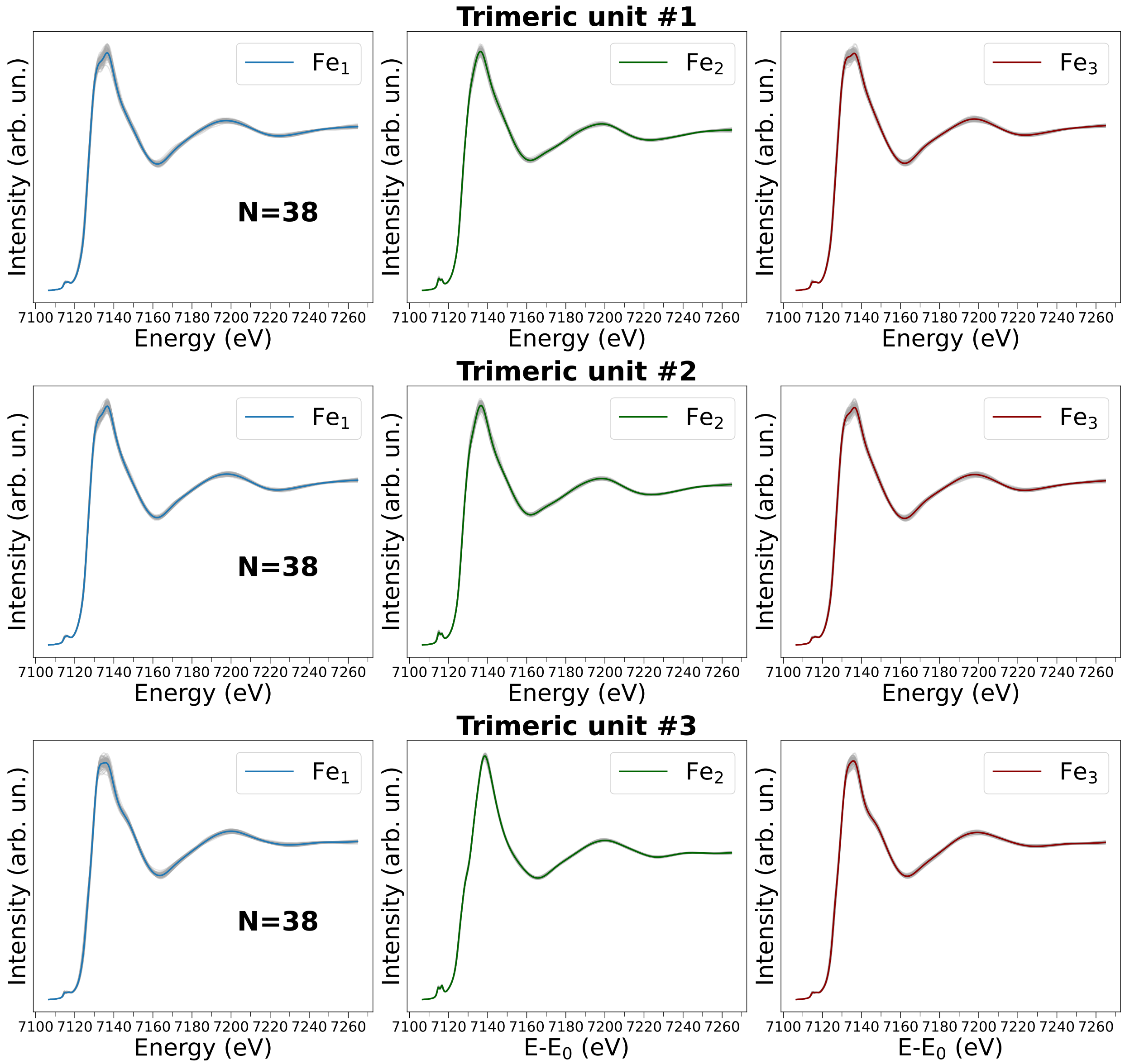

Figure S43: Theoretical Fe K-edge XANES spectra (gray lines) calculated from 100 MD snapshots of MIL-100(Fe) loaded with N=38 water molecules per trimeric unit and converged XANES averages of the 100 spectra (full lines). The three rows display the XANES spectra evaluated for three distinct trimeric units in the MOF unit cell. The three columns display the XANES spectra calculated for each individual Fe site in the given trimeric unit (Fe_1/Fe_3 and Fe_2 correspond to the Fe sites directly coordinated by water and hydroxyl ligands, respectively).

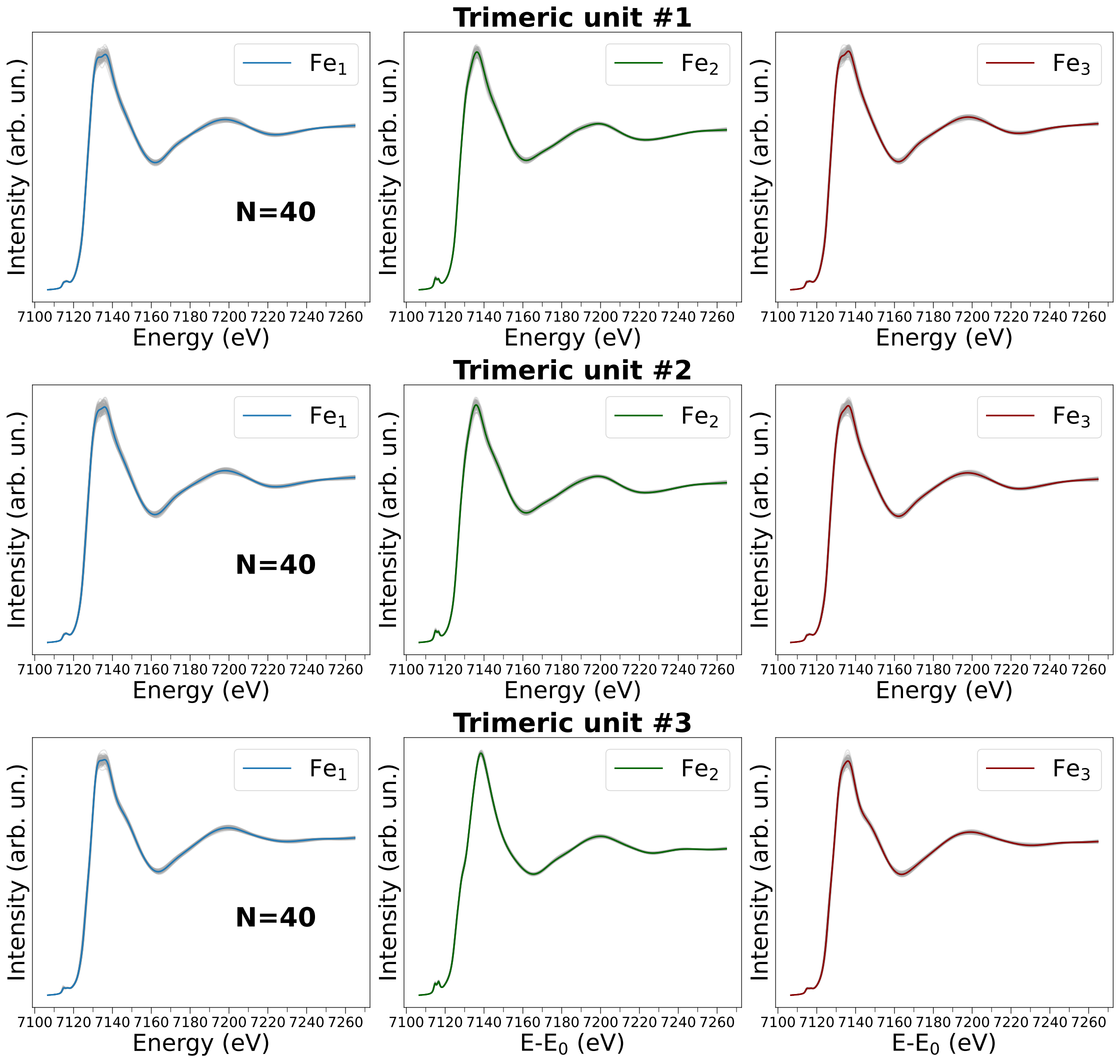

Figure S44: Theoretical Fe K-edge XANES spectra (gray lines) calculated from 100 MD snapshots of MIL-100(Fe) loaded with N=40 water molecules per trimeric unit and converged XANES averages of the 100 spectra (full lines). The three rows display the XANES spectra evaluated for three distinct trimeric units in the MOF unit cell. The three columns display the XANES spectra calculated for each individual Fe site in the given trimeric unit (Fe_1/Fe_3 and Fe_2 correspond to the Fe sites directly coordinated by water and hydroxyl ligands, respectively).

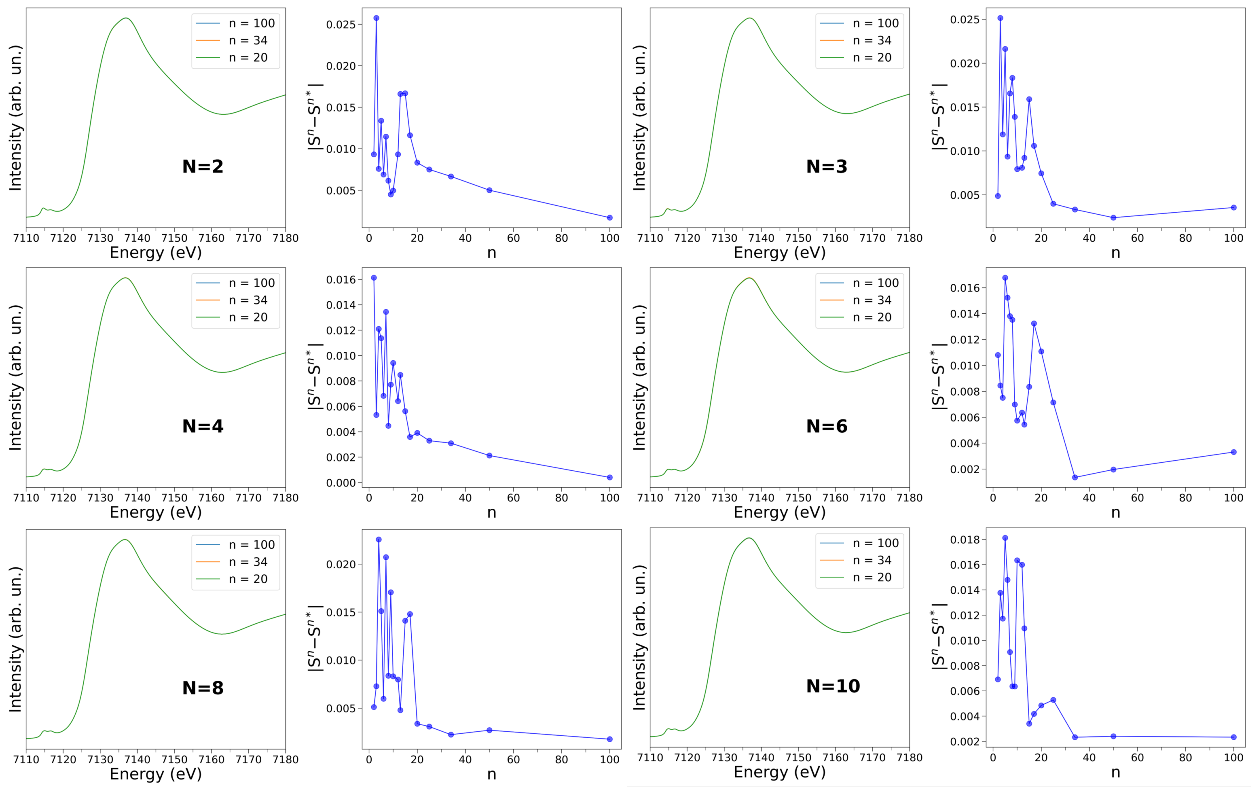

Figure S45: (Left panels) Selection of average Fe K-edge XANES theoretical spectra calculated with an increasing number (n) of spectra from MD snapshots of MIL-100(Fe) loaded with N=2,3,4,6,8,10 water molecules per trimeric unit. (Right panels) Total differences between averages of spectra computed with increasing n values. S^n and S^{n*} are the theoretical averages of n and n* spectra, respectively, with n* immediately preceding the given value of n in the considered sequence.

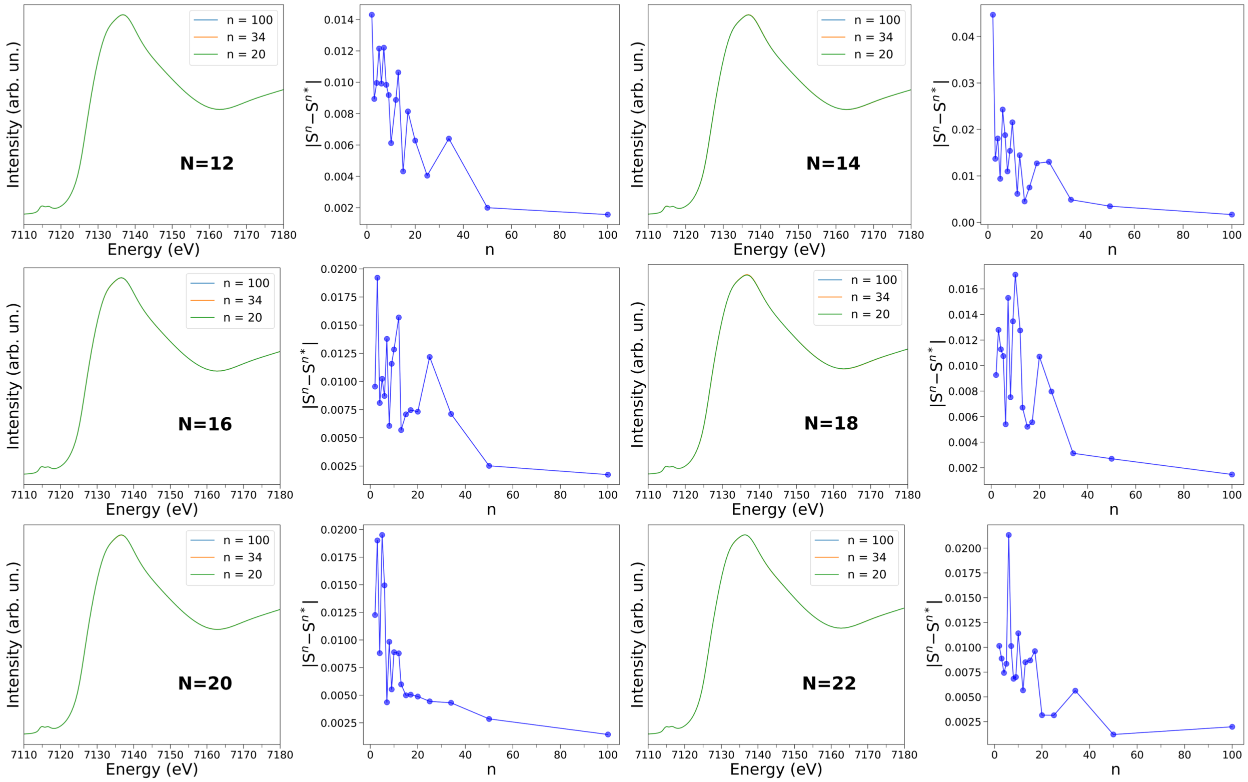

Figure S46: (Left panels) Selection of average Fe K-edge XANES theoretical spectra calculated with an increasing number (n) of spectra from MD snapshots of MIL-100(Fe) loaded with N=12,14,16,18,20,22 water molecules per trimeric unit. (Right panels) Total differences between averages of spectra computed with increasing n values. S^n and S^{n*} are the theoretical averages of n and n* spectra, respectively, with n* immediately preceding the given value of n in the considered sequence.

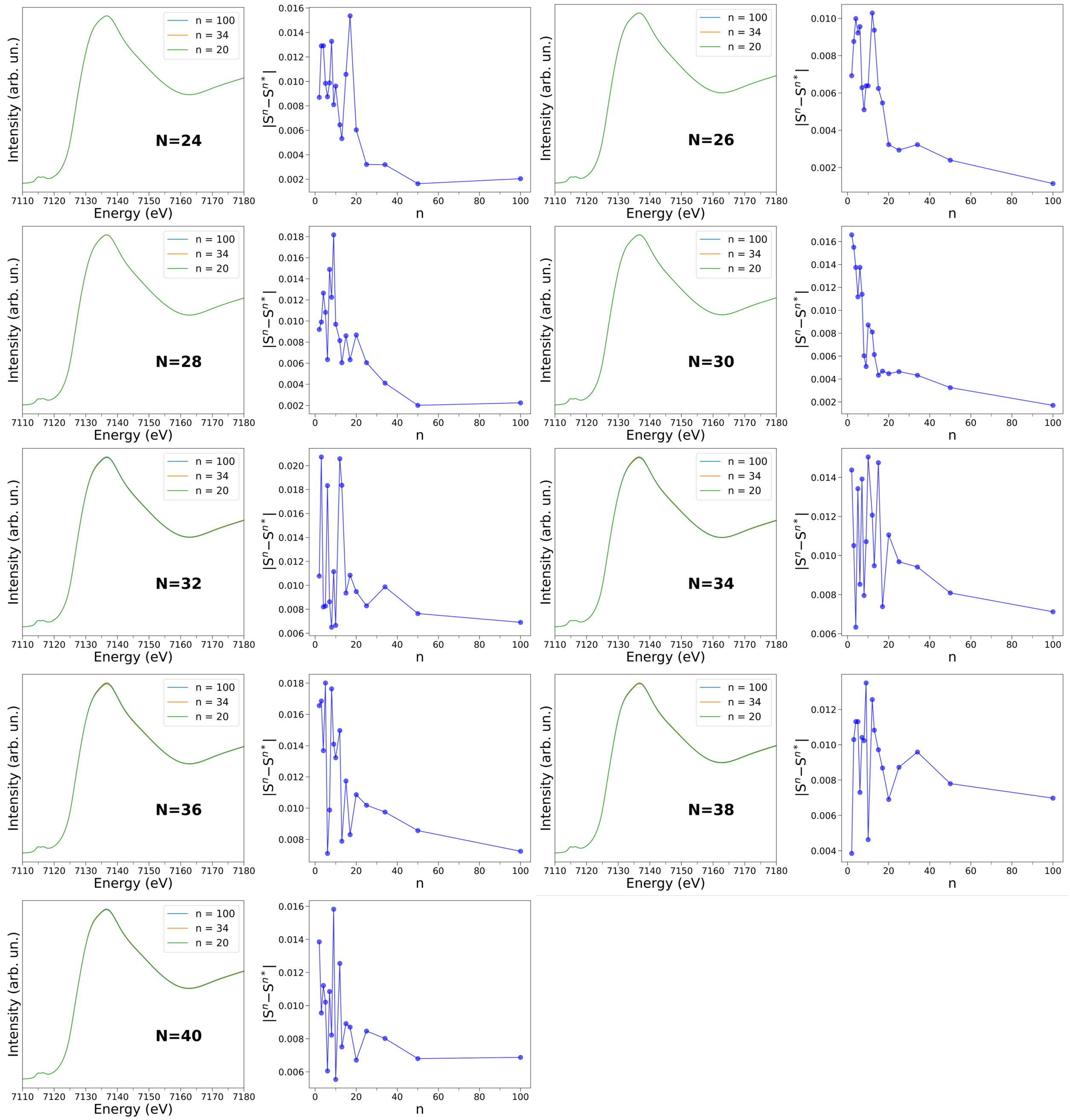

Figure S47: (Left panels) Selection of average Fe K-edge XANES theoretical spectra calculated with an increasing number (n) of spectra from MD snapshots of MIL-100(Fe) loaded with N=24,26,28,30,32,34,36,38,40 water molecules per trimeric unit. (Right panels) Total differences between averages of spectra computed with increasing n values. S^n and S^{n*} are the theoretical averages of n and n* spectra, respectively, with n* immediately preceding the given value of n in the considered sequence.

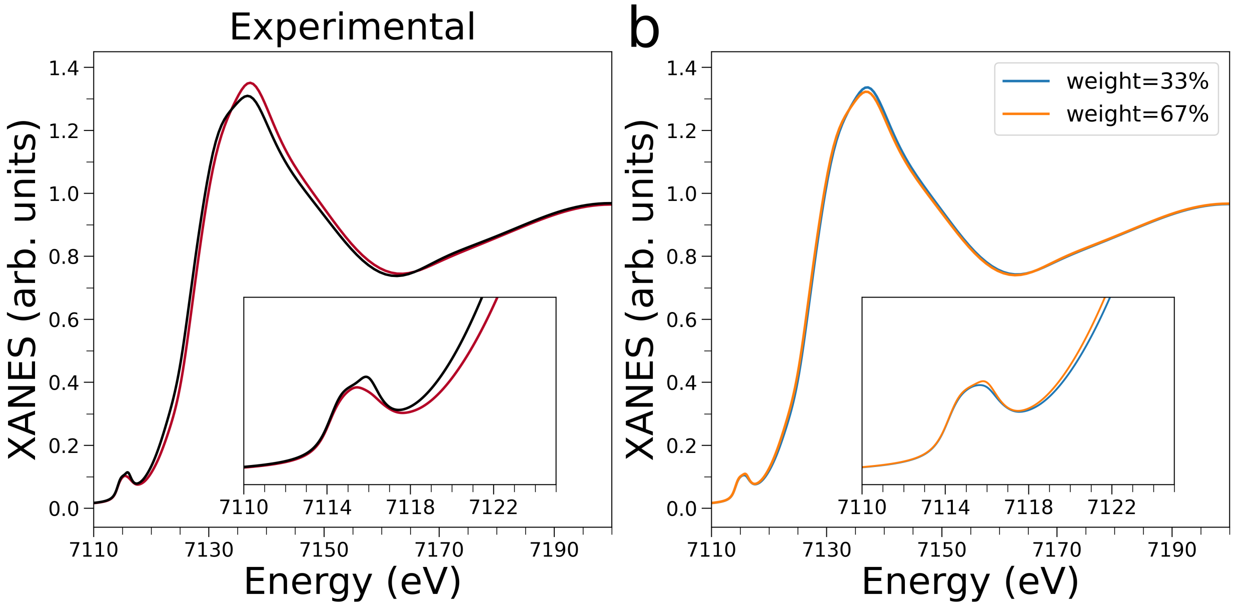

Figure S48: (a) Theoretical Fe K-edge XAS spectra calculated starting from the dehydrated and dehydrated/dehydroxylated MOF structures (red and black curves, respectively). (b) Weighted averages (blue and orange curves) of the theoretical Fe K-edge XAS spectra calculated starting from the dehydrated and dehydrated/dehydroxylated MOF structures. The percentage weights in the spectral sums of the XAS spectrum of the dehydrated/dehydroxylated MOF are listed in the legend. The theoretical XAS spectra calculated starting from the dehydrated and dehydrated/dehydroxylated MOF structures are shown in faded colors to aid the comparison.

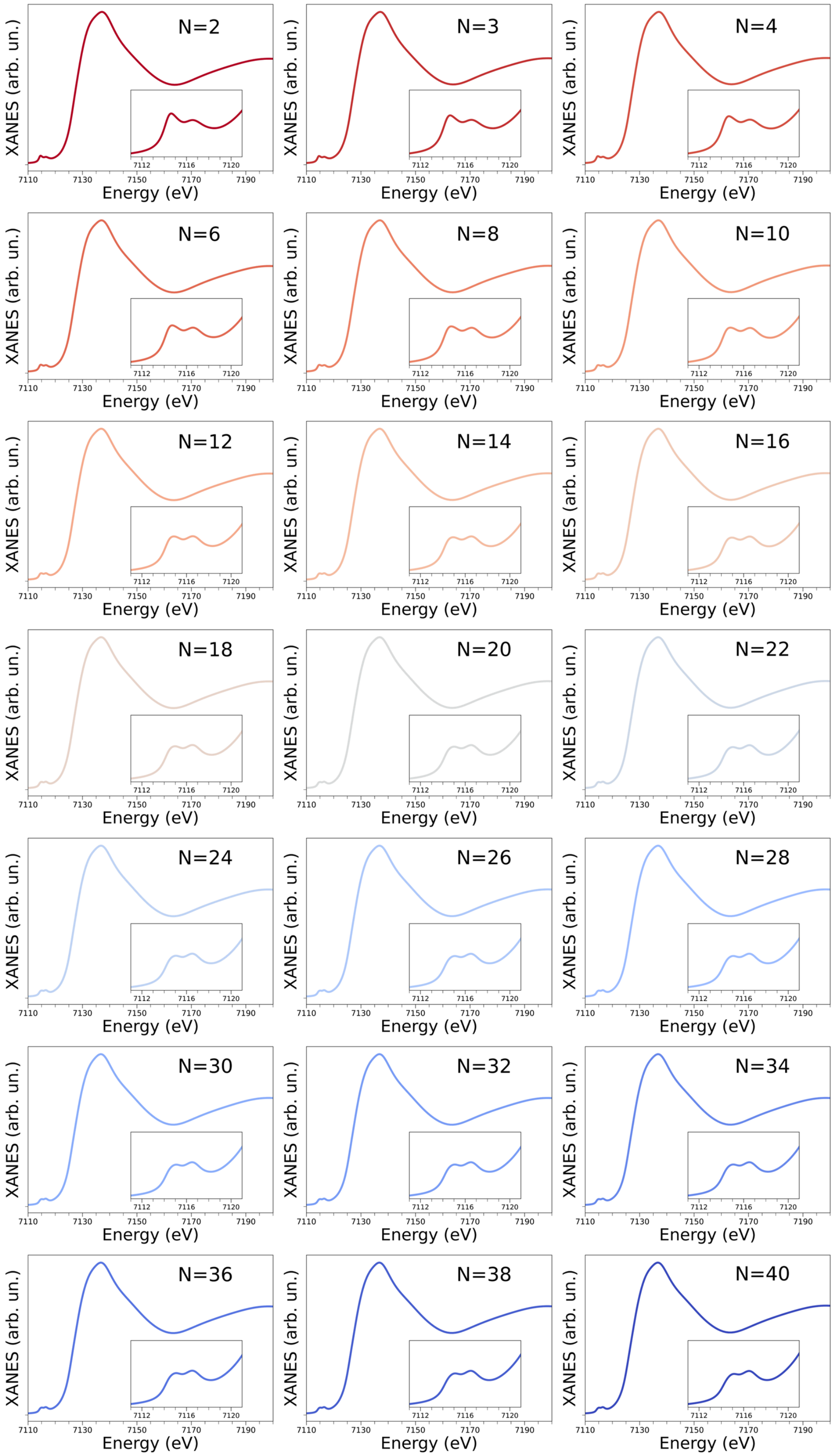

Figure S49: Theoretical Fe K-edge XAS converged average spectra calculated from MD simulations of MIL-100(Fe) loaded with N water molecules per trimeric unit. For each water loading, the corresponding XAS spectrum has been calculated as the average over 20 MD snapshots and 94 distinct trimeric units.

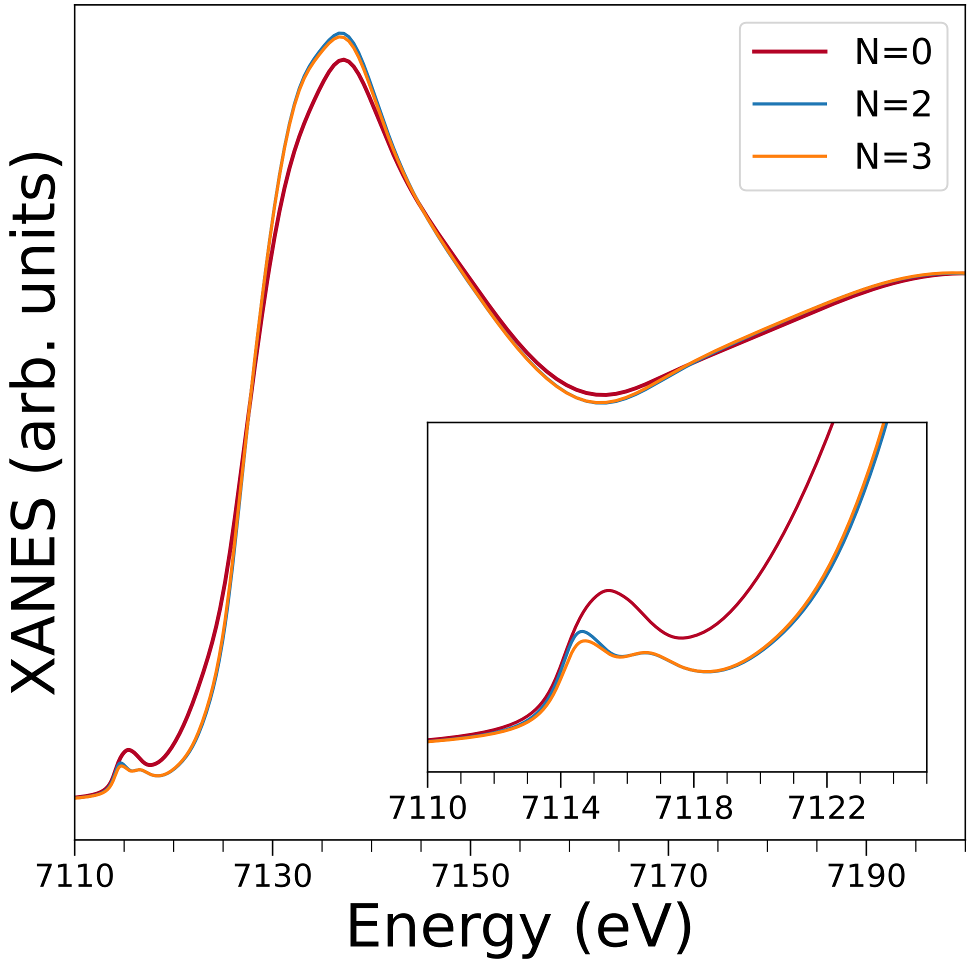

Figure S50: Theoretical Fe K-edge XAS spectra calculated from MD simulations of MIL-100(Fe) loaded with N=0,2,3 water molecules per trimeric unit.

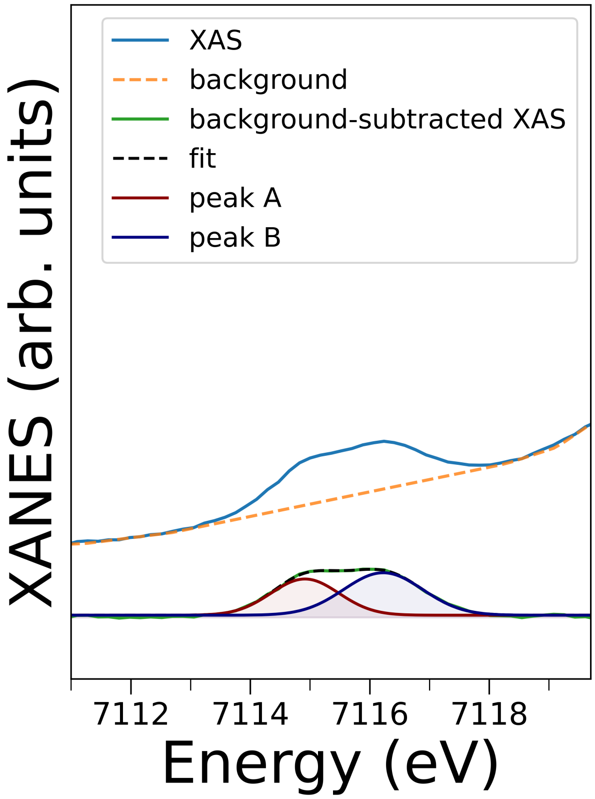

Figure S51: Pre-edge peak fitting of the experimental XAS spectrum of hydrated MIL-100(Fe).

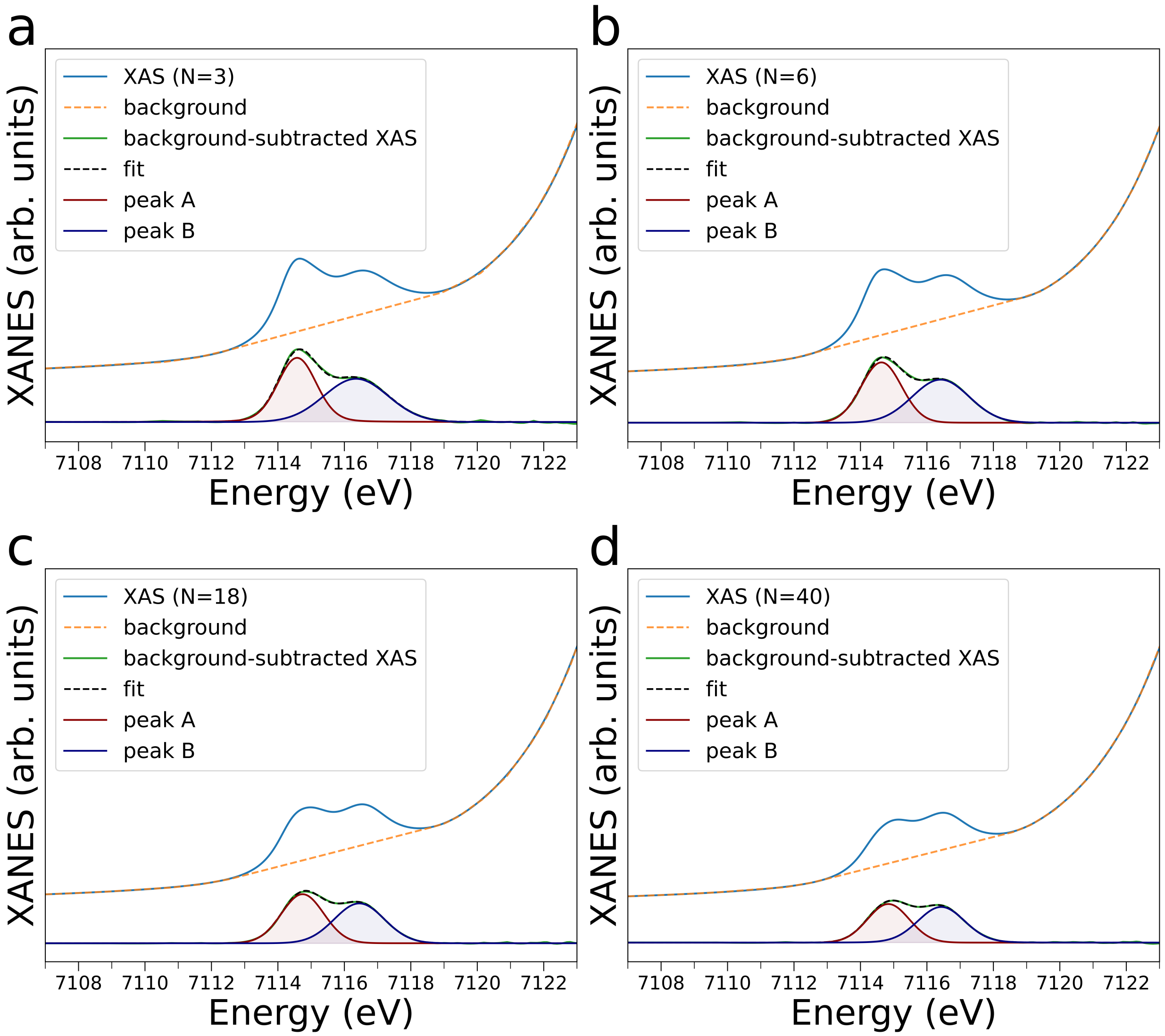

Figure S52: Pre-edge peak fitting of the theoretical XAS spectra calculated from MD simulations of MIL-100(Fe) loaded with N=3 (a), 6 (b), 18 (c), 40 (d) water molecules per trimeric unit.

3 Supplementary Tables (Tables S1–S4)

Table S1: Temperature evolution of the refined unit cell parameters of pristine (scan 1), thermally activated (scans 2-8), hydrated (scan 9-10), and thermally dehydrated (scans 11-15) MIL-100(Fe).

**Scan Number**	**T (°C)**	**Atmosphere**	$\mathbf{R}_{wp}$	**Spacegroup**	**a (Å)**	**Volume (Å^3)**
1	25	Air	3.111	$R\bar{3}$	73.080(1)	390300(16)
2	25	He	5.416	$R\bar{3}$	72.941(2)	388082(32)
3	50	He	5.332	$R\bar{3}$	72.788(2)	385632(34)
4	100	He	4.802	$R\bar{3}$	72.613(2)	382856(32)
5	150	He	4.929	$R\bar{3}$	72.492(2)	380948(34)
6	200	He	4.994	$R\bar{3}$	72.418(2)	379781(37)
7	200 (2 h)	He	4.718	$R\bar{3}$	72.529(2)	381528(36)
8	25	He	4.505	$R\bar{3}$	72.785(2)	385586(34)
9	25	Wet He	2.294	$R\bar{3}$	73.448(1)	396215(16)
10	25	He	4.785	$R\bar{3}$	73.084(2)	390358(34)
11	50	He	4.573	$R\bar{3}$	72.878(2)	387076(33)
12	75	He	4.469	$R\bar{3}$	72.831(2)	386316(32)
13	100	He	4.433	$R\bar{3}$	72.720(2)	384554(33)
14	150	He	4.685	$R\bar{3}$	72.645(2)	383370(36)
15	200	He	4.400	$R\bar{3}$	72.523(2)	381448(37)

Table S2: List of the carried out MD simulations of MIL-100(Fe) at increasing water loadings.

**Water loading**	**Number of atoms**	**Number of water molecules**
N=2	13056	544
N=3	13872	816
N=4	14688	1088
N=6	16320	1632
N=8	17952	2176
N=10	19584	2720
N=12	21216	3264
N=14	22848	3808
N=16	24480	4352
N=18	26112	4896
N=20	27744	5440
N=22	29376	5984
N=24	31008	6528
N=26	32640	7072
N=28	34272	7616
N=30	35904	8160
N=32	37536	8704
N=34	39168	9248
N=36	40800	9792
N=38	42432	10336
N=40	44064	10880

Table S3: Fe-O_{water} bond distances obtained from the Fe-O_{water} radial distribution functions calculated for MIL-100(Fe) at the investigated water loadings. The distance corresponding to the maximum of the first peak of the given Fe-O_{water} radial distribution function is reported. The corresponding coordination number (CN) calculation yielded a value of CN=1 for all MD simulations while employing a distance cutoff in all cases equal to 2.5 Å.

**Water loading**	**r (Å)**
N=2	2.082
N=3	2.082
N=4	2.082
N=6	2.082
N=8	2.07
N=10	2.07
N=12	2.07
N=14	2.07
N=16	2.07
N=18	2.07
N=20	2.07
N=22	2.07
N=24	2.07
N=26	2.07
N=28	2.07
N=30	2.07
N=32	2.07
N=34	2.07
N=36	2.07
N=38	2.07
N=40	2.07

Table S4: Parameters of the theoretical XAS spectra extracted from MD simulations performed at water loadings of N = 3, 6, 18, 40 compared to those of the experimental XAS spectrum of hydrated MIL-100(Fe). In particular, the energies (E_A and E_B, eV) and areas (A_A and A_B) of the pre-edge transitions are reported, together with the ratio of the maximum intensities of the pre-edge transitions (I_A/I_B), the energy of the white line transition (E_{wl}, eV), the area of the given XAS spectrum integrated up to the energy of the white line transition (A_{wl}), and the edge energy of the given XAS spectrum (E_{edge}, eV).

**XAS spectrum**	$\mathbf{E}_A$	$\mathbf{E}_B$	$\mathbf{A}_A$	$\mathbf{A}_B$	$\mathbf{I}_{A/B}$	$\mathbf{E}_{wl}$	$\mathbf{A}_{wl}$	$\mathbf{E}_{edge}$
N=3	7114.5	7116.3	0.0564	0.0605	1.104	7137.2	13.30	7126.7
N=6	7114.6	7116.4	0.0569	0.0572	1.051	7137.2	13.36	7126.4
N=18	7114.7	7116.4	0.0521	0.0498	1.047	7136.7	13.42	7126.4
N=40	7114.8	7116.4	0.0408	0.0434	0.942	7136.7	13.48	7125.9
Exp. hydrated MOF	7114.9	7116.2	0.0499	0.0575	0.879	7136.0	14.13	7126.7

References

(S1) Souza, B. E.; Möslein, A. F.; Titov, K.; Taylor, J. D.; Rudić, S.; Tan, J.-C. Green Reconstruction of MIL-100(Fe) in Water for High Crystallinity and Enhanced Guest Encapsulation. *ACS Sustain. Chem. Eng.* **2020**, *8*, 8247–8255.

(S2) Dyadkin, V.; Pattison, P.; Dmitriev, V.; Chernyshov, D. A New Multipurpose Diffractometer PILATUS@ SNBL. *J. Synchr. Rad.* **2016**, *23*, 825–829.

(S3) Juhás, P.; Davis, T.; Farrow, C. L.; Billinge, S. J. PDFgetX3: a Rapid and Highly Automatable Program for Processing Powder Diffraction Data Into Total Scattering Pair Distribution Functions. *J. Appl. Crystallogr.* **2013**, *46*, 560–566.

(S4) Le Bail, A.; Duroy, H.; Fourquet, J. L. Ab-initio Structure Determination of $LiSbWO_6$ by X-ray Powder Diffraction. *Mater. Res. Bull.* **1988**, *23*, 447–452.

(S5) Toby, B. H.; Von Dreele, R. B. GSAS-II: the Genesis of a Modern Open-Source All Purpose Crystallography Software Package. *J. Appl. Crystallogr.* **2013**, *46*, 544–549.

(S6) Gowers, R. J.; Carbone, P. A Multiscale Approach to Model Hydrogen Bonding: The case of Polyamide. *J. Chem. Phys.* **2015**, *142*, 224907.

(S7) Richard J. Gowers,; Max Linke,; Jonathan Barnoud,; Tyler J. E. Reddy,; Manuel N. Melo,; Sean L. Seyler,; Jan Domański,; David L. Dotson,; Sébastien Buchoux,; Ian M. Kenney,; Oliver Beckstein, MDAnalysis: A Python Package for the Rapid Analysis of Molecular Dynamics Simulations. Proceedings of the 15th Python in Science Conference. 2016; pp 98 – 105.

(S8) Michaud-Agrawal, N.; Denning, E. J.; Woolf, T. B.; Beckstein, O. MDAnalysis: A toolkit for the Analysis of Molecular Dynamics Simulations. *J. Comput. Chem.* **2011**, *32*, 2319–2327.

(S9) Terranova, Z. L.; Paesani, F. The Effects of Framework Dynamics on the Behavior of Water Adsorbed in the [Zn(l-L)(Cl)] and Co-MOF-74 Metal–Organic Frameworks. *Phys. Chem. Chem. Phys.* **2016**, *18*, 8196–8204.

(S10) McInnes, L.; Healy, J.; Melville, J. UMAP: Uniform Manifold Approximation and Projection for Dimension Reduction. *ArXiv e-prints* **2018**,

(S11) Becht, E.; McInnes, L.; Healy, J.; Dutertre, C.-A.; Kwok, I. W. H.; Guan Ng, L.; Ginhoux, F.; Newell, E. W. Dimensionality Reduction for Visualizing Single-cell Data Using UMAP. *Nat. Biotechnol.* **2019**, *37*, 38–44.

(S12) Healy, J.; McInnes, L. Uniform Manifold Approximation and Projection. *Nat. Rev. Methods Primers* **2024**, *4*, 82.

(S13) Joly, Y. X-ray Absorption Near-Edge Structure Calculations Beyond the Muffin-Tin Approximation. *Phys. Rev. B* **2001**, *63*, 125120.

(S14) Bunău, O.; Joly, Y. Self-Consistent Aspects of X-ray Absorption Calculations. *J. Phys: Condes. Matter* **2009**, *21*, 345501.

(S15) Hedin, L.; Lundqvist, B. I. Explicit Local Exchange-Correlation Potentials. *J. Phys. C: Solid State Phys.* **1971**, *4*, 2064.

(S16) Joly, Y.; Bunău, O.; Lorenzo, J. E.; Galéra, R. M.; Grenier, S.; Thompson, B. Self-consistency, Spin-orbit and Other Advances in the FDMNES Code to Simulate XANES and RXD Experiments. *Journal of Physics: Conference Series* **2009**, *190*, 012007.

(S17) Rankine, C. D.; Madkhali, M. M. M.; Penfold, T. J. A Deep Neural Network for the Rapid Prediction of X-ray Absorption Spectra. *J. Phys. Chem. A* **2020**, *124*, 4263–4270.
